# Chronic Intermittent Hypoxia Reduces the Effects of Glucosteroid in Asthma via Activating the p38 MAPK Signaling Pathway

**DOI:** 10.3389/fphys.2021.703281

**Published:** 2021-08-27

**Authors:** Li Liang, Xin Gu, Hai Ji Shen, Yu Heng Shi, Yao Li, Jie Zhang, Yan Yan Chen, Zhen He Chen, Jia Yun Ma, Qing Yun Li

**Affiliations:** ^1^Department of Respiratory and Critical Care Medicine, Shanghai Ninth People’s Hospital, Shanghai Jiao Tong University School of Medicine, Shanghai, China; ^2^Department of Respiratory and Critical Care Medicine, Shanghai General Hospital, Shanghai Jiao Tong University School of Medicine, Shanghai, China; ^3^Department of Urology, Shanghai Ninth People’s Hospital, Shanghai Jiao Tong University School of Medicine, Shanghai, China; ^4^Department of Respiratory and Critical Care Medicine, Ruijin Hospital, Shanghai Jiao Tong University School of Medicine, Shanghai, China

**Keywords:** p38 MAPK pathway, ovalbumin, chronic intermittent hypoxia, asthma, glucosteroid

## Abstract

**Aims:**

Obstructive sleep apnea (OSA) is a risk factor for steroid-resistant (SR) asthma. However, the underlying mechanism is not well defined. This study aimed to investigate how chronic intermittent hypoxia (CIH), the main pathophysiology of OSA, influenced the effects of glucocorticoids (GCs) on asthma.

**Main Methods:**

The effects of dexamethasone (Dex) were determined using the ovalbumin (OVA)-challenged mouse model of asthma and transforming growth factor (TGF)-β treated airway smooth muscle cells (ASMCs), with or without CIH. The p38 MAPK signaling pathway activity was then detected in the mouse (*n* = 6) and ASMCs models (*n* = 6), which were both treated with the p38 MAPK inhibitor SB239063.

**Key Findings:**

Under CIH, mouse pulmonary resistance value, inflammatory cells in bronchoalveolar lavage fluid (BALF), and inflammation scores increased in OVA-challenged combined with CIH exposure mice compared with OVA-challenged mice (*p* < 0.05). These indicators were similarly raised in the OVA + CIH + Dex group compared with the OVA + Dex group (*P* < 0.05). CIH exposure enhanced the activation of the p38 MAPK pathway, oxidative stress injury, and the expression of NF-κB both in lung tissue and ASMCs, which were reversed by treatment with Dex and SB239063. In the *in vitro* study, treatment with Dex and SB239063 decreased ASMCs proliferation induced by TGF-β combined with CIH and suppressed activation of the p38 MAPK pathway, oxidative stress injury, and NF-κB nuclear transcription (*p* < 0.05).

**Significance:**

These results indicated that CIH decreased GC sensitivity by activating the p38 MAPK signaling pathway.

## Introduction

Asthma is a heterogeneous disease characterized by chronic airway inflammation (Boonpiyathad et al., 2019; Lambrecht et al., 2019; McGregor et al., 2019). Hormone insensitive or steroid-resistant (SR) asthma, which accounts for more than 20% of all asthma patients, cannot achieve the expected effect even after receiving large doses of hormone therapy (McManus, 2003; Ramamoorthy and Cidlowski, 2013), which makes the treatment of asthma challenging. Obstructive sleep apnea (OSA) is characterized by chronic intermittent hypoxia (CIH) and sleep fragmentation (Somers et al., 1995), resulting in injury to multiple systems and affecting the quality of life and survival of patients (Tsai, 2017). Asthma combined with OSA is considered a type of overlap syndrome, with a prevalence of 38–70% (Guo et al., 2017). It is noteworthy that the coexistence of asthma and OSA is not a simple superposition of symptoms, but a synergistic effect (Julien et al., 2009; Broytman et al., 2015; Qiao and Xiao, 2015). ten Brinke et al. (2005) confirmed that OSA (adjusted odds ratio [OR] of 3.4) was significantly associated with frequent asthma exacerbations. The previous studies demonstrated that CIH led to bronchial hyperreactivity, increased airway and systemic inflammation, and thus promoted the risk of refractory asthma (Broytman et al., 2015). It has been found that patients with asthma and OSA are more likely to develop SR asthma which is difficult to treat (Prasad et al., 2020).

To date, the mechanism of OSA in SR or refractory asthma is unclear. We previously found that p38 MAPK pathway activation following ozone exposure induced glucocorticoid (GC)-resistance in ovalbumin (OVA)-challenged mice (Liang et al., 2013). Other studies have shown that CIH exposure led to the activation of the p38 MAPK pathway in the nervous system and vascular endothelium (Lee et al., 2016; Liu et al., 2017). It was hypothesized that CIH may induce steroid resistance by activating the p38 MAPK signaling pathway. Thus, we examined the effect of CIH on the sensitivity of GCs on airway inflammation and the possible mechanism of the p38 MAPK pathway using an *in vivo* and *in vitro* model.

## Materials and Methods

### Animal Study

In the study, C57/BL6 male mice (20 ± 2 g) were obtained from SLAC Laboratory Animal Co. Ltd. (Shanghai, China) and housed under specific pathogen-free conditions. This study was carried out in strict accordance with the Guide for the Care and Use of Laboratory Animals (Eighth Edition, 2011, published by The National Academies Press, 2101 Constitution Ave. NW, Washington, DC, United States). The protocol was reviewed and approved by the Shanghai Ninth People’s Hospital Institutional Review Board (Permit Number: HKDL2017265), and all animal experiments were conducted in Shanghai Ninth People’s Hospital. All surgeries were performed under sodium pentobarbital anesthesia, and all efforts were made to minimize suffering.

**FIGURE 1:**
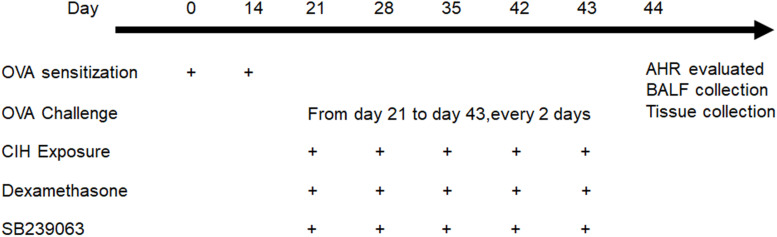
Diagram of animal treatment.

The mice were randomly assigned to six groups, six mice in each group: (1) control; (2) OVA; (3) OVA + dexamethasone (Dex); (4) OVA + CIH; (5) OVA + CIH + Dex; and (6) OVA + CIH + DEX + SB239063; mice in the control group were challenged with aerosolized saline and exposed to room air. In the asthma model, the mice were sensitized intraperitoneally (i.p.) with 20 μg OVA (Sigma-Aldrich, St. Louis, MO, United States) complexed with 2 mg of alum (Shanghai No. 4 Reagent & H.V. Chemical Industries, Ltd., Shanghai, China) in a total volume of 0.1 ml saline on day 0 and day 14. The mice were challenged *via* aerosol nebulization with 5% OVA (w/v) for 30 min every 2 days from day 21 to day 43 (the control mice received saline). On day 21, CIH exposure was initiated. The oxygen content in the CIH exposure chamber was measured throughout several cycles with an oxygen sensor placed on the bottom of the chamber. The animals were exposed to 14–15% O_2_ for 5 s during each 60 s cycle. Each CIH exposure lasted 8 h during the daytime and was repeated on days 21, 28, 35, 42, and 43. The mice were fed with an OVA-free diet. The mice in the OVA-challenged or OVA-challenged combined with the CIH exposure model also received the p38 inhibitor SB239063 and/or Dex. The Dex (2 mg/kg) (Sigma-Aldrich) or Dex + SB239063 (5 mg/kg) (Sigma-Aldrich) was injected i.p. 1 h before CIH exposure (control mice received dimethyl sulfoxide (DMSO) alone). The dose of SB239063 and Dex was adopted based on our previous study (Liang et al., 2013). The protocol is shown in Figure 1.

### Evaluation of Airway Hyperreactivity and Lung Histology

Pulmonary resistance was used to evaluate airway hyperreactivity (AHR) and was detected using an invasive pulmonary device for small animals (FlexiVent, SCIREQ, Montreal, QB, Canada) as reported previously (Aravamudan et al., 2012). The mice were exposed to an aerosol of phosphate-buffered saline (PBS) (baseline readings) followed by cumulatively increased doses of aerosolized methacholine (5, 10, 25, and 50 mg/ml). The aerosol was generated by a portable ultrasonic nebulizer and drawn through the chamber for 3 min with the Bias Flow Supply. The signals were recorded for the subsequent 5 min and the interval between each dose was 1 min. For histology assay, the left pulmonary portion was fixed in a 10% neutral-buffered formalin solution and embedded in paraffin. Lung sections (5 μM) were subjected to hematoxylin and eosin (H&E) staining. The infiltration of inflammatory cells in the peribronchial and perivascular regions was evaluated according to a 0–3 scoring system as described previously (Bao et al., 2013).

### Enzyme-Linked Immunosorbent Assay of Bronchoalveolar Lavage Fluid

In the current study, 24 h after the last CIH exposure, the mice were sacrificed by an overdose of pentobarbital (100 mg/kg i.p.), and the trachea was isolated by blunt dissection. Three successive volumes of 0.3 ml PBS were instilled *via* the endotracheal tube and aspirated gently. The Bronchoalveolar Lavage Fluid (BALF) was pooled from each aspirate. Each BALF sample was centrifuged at 1,000 × *g* for 10 min at 4°C, and the supernatants were stored at −80°C until used. The cell pellets were diluted with 0.5 ml PBS. The total cell counts were determined using a hemocytometer by adding 100 μl of the cell suspension to 100 μl 0.4% trypan blue. The differential cell counts were performed using cytocentrifuge preparations (Cytospin 2; Shandon Instruments, Runcorn, United Kingdom) stained with the Wright-Giemsa stain method.

The levels of interleukin (IL)-4, IL-5, and IL-13 in the BALF were determined using an Enzyme-Linked Immunosorbent Assay (ELISA) Kit (Nanjing Jiancheng Bioengineering Institute, Nanjing, China), according to the standard protocols.

### Biochemical Analysis of Lung Tissue

A sample of lung tissue was fixed in formalin and the remainder was snap-frozen in liquid nitrogen and stored at − 80°C. The levels of malondialdehyde (MDA) and glutathione peroxidase (GSH-Px) in lung tissues and airway smooth muscle cells (ASMCs) were measured using commercial kits in accordance with the protocols from the manufacturer (Nanjing Jiancheng Bioengineering Institute).

### Preparation and Activation of Primary Cultured Mouse ASMCs

In the present study, 24 h after the last aerosol exposure, the normal mice were sacrificed by an overdose of pentobarbital (100 mg/kg i.p.). ASMCs were isolated and identified as described previously (Yin et al., 2014). Briefly, the trachea was placed into a sterile, ice-cold PBS solution. After cutting into small pieces, the segments were digested for 30 min at 37°C in a PBS solution containing 2.0 mg/ml collagenase IV, and then subsequently centrifuged at 200 × *g* for 5 min, and the pellet was resuspended and cultured in DMEM supplemented with 10% FBS. The ASMCs were identified by light microscopy and immunofluorescence. More than 95% of the cells in the primary culture expressed the contractile protein, smooth muscle α-actin. The experiments were performed with cells at passages 3–8. At 90% confluency, the cells were stimulated with transforming growth factor (TGF-β) (10 ng/ml; Proteintech), cultured in the presence or absence of Dex (1 nmol/L; Sigma-Aldrich) or SB239063 (20 μM; Sigma-Aldrich), or both for 48 h. For CIH exposure, the cells were exposed to 14–15% O_2_ for 5 s during each 60 s cycle for 24 h. Each cell experiment was repeated in triplicate.

### Detection of ASMC Proliferation *via* an MTT Assay

The ASMCs were seeded into 96-well plates at a density of 1 × 10^4^ cells/well and treated with different stimulants for the indicatedperiods. Then, 20 μl of 5 mg/ml MTT solution was added to each well to form purple formazan. Subsequently, 150 μl formazans dissolving liquid was added to dissolve the formazan crystals. The optical density (OD) value of each well was detected at a wavelength of 490 nm using a spectrophotometer, and the 50% effective concentration (EC_50_) value was obtained from the MTT viability growth curve. The ASMC proliferation rate was calculated using the following formula: (OD of treated wells/OD of control wells) × 100%.

### Statistical Analysis

The SPSS 21.0 software (SPSS Inc., Chicago, IL, United States) was used to carry out a one-way ANOVA on the results from the various groups with LSD *post hoc* tests, and the measurement data were expressed as means ± SD. Differences were considered statistically significant at *p* < 0.05.

More detailed materials and methods are included in the Supplementary Methods.

## Results

Chronic intermittent hypoxia exposure reduced the effects of Dex in the mouse model, which were ameliorated by the p38 MAPK inhibitor, SB239063.

**FIGURE 2:**
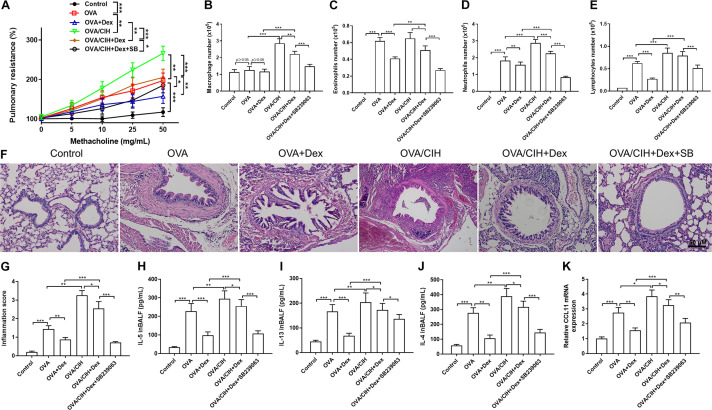
Effects of dexamethasone (Dex) and the p38 MAPK inhibitor, SB239063, on the allergic asthma model with (chronic intermittent hypoxia) CIH exposure. **(A)** Airway hyperreactivity (AHR) was measured using an invasive pulmonary device for mice. **(B)** Macrophage cell count, **(C)** eosinophil cell count, **(D)** neutrophil cell count, and **(E)** lymphocyte cell count in bronchoalveolar lavage fluid (BALF). **(F,G)** Typical hematoxylin and eosin (H&E) staining images of the lung with quantification. **(H)** IL-4, **(I)** IL-5, and **(J)** IL-13 levels in BALF were detected by enzyme-linked immunosorbent assay (ELISA). **(K)** The level of CCL11 mRNA expression in the lungs as determined by Real-Time Quantitative Reverse Transcription-PCR (qRT-PCR). Data are shown as the mean ± SEM (*n* = 6; *p < 0.05; **p < 0.01; ***p < 0.001).

An OVA-induced asthma mouse model was reported to be used to explore the mechanism of hormone resistance asthma (He et al., 2019). We first established an OVA-induced mouse model of asthma with or without CIH exposure. The effects of CIH exposure and SB239063 of OVA-induced AHR were assessed by pulmonary resistance in response to different concentrations of methacholine. Figure 2A shows that pulmonary resistance values were enhanced by methacholine inhalation in the OVA-challenged group and Dex treatment decreased these values. In addition, as compared to control mice, OVA sensitization and challenge provoked a significant increase in the number of macrophages, eosinophils, neutrophils, and lymphocytes in BALF, and these increases were reversed after Dex administration (Figures 2B–E). Based on the results of HE analysis, the OVA challenge caused a significant increase in inflammation scores with peribronchial and perivascular inflammatory cell infiltrates in lung sections compared to control mice. All these changes were attuned to Dex treatment. To investigate the inflammatory effects in the lung, the secretion of IL-4, IL-5, and IL-13 were also detected (Figures 2F,G). The ELISA showed that the levels of IL-4, IL-5, and IL-13 were increased in BALF of the OVA-challenged mice as compared with the controls, and Dex treatment significantly reversed the increases observed in the levels of IL-4, IL-5, and IL-13 (Figures 2H–J). Furthermore, Reverse Transcription-Polymerase Chain Reaction (RT-PCR) results demonstrated that the level of CCL11 mRNA expression was also affected, which indicated that hormone sensitivity was decreased. Dex and SB239063 pretreatment significantly reversed these trends (Figure 2K).

As shown in Figure 2A, CIH exposure further significantly aggravated asthma induced by the OVA challenge. An obvious decrease in the pulmonary resistance value was observed in mice treated with Dex plus SB239063. Furthermore, CIH led to a rise in total cell counts, such as macrophages and neutrophils in BALF: macrophages (*p* < 0.001), neutrophils (*p* < 0.001), and lymphocytes (*p* < 0.001), and enhanced concentrations of IL-5 (*p* = 0.0009), IL-13 (*p* = 0.0128), and IL-4 (*p* < 0.001) (Figures 2B–E). Compared with the OVA + Dex group, the OVA + CIH + Dex group showed higher pulmonary resistance values, more macrophages, lymphocytes, eosinophils, and neutrophils in BALF, and more severe inflammatory scores. These results showed that CIH further aggravated steroid resistance and decreased the efficacy of Dex. However, pretreatment with Dex and SB239063 significantly increased the cell counts: macrophages (*p* < 0.001), eosinophils (*p* < 0.001), neutrophils (*p* < 0.001), and lymphocytes (*p* < 0.001), and enhanced the levels of IL-5 (*p* < 0.001), IL-13 (*p* = 0.049), and IL-4 (*p* < 0.001). H&E staining revealed that the OVA challenge plus CIH exposure mice exhibited more severe inflammatory scores compared to mice in the control group. However, pretreatment with Dex significantly reduced the inflammation scores in OVA-induced asthma and CIH-treated mice (Figures 2F,G). Furthermore, treatment with the p38 MAPK inhibitor (SB239063) substantially decreased the inflammation scores. The ELISA indicated that there was a steep increase in the production of IL-4, IL-5, and IL-13 in the BALF of the OVA challenged plus CIH mice vs. the OVA challenged mice (Figures 2H–J). The RT-PCR results confirmed that the mRNA level of CCL11 was also enhanced in the CIH+OVA group compared with OVA-challenged mice. Dex and SB239063 pretreatment significantly reversed these trends (Figure 2K).

**FIGURE 3:**
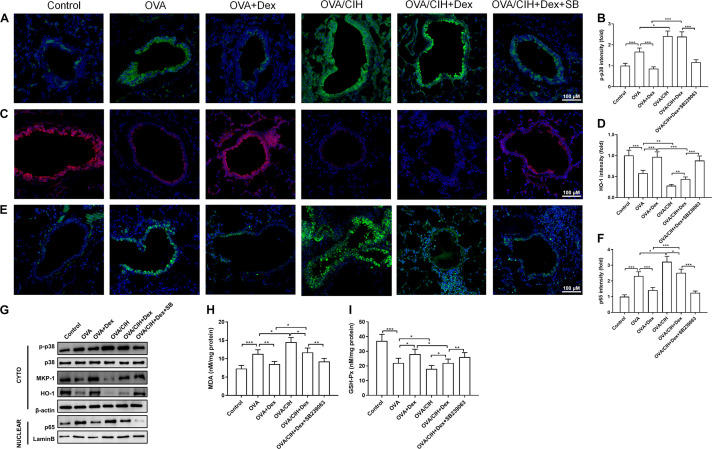
The p38 MAPK pathway regulated allergic asthma in mice with CIH exposure. **(A,B)** Representative images of phosphor-p38 (green) and DAPI (blue) immunofluorescence-stained lung sections and phosphor-p38 intensity. **(C,D)** Representative images of HO-1 (red) and DAPI (blue) immunofluorescence-stained lung sections and HO-1 intensity. **(E,F)** Representative images of p65 (green) and DAPI (blue) immunofluorescence-stained lung sections and p65 intensity. **(G)** Levels of phosphor-p38, MKP-1, HO-1, and nuclear p65 in the lung were detected by western blot. **(H)** Lung MDA concentration. **(I)** Lung GSH-Px activity. Data are shown as the means ± SEM (*n* = 6; *p < 0.05; **p < 0.01; ***p < 0.001).

Extensive research has shown that p65 and HO-1 modulated by p38 MAPK were involved in the progression of steroid resistance (Zhang et al., 2013; Panda et al., 2017; Cho et al., 2018; Lin et al., 2019). To investigate the p38 MAPK signaling pathway in our mouse model of asthma, immunofluorescence and western blots were carried out to analyze the expression of p-p38, HO-1, and p65 in lung sections. The immunofluorescence staining results showed that OVA increased the level of phosphorylated p38 MAPK in the cytoplasm and inflammation response marker p65 in the nucleus while inhibiting the expression of oxidative stress marker HO-1 (Figures 3A–F). In addition, western blot results showed that OVA-induced asthma increased the expression level of p-p38 in the cytoplasm and p65 in the nucleus and decreased the levels of MKP-1 and HO-1 in the cytoplasm (Figure 3G). Furthermore, the indicators of an oxidative stress injury in lung tissue were also detected using commercial kits. Compared to the control group, the OVA challenge provoked a significant increase in MDA and a decrease in GSH-Px (Figures 3H,I).

The immunofluorescence staining results revealed that OVA challenge plus CIH exposure caused a further significant increase in the levels of p38 MAPK in the cytoplasm and p65 in the nucleus and suppressed the level of HO-1 in the cytoplasm, which was not significantly reversed when treated with Dex. However, Dex combined with SB239063 inhibited the levels of phosphorylated p38 MAPK in the cytoplasm and p65 in the nucleus and promoted the expression of HO-1 in the cytoplasm (Figures 3A–F). In addition, western blot results showed that OVA and CIH-induced asthma increased the expression level of p-p38 in the cytoplasm and p65 in the nucleus, and decreased the levels of MKP-1 and HO-1 in the cytoplasm. These findings indicated that CIH affected asthma hormone sensitivity by activating the p38 signaling pathway. Dex and SB239063 pretreatment significantly reduced p-p38 in the cytoplasm and p65 in the nucleus, and the levels of MKP-1 and HO-1 were increased in the cytoplasm (Figure 3G). CIH-induced asthma further significantly enhanced MDA concentration and suppressed GSH-Px in pulmonary tissue, while Dex and SB239063 treatment significantly reduced MDA and increased GSH-Px (Figures 3H,I).

### Chronic Intermittent Hypoxia Exposure Reduced the Effects of Dex in ASMCs, Which Was Reversed by the p38 MAPK Inhibitor

**FIGURE 4:**
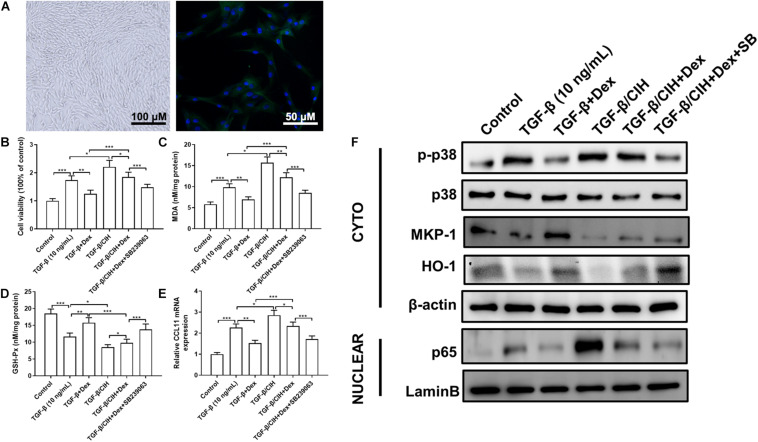
Effects of Dex or the p38 MAPK inhibitor, SB239063, on airway smooth muscle cells (ASMCs). **(A)** ASMCs displayed the characteristic “hill and valley” appearance. Immunofluorescence staining indicated the expression of the contractile protein SM α-actin (Green). **(B)** Proliferation of ASMCs at 48 h was analyzed in the different groups. Cell proliferation following TGF-β (10 ng/ml) stimulation was determined by the MMT test. **(C)** The malondialdehyde (MDA) concentration in ASMCs was measured. **(D)** Activity of GSH-Px in ASMCs was measured. **(E)** The level of CCL11 mRNA expression in ASMCs was determined by qRT-PCR. **(F)** The levels of phosphor-p38, MKP-1, HO-1, and nuclear p65 protein in ASMCs were determined by western blotting. Data are shown as the mean ± SEM (*n* = 3; *p < 0.05; **p < 0.01; ***p < 0.001).

As shown in Figure 4A, primary ASMCs demonstrated a typical characteristic of smooth muscle cells in culture, such as spindle morphology and a hill-and-valley-like pattern. Further, immunofluorescence staining detected that the cells at confluence expressed smooth muscle-specific actin as well as SM α-actin. We treated primary ASMCs with TGF-β, Dex or SB239063, and cultured them under normal or CIH conditions. To detect ASMC proliferation, an MTT assay was performed for each group. As shown in Figure 4B, the level of ASMC proliferation was increased in the TGF-β group, and treatment with Dex suppressed this proliferation. In addition, the MDA concentration and GSH-Px activity were detected in each group (Figures 4C,D). It was found that the Dex group exhibited a lower MDA concentration and higher GSH-Px activity than the TGF-β group. To investigate the inflammatory effects at the cellular level, the level of CCL11 mRNA was analyzed in ASMCs. As shown in Figure 4E, the level of CCL11 was significantly higher in the TGF-β group as compared with the control group. It can be seen in Figure 4F, that p-p38 is upregulated in the TGF-β-treated ASMCs compared with the control group. After Dex treatment, the level of p-p38 phosphorylation was significantly decreased in the TGF-β group. Compared with the control group, HO-1 and MKP-1 expressions were decreased in the TGF-β group.

We, then, investigated the effects of CIH exposure on ASMCs. The MTT assay revealed that proliferation was significantly increased in the CIH group compared with the TGF-β group. However, treatment with Dex alone did not show marked changes vs. the TGF-β group, and the combination of Dex and SB239063 suppressed this proliferative effect (Figure 4B). The combined application of Dex and SB239063 also reduced the level of MDA and increased the level of GSH-Px, which was aggravated by CIH exposure (Figures 4C,D). As shown in Figure 4E, CIH exposure caused further significant increases in these levels. However, in the groups that received combined treatment with SB239063 and Dex, the levels of CCL11 were significantly decreased, which were similar to the TGF-β group treated with Dex alone. Western blotting was performed to further assess whether CIH affected steroid sensitivity by activating the p38 MAPK signaling pathway *in vitro*. Dex alone did not attenuate p38 phosphorylation in the TGF-β/CIH group. However, Dex in combination with SB239063 significantly decreased the level of p-p38 protein expression in the TGF-β/CIH group (Figure 4F).

## Discussion

Most patients with asthma respond well to therapy with GCs. However, some patients have reduced sensitivity to GCs, which means that long-term or high-dose GCs are needed. It has been reported that OSA was a risk factor for refractory asthma, attributed to the effect of CIH on GC insensitivity (Prasad et al., 2020). In this study, we found that CIH exposure significantly increased airway responsiveness in mice. It is noteworthy that treatment with continuous positive airway pressure (CPAP) could not ameliorate FEV1 and AHR in asthma patients with OSA in a recent systematic review (Davies et al., 2018). In contrast, our results demonstrated that combining Dex with SB239063 promoted the depression of airway resistance. The airway structure of clinically treated OSAHS patients is already altered and short-term CPAP is unable to change the airway smooth muscle structure and may require longer CPAP treatment. In this article, Dex and SB239063 interventions have been initiated at the same time as CIH exposure and had a preventive effect. The OVA challenge combined with CIH exposure further led to the production of cytokines. The results suggested that CIH led to a decrease in Dex sensitivity. These results supported previous research by Martin et al. (Leonard et al., 2005), who suggested that hypoxia resulted in attenuation of enhanced GC sensitivity. For many years, asthma was thought to be primarily mediated by an adaptive immune response. The main cells involved in T helper type 2 (TH2) cells-high asthma are eosinophils and basophils, mast cells, TH2, ILC2s, and IgE-producing B cells. TH2-low asthma does not involve eosinophils and has a poor response to GCs and type II immunosuppressive agents (Fahy, 2015). As shown in Figure 2C, there was no significant change in eosinophils. We speculate that asthma combined with CIH exposure may be associated with TH2-low asthma and steroid resistance.

Inflammation is the key factor in asthma (Keane-Myers et al., 1998; Jember et al., 2001; Al-Shami et al., 2005; Lee et al., 2011; Lin et al., 2016). The reports have shown that intermittent hypoxia can induce airway inflammation in rats (Broytman et al., 2015). In addition, inhibition of p38 has an anti-inflammatory effect and is effective in asthma models by reversing insensitivity to GCs (Liang et al., 2013). In this study, the data confirmed that SB239063, a p38 MAPK inhibitor, inhibited the SR inflammation response both *in vitro* and *in vivo*. Previous research found that p38 MAPK activity was selectively increased in the peripheral blood mononuclear cells of patients with SR asthma, and activation of the p38 MAPK pathway in blood could be used as a selective biomarker in patients with SR asthma (Li et al., 2015). Furthermore, *in vitro* experiments showed that the combined use of p38 MAPK inhibitors and GCs could significantly inhibit the levels of cytokines in patients with asthma, while p38 inhibitors could improve and reverse hormone insensitivity, as well as increase the anti-inflammatory effects of hormones (Bhavsar et al., 2010; Lea et al., 2015). Furthermore, p38 MAPK inhibitors also significantly inhibited the mRNA of monocyte-macrophage inflammatory genes in patients with chronic obstructive pulmonary disease (Kent et al., 2009). Thus, it was hypothesized that the p38 MAPK signaling pathway may be involved in CIH-induced SR asthma. We found that CIH exposure increased the activity of p38 MAPK in lung tissue and ASMCs, which was effectively inhibited by SB239063, a p38 MAPK inhibitor. In addition, SB239063 suppressed ASMCs proliferation, oxidative stress activation, and inflammatory cell recruitment both *in vitro* and *in vivo*. These findings are consistent with previous observations by several research groups that p38 MAPK inhibition in severe asthma may enhance cellular responsiveness to GCs, and we first found that p38 MAPK inhibition ameliorated CIH-induced SR asthma both *in vitro* and *in vivo*.

The activation of p38 MAPK regulates various inflammatory signaling pathways (O’Dea et al., 2011). Our results showed that MKP-1 was found to have significant interactions with anti-inflammatory proteins mediated by p38 MAPK in the development of asthma (Prabhala et al., 2015). MKP-1 plays a crucial negative feedback role in MAPK signaling and regulates the pro-inflammatory response and cytokine secretion from ASMCs caused by MAPK pathway stimulation (Bao et al., 2014). In addition, several studies found that MKP-1 may inhibit the inflammatory response by inhibiting p65 nuclear transcription (Chen et al., 2015; Kovacs et al., 2019), and the level of p38 MAPK activity in severe asthma patients increases in alveolar macrophages, and the level of MKP-1 mRNA expression in hormone-induced cells is reduced (Bhavsar et al., 2008). CIH leads to the inhibition of MKP-1 function and an aggravated inflammatory response, which can cause impaired GC receptor function and hormone resistance. SB239063 can increase the expression level of MKP-1, prevent the translocation of p65 into the nucleus, and restore hormone sensitivity. Our results confirmed the above theory. It was also demonstrated in the present study that SB239063 inhibited the level of phosphorylated p38 MAPK and promoted the expression of HO-1 both *in vivo* and *in vitro*. HO-1 and its by-products, biliverdin are considered to have cytoprotective properties, such as anti-oxidant, anti-inflammatory, and anti-apoptotic activities in the context of lung disease (Ahmed et al., 2019), and are regulated by p38 MAPK (Cho et al., 2018; Nakashima et al., 2018). CIH can induce oxidative stress in asthma, stimulate the p38 pathway, cause GR dysfunction, and reduce the expression levels of histone deacetylase (HDAC2) (Park et al., 2007; Klusonova et al., 2009; Du et al., 2015). Therefore, targeted overexpression of HO-1 may be helpful in the treatment of CIH-induced lung oxidative stress disorders.

Steroid-resistant asthma is a complicated pathophysiological process in which various types of asthma cells display increased p38 MAPK signaling, inflammatory response, and oxidative stress activation. We found that p38 MAPK activation participated in OVA- and hypoxia-induced asthma in mice. This led to a decline in GC sensitivity, inducing the release of inflammatory mediators and airway remodeling, thus leading to uncontrolled and more severe asthma. It can be concluded that p38 MAPK inhibitors improved the sensitivity of GCs in mice with CIH and asthma by inhibiting p38 MAPK expression.

We speculate that p38 inhibitor combined with CPAP treatment will enable patients with asthma and OSA to achieve a better level of control, probably because it increases hormone sensitivity by improving CIH in these patients.

## Conclusion

Chronic intermittent hypoxia exposure aggravated asthma and reduced GC sensitivity by stimulating the p38 MAPK signaling pathway. Our data revealed that the p38 MAPK pathway may be a promising potential target for SR asthma combined with OSA.

## Clinical Perspectives

(1) Obstructive sleep apnea is a risk factor for refractory asthma. Patients with asthma and OSA are more likely to develop steroid resistance. Activation of the p38 MAPK pathway has been reported to participate in SR asthma. Our study aimed to examine how the p38 MAPK pathway affected CIH exposure combined with OVA-induced asthma in a mouse model.
(2) The results showed that the p38 MAPK inhibitor SB239063 ameliorated CIH combined with OVA-induced asthma by increasing GC sensitivity and reducing oxidative stress injury.
(3) These findings provided a novel insight into SR asthma combined with OSA.

## Data Availability Statement

The raw data supporting the conclusions of this article will be made available by the authors, without undue reservation.

## Ethics Statement

The protocol was reviewed and approved by the Shanghai Ninth People’s Hospital Institutional Review Board (Permit Number: HKDL2017265) and all animal experiments were conducted in Shanghai Ninth People’s Hospital.

## Author Contributions

LL designed and performed the experiments. LL, XG, YS, YL, YC, and ZC performed the experiments and wrote the manuscript. HS, YL, and JZ analyzed the data. JM and QL conceived the experiments. All authors have read and approved the final manuscript.

## Conflict of Interest

The authors declare that the research was conducted in the absence of any commercial or financial relationships that could be construed as a potential conflict of interest.

## Publisher’s Note

All claims expressed in this article are solely those of the authors and do not necessarily represent those of their affiliated organizations, or those of the publisher, the editors and the reviewers. Any product that may be evaluated in this article, or claim that may be made by its manufacturer, is not guaranteed or endorsed by the publisher.

## References

[B1] Ahmed R. F. Moussa R. A. Eldemerdash R. S. Zakaria M. M. Abdel-Gaber S. A. (2019). Ameliorative effects of silymarin on HCl-induced acute lung injury in rats; role of the Nrf-2/HO-1 pathway. *Iran J. Basic Med. Sci.* 22 1483–1492. 10.22038/IJBMS.2019.14069 32133068 PMC7043873

[B2] Al-Shami A. Spolski R. Kelly J. Keane-Myers A. Leonard W. J. (2005). A role for TSLP in the development of inflammation in an asthma model. *J. Exp. Med.* 202 829–839. 10.1084/jem.20050199 16172260 PMC2212950

[B3] Aravamudan B. VanOosten S. K. Meuchel L. W. Vohra P. Thompson M. Sieck G. C. (2012). Caveolin-1 knockout mice exhibit airway hyperreactivity. *Am. J. Physiol. Lung. Cell. Mol. Physiol.* 303 L669–L681. 10.1152/ajplung.00018.2012 22923642 PMC3469637

[B4] Bao A. Li F. Zhang M. Chen Y. Zhang P. Zhou X. (2014). Impact of ozone exposure on the response to glucocorticoid in a mouse model of asthma: involvements of p38 MAPK and MKP-1. *Respir Res.* 15:126. 10.1186/s12931-014-0126-x 25287866 PMC4196074

[B5] Bao A. Liang L. Li F. Zhang M. Zhou X. (2013). Effects of acute ozone exposure on lung peak allergic inflammation of mice. *Front. Biosci.* 18:838–851. 10.2741/4147 23747851

[B6] Bhavsar P. Hew M. Khorasani N. Torrego A. Barnes P. J. Adcock I. (2008). Relative corticosteroid insensitivity of alveolar macrophages in severe asthma compared with non-severe asthma. *Thorax* 63 784–790. 10.1136/thx.2007.090027 18492738

[B7] Bhavsar P. Khorasani N. Hew M. Johnson M. Chung K. F. (2010). Effect of p38 MAPK inhibition on corticosteroid suppression of cytokine release in severe asthma. *Eur. Respir J.* 35 750–756. 10.1183/09031936.00071309 19840967

[B8] Boonpiyathad T. Sozener Z. C. Satitsuksanoa P. Akdis C. A. (2019). Immunologic mechanisms in asthma. *Semin. Immunol.* 46:101333. 10.1016/j.smim.2019.101333 31703832

[B9] Broytman O. Braun R. K. Morgan B. J. Pegelow D. F. Hsu P. N. Mei L. S. (2015). Effects of chronic intermittent hypoxia on allergen-induced airway inflammation in rats. *Am. J. Respir Cell. Mol. Biol.* 52 162–170. 10.1165/rcmb.2014-0213OC 25004109

[B10] Chen W. C. Yen C. S. Huang W. J. Hsu Y. F. Ou G. Hsu M. J. (2015). WMJ-S-001, a novel aliphatic hydroxamate derivative, exhibits anti-inflammatory properties via MKP-1 in LPS-stimulated RAW264.7 macrophages. *Br. J. Pharmacol.* 172 1894–1908. 10.1111/bph.13040 25521622 PMC4376465

[B11] Cho R. L. Lin W. N. Wang C. Y. Yang C. C. Hsiao L. D. Lin C. C. (2018). Heme oxygenase-1 induction by rosiglitazone via PKCalpha/AMPKalpha/p38 MAPKalpha/SIRT1/PPARgamma pathway suppresses lipopolysaccharide-mediated pulmonary inflammation. *Biochem. Pharmacol.* 148 222–237. 10.1016/j.bcp.2017.12.024 29309760

[B12] Davies S. E. Bishopp A. Wharton S. Turner A. M. Mansur A. H. (2018). Does continuous positive airway pressure (CPAP) treatment of obstructive sleep apnoea (OSA) improve asthma-related clinical outcomes in patients with co-existing conditions? A systematic review. *Respir Med.* 143 18–30. 10.1016/j.rmed.2018.08.004 30261988

[B13] Du J. Zhang L. Zhuang S. Qin G. J. Zhao T. C. (2015). HDAC4 degradation mediates HDAC inhibition-induced protective effects against hypoxia/reoxygenation injury. *J. Cell Physiol.* 230 1321–1331. 10.1002/jcp.24871 25475100 PMC4373665

[B14] Fahy J. V. (2015). Type 2 inflammation in asthmapresent in most, absent in many. *Nat. Rev. Immunol.* 15 57–65. 10.1038/nri3786 25534623 PMC4390063

[B15] Guo L. Yang Y. An B. Yang Y. Shi L. Han X. (2017). Risk factors for dislocation after revision total hip arthroplasty: a systematic review and meta-analysis. *Int. J. Surg.* 38 123–129. 10.1016/j.ijsu.2016.12.122 28043927

[B16] He Y. Shi J. Nguyen Q. T. You E. Liu H. Ren X. (2019). Development of highly potent glucocorticoids for steroid-resistant severe asthma. *Proc. Natl. Acad. Sci. U.S.A.* 116 6932–6937. 10.1073/pnas.1816734116 30894497 PMC6452690

[B17] Jember A. G. Zuberi R. Liu F. T. Croft M. (2001). Development of allergic inflammation in a murine model of asthma is dependent on the costimulatory receptor OX40. *J. Exp. Med.* 193 387–392. 10.1084/jem.193.3.387 11157058 PMC2195923

[B18] Julien J. Y. Martin J. G. Ernst P. Olivenstein R. Hamid Q. Lemiere C. (2009). Prevalence of obstructive sleep apnea-hypopnea in severe versus moderate asthma. *J. Allergy Clin. Immunol.* 124 371–376. 10.1016/j.jaci.2009.05.016 19560194

[B19] Keane-Myers A. M. Gause W. C. Finkelman F. D. Xhou X. D. Wills-Karp M. (1998). Development of murine allergic asthma is dependent upon B7-2 costimulation. *J. Immunol.* 160 1036–1043. 9551945

[B20] Kent L. M. Smyth L. J. Plumb J. Clayton C. L. Fox S. M. Ray D. W. (2009). Inhibition of lipopolysaccharide-stimulated chronic obstructive pulmonary disease macrophage inflammatory gene expression by dexamethasone and the p38 mitogen-activated protein kinase inhibitor N-cyano-N’-(2-{[8-(2,6-difluorophenyl)-4-(4-fluoro-2-methylphenyl)-7-oxo-7,8-dihy dropyrido[2,3-d] pyrimidin-2-yl]amino}ethyl)guanidine (SB706504). *J. Pharmacol. Exp. Ther.* 328 458–468. 10.1124/jpet.108.142950 19004925 PMC2682286

[B21] Klusonova P. Rehakova L. Borchert G. Vagnerova K. Neckar J. Ergang P. (2009). Chronic intermittent hypoxia induces 11beta-hydroxysteroid dehydrogenase in rat heart. *Endocrinology* 150 4270–4277. 10.1210/en.2008-1493 19470702

[B22] Kovacs K. Vaczy A. Fekete K. Kovari P. Atlasz T. Reglodi D. (2019). PARP inhibitor protects against chronic hypoxia/reoxygenation-induced retinal injury by regulation of MAPKs, HIF1alpha, Nrf2, and NFkappaB. *Invest. Ophthalmol. Vis. Sci.* 60 1478–1490. 10.1167/iovs.18-25936 30973576

[B23] Lambrecht B. N. Hammad H. Fahy J. V. (2019). The cytokines of asthma. *Immunity* 50 975–991. 10.1016/j.immuni.2019.03.018 30995510

[B24] Lea S. Harbron C. Khan N. Booth G. Armstrong J. Singh D. (2015). Corticosteroid insensitive alveolar macrophages from asthma patients; synergistic interaction with a p38 mitogen-activated protein kinase (MAPK) inhibitor. *Br. J. Clin. Pharmacol.* 79 756–766. 10.1111/bcp.12536 25358442 PMC4415712

[B25] Lee K. Kim S. H. Yoon H. J. Paik D. J. Kim J. M. Youn J. (2011). Bacillus-derived poly-gamma-glutamic acid attenuates allergic airway inflammation through a Toll-like receptor-4-dependent pathway in a murine model of asthma. *Clin. Exp. Allergy* 41 1143–1156. 10.1111/j.1365-2222.2011.03792.x 21672055

[B26] Lee M. Y. Wang Y. Mak J. C. Ip M. S. (2016). Intermittent hypoxia induces NF-kappaB-dependent endothelial activation via adipocyte-derived mediators. *Am. J. Physiol. Cell. Physiol.* 310 C446–C455. 10.1152/ajpcell.00240.2015 26739492

[B27] Leonard M. O. Godson C. Brady H. R. Taylor C. T. (2005). Potentiation of glucocorticoid activity in hypoxia through induction of the glucocorticoid receptor. *J. Immunol.* 174 2250–2257. 10.4049/jimmunol.174.4.2250 15699159

[B28] Li L. B. Leung D. Y. Goleva E. (2015). Activated p38 MAPK in peripheral blood monocytes of steroid resistant asthmatics. *PLoS One* 10:e0141909. 10.1371/journal.pone.0141909 26517722 PMC4627650

[B29] Liang L. Li F. Bao A. Zhang M. Chung K. F. Zhou X. (2013). Activation of p38 mitogen-activated protein kinase in ovalbumin and ozone-induced mouse model of asthma. *Respirology* 18 (Suppl. 3) 20–29. 10.1111/resp.12189 24188200

[B30] Lin C. L. Hsiao G. Wang C. C. Lee Y. L. (2016). Imperatorin exerts antiallergic effects in Th2-mediated allergic asthma via induction of IL-10-producing regulatory T cells by modulating the function of dendritic cells. *Pharmacol. Res.* 110 111–121. 10.1016/j.phrs.2016.04.030 27185659

[B31] Lin L. P. Niu G. H. Zhang X. Q. (2019). Influence of lncRNA MALAT1 on septic lung injury in mice through p38 MAPK/p65 NF-kappaB pathway. *Eur. Rev. Med. Pharmacol. Sci.* 23 1296–1304. 10.26355/eurrev_201902_17025 30779099

[B32] Liu S. Sun J. Y. Ren L. P. Chen K. Xu B. (2017). Propofol attenuates intermittent hypoxia induced up-regulation of proinflammatory cytokines in microglia through inhibiting the activation of NF-Bkappa/p38 MAPK signalling. *Folia Neuropathol.* 55 124–131. 10.5114/fn.2017.68579 28677369

[B33] McGregor M. C. Krings J. G. Nair P. Castro M. (2019). Role of biologics in asthma. *Am. J. Respir. Crit. Care Med.* 199 433–445. 10.1164/rccm.201810-1944CI 30525902 PMC6835092

[B34] McManus R. (2003). Mechanisms of steroid action and resistance in inflammation and disease. *J. Endocrinol.* 178 1–4. 10.1677/joe.0.1780001 12844329

[B35] Nakashima K. Sato T. Shigemori S. Shimosato T. Shinkai M. Kaneko T. (2018). Regulatory role of heme oxygenase-1 in silica-induced lung injury. *Respir Res.* 19:144. 10.1186/s12931-018-0852-6 30068325 PMC6090697

[B36] O’Dea K. P. Dokpesi J. O. Tatham K. C. Wilson M. R. Takata M. (2011). Regulation of monocyte subset proinflammatory responses within the lung microvasculature by the p38 MAPK/MK2 pathway. *Am. J. Physiol. Lung. Cell Mol. Physiol.* 301 L812–L821. 10.1152/ajplung.00092.2011 21873449 PMC3213987

[B37] Panda L. Gheware A. Rehman R. Yadav M. K. Jayaraj B. S. Madhunapantula S. V. (2017). Linoleic acid metabolite leads to steroid resistant asthma features partially through NF-kappaB. *Sci. Rep.* 7:9565. 10.1038/s41598-017-09869-9 28851976 PMC5575291

[B38] Park A. M. Nagase H. Kumar S. V. Suzuki Y. J. (2007). Effects of intermittent hypoxia on the heart. *Antioxid Redox Signal* 9 723–729. 10.1089/ars.2007.1460 17511587

[B39] Prabhala P. Bunge K. Rahman M. M. Ge Q. Clark A. R. Ammit A. J. (2015). Temporal regulation of cytokine mRNA expression by tristetraprolin: dynamic control by p38 MAPK and MKP-1. *Am. J. Physiol. Lung Cell Mol. Physiol.* 308 L973–L980. 10.1152/ajplung.00219.2014 25724669

[B40] Prasad B. Nyenhuis S. M. Imayama I. Siddiqi A. Teodorescu M. (2020). Asthma and obstructive sleep apnea overlap: what has the evidence taught us? *Am. J. Respir Crit. Care Med.* 201 1345–1357. 10.1164/rccm.201810-1838TR 31841642 PMC7258643

[B41] Qiao Y. X. Xiao Y. (2015). Asthma and obstructive sleep apnea. *Chin. Med. J.* 128 2798–2804. 10.4103/0366-6999.167361 26481749 PMC4736887

[B42] Ramamoorthy S. Cidlowski J. A. (2013). Exploring the molecular mechanisms of glucocorticoid receptor action from sensitivity to resistance. *Endocr Dev.* 24 41–56. 10.1159/000342502 23392094 PMC4770453

[B43] Somers V. K. Dyken M. E. Clary M. P. Abboud F. M. (1995). Sympathetic neural mechanisms in obstructive sleep apnea. *J. Clin. Invest.* 96 1897–1904. 10.1172/JCI118235 7560081 PMC185826

[B44] ten Brinke A. Sterk P. J. Masclee A. A. Spinhoven P. Schmidt J. T. Zwinderman A. H. (2005). Risk factors of frequent exacerbations in difficult-to-treat asthma. *Eur. Respir J.* 26 812–818. 10.1183/09031936.05.00037905 16264041

[B45] Tsai S. C. (2017). Chronic obstructive pulmonary disease and sleep related disorders. *Curr. Opin. Pulm. Med.* 23 124–128. 10.1097/MCP.0000000000000351 27984243

[B46] Yin L. M. Wei Y. Wang W. Q. Wang Y. Xu Y. D. Yang Y. Q. (2014). Simultaneous application of BrdU and WST-1 measurements for detection of the proliferation and viability of airway smooth muscle cells. *Biol. Res.* 47:75. 10.1186/0717-6287-47-75 25723317 PMC4289569

[B47] Zhang Y. Zhang L. Wu J. Di C. Xia Z. (2013). Heme oxygenase-1 exerts a protective role in ovalbumin-induced neutrophilic airway inflammation by inhibiting Th17 cell-mediated immune response. *J. Biol. Chem.* 288 34612–34626. 10.1074/jbc.M113.494369 24097973 PMC3843074

