# Supplementary figures and images for: Chronic Intermittent Hypoxia Reduces the Effects of Glucosteroid in Asthma via Activating the p38 MAPK Signaling Pathway
Source: Front Physiol. 2021 Aug 27;12:703281. doi: 10.3389/fphys.2021.703281 (PMC8430218; doi:10.3389/fphys.2021.703281)

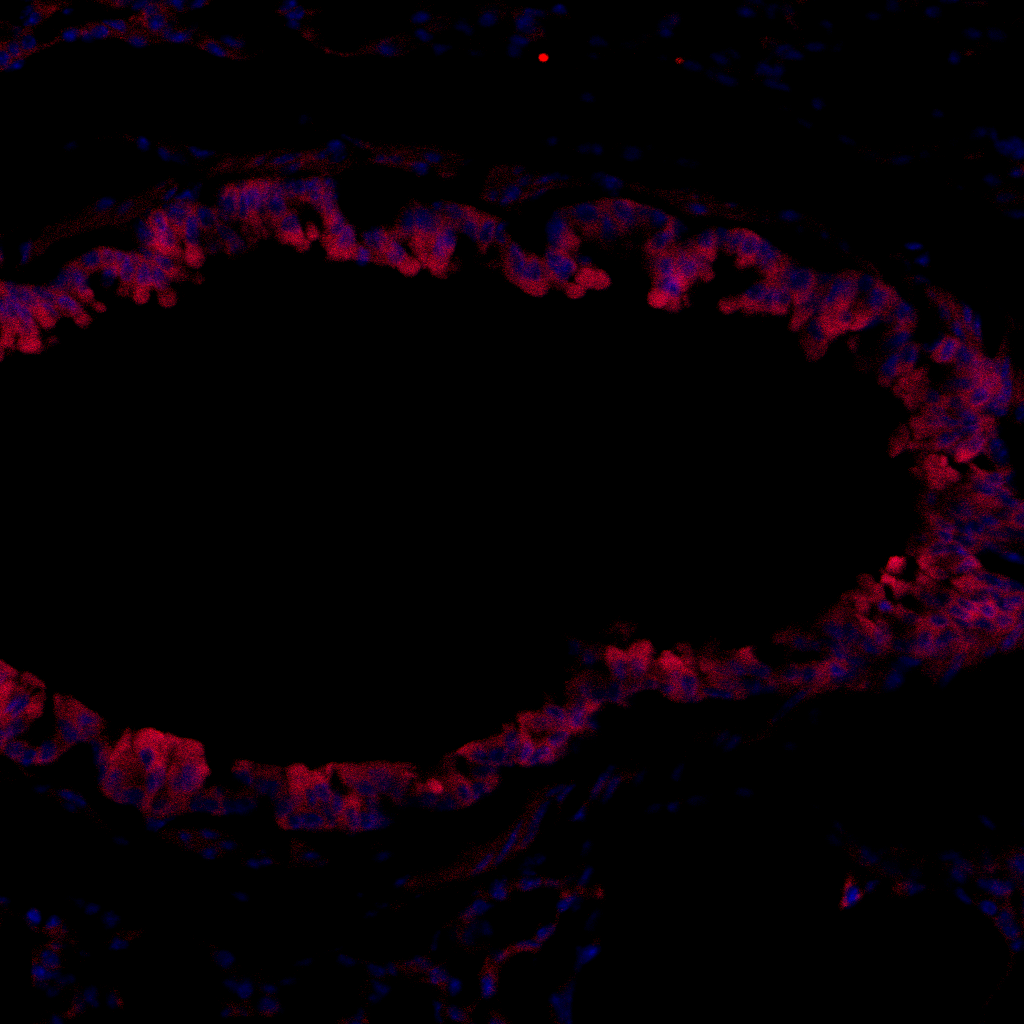

Supplement: Supplementary file 1 [file Data_Sheet_1.ZIP › Immunofluorescence/HO-1/control/1.tif]

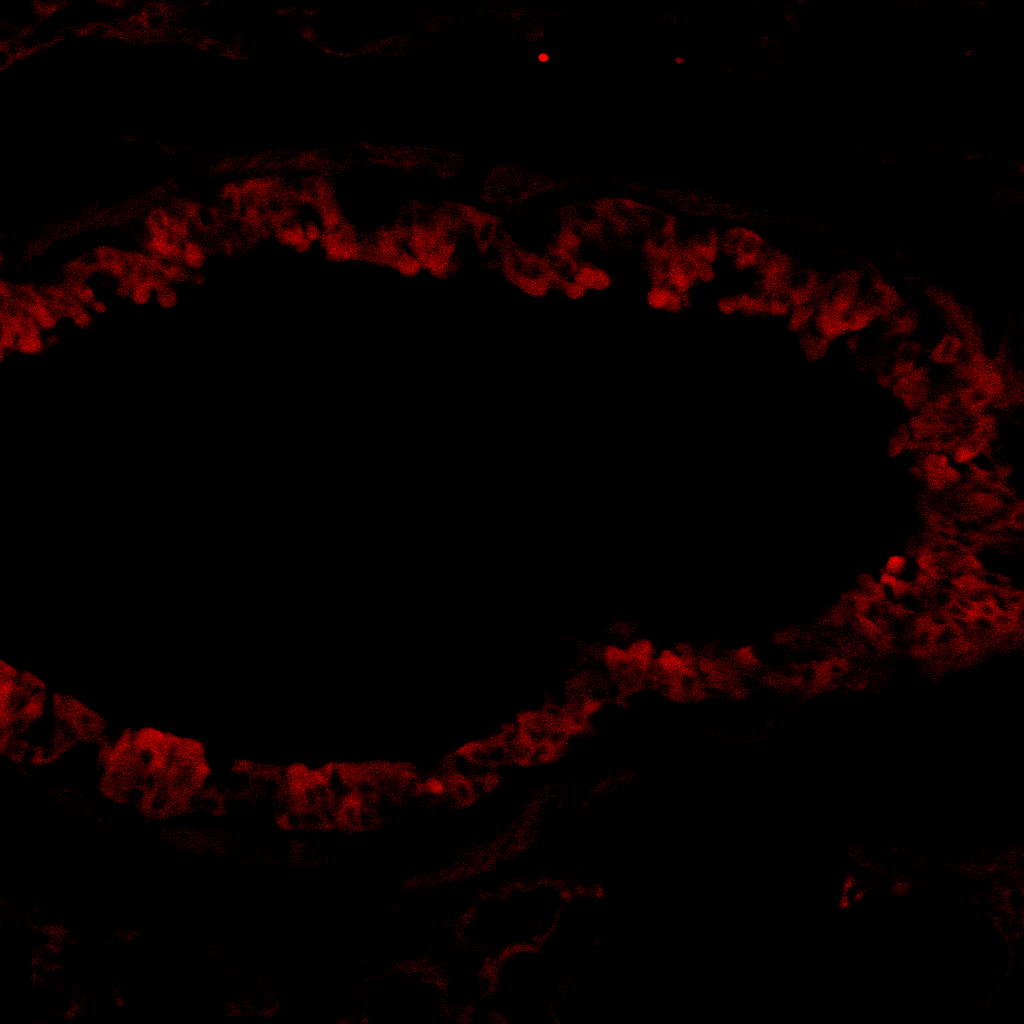

Supplement: Supplementary file 1 [file Data_Sheet_1.ZIP › Immunofluorescence/HO-1/control/2.tif]

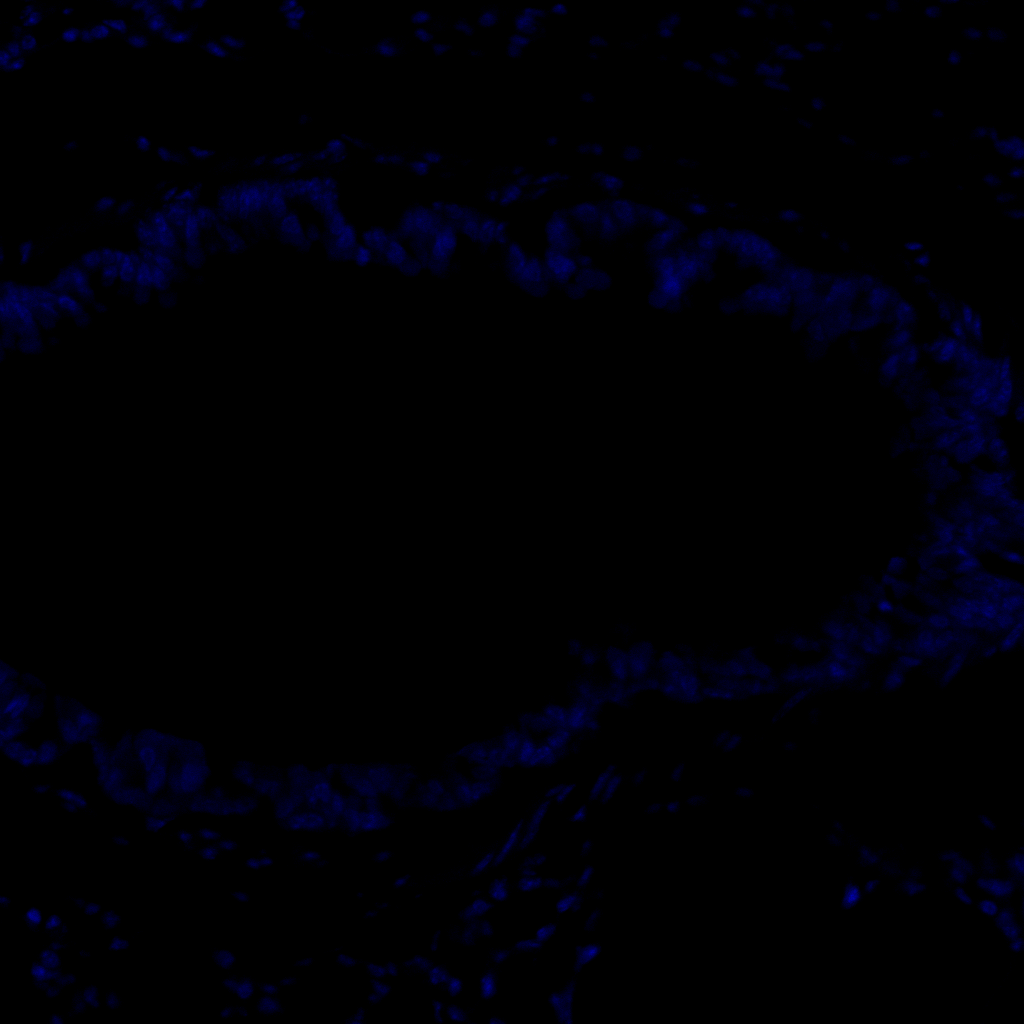

Supplement: Supplementary file 1 [file Data_Sheet_1.ZIP › Immunofluorescence/HO-1/control/3.tif]

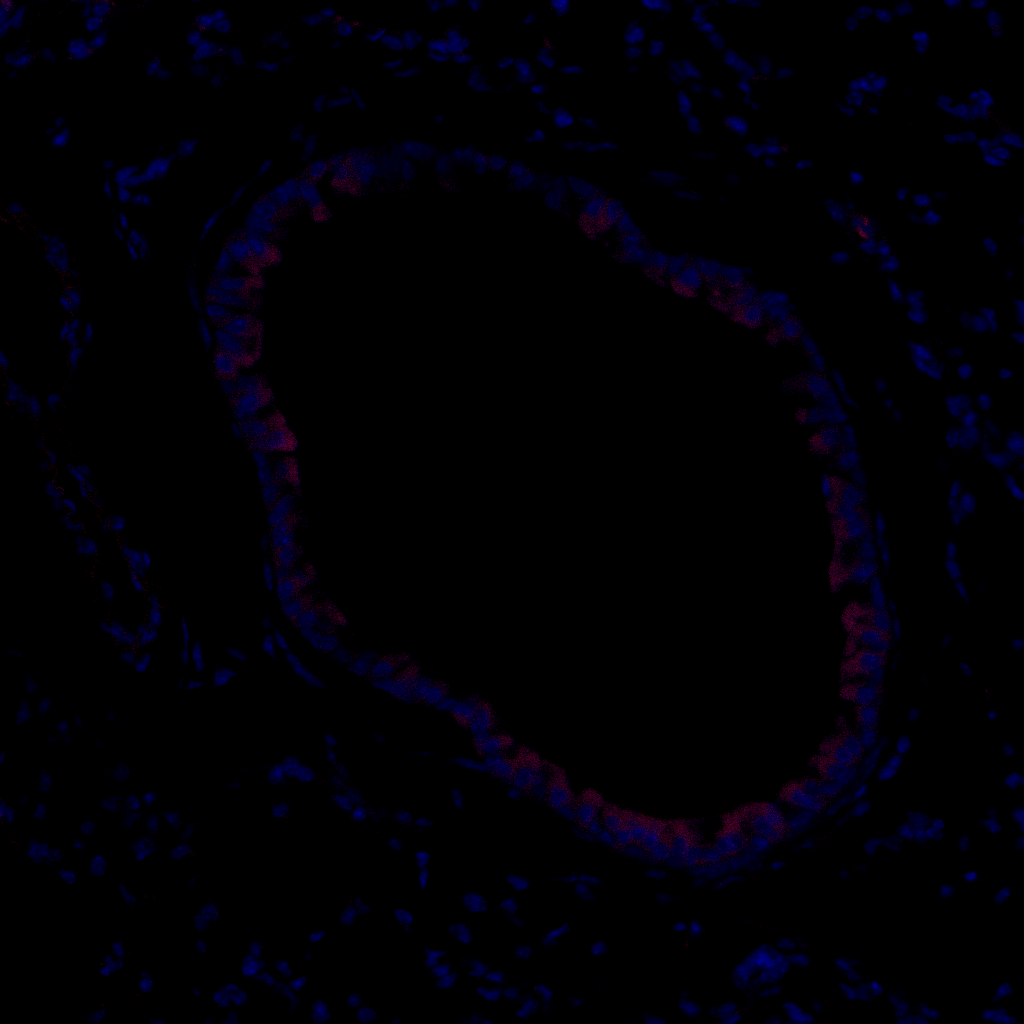

Supplement: Supplementary file 1 [file Data_Sheet_1.ZIP › Immunofluorescence/HO-1/OVA/1.tif]

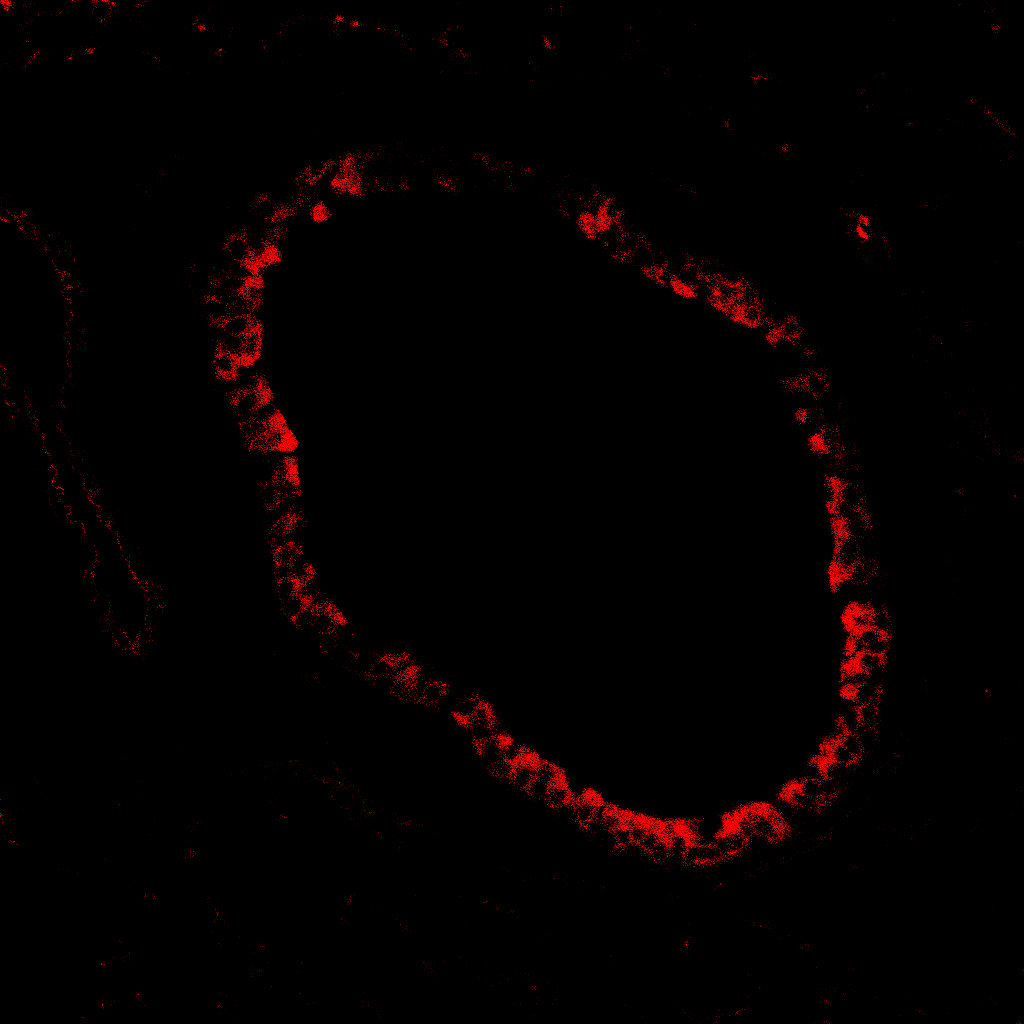

Supplement: Supplementary file 1 [file Data_Sheet_1.ZIP › Immunofluorescence/HO-1/OVA/2.tif]

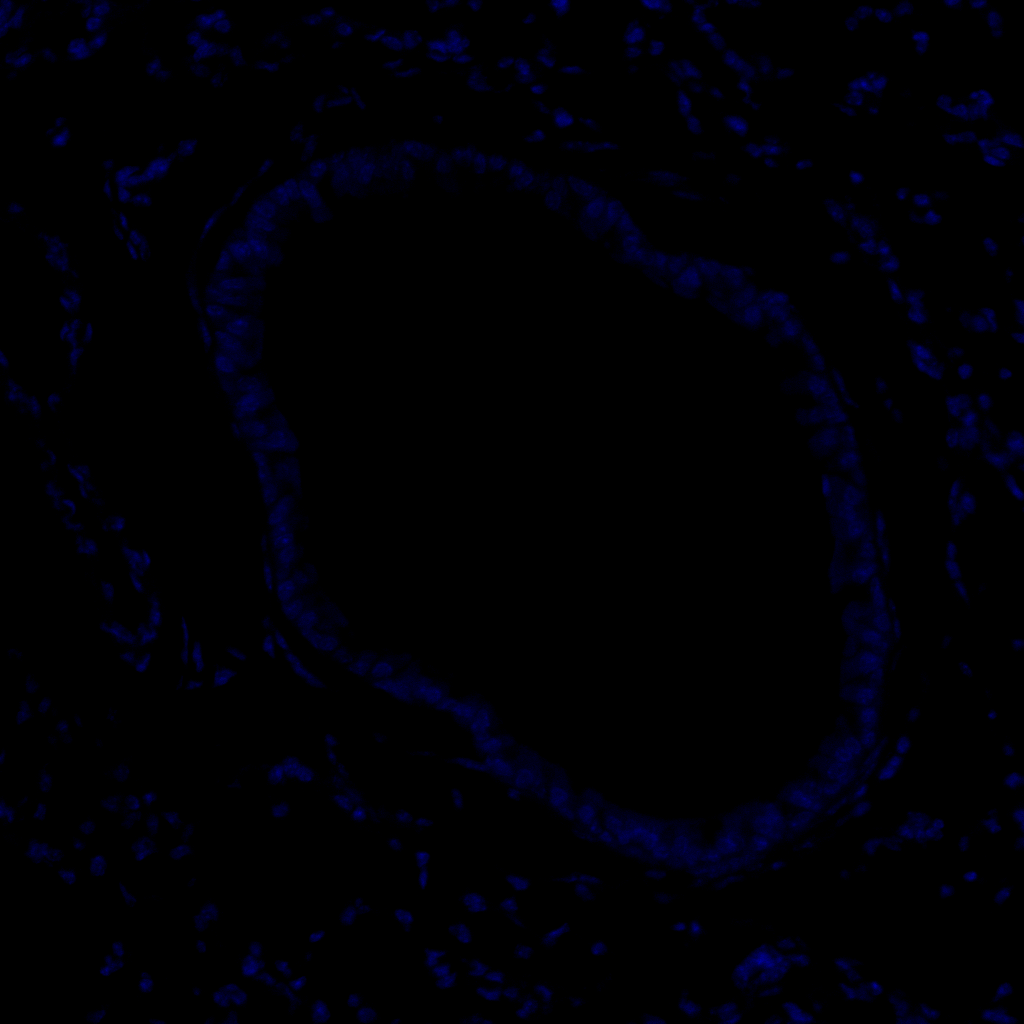

Supplement: Supplementary file 1 [file Data_Sheet_1.ZIP › Immunofluorescence/HO-1/OVA/3.tif]

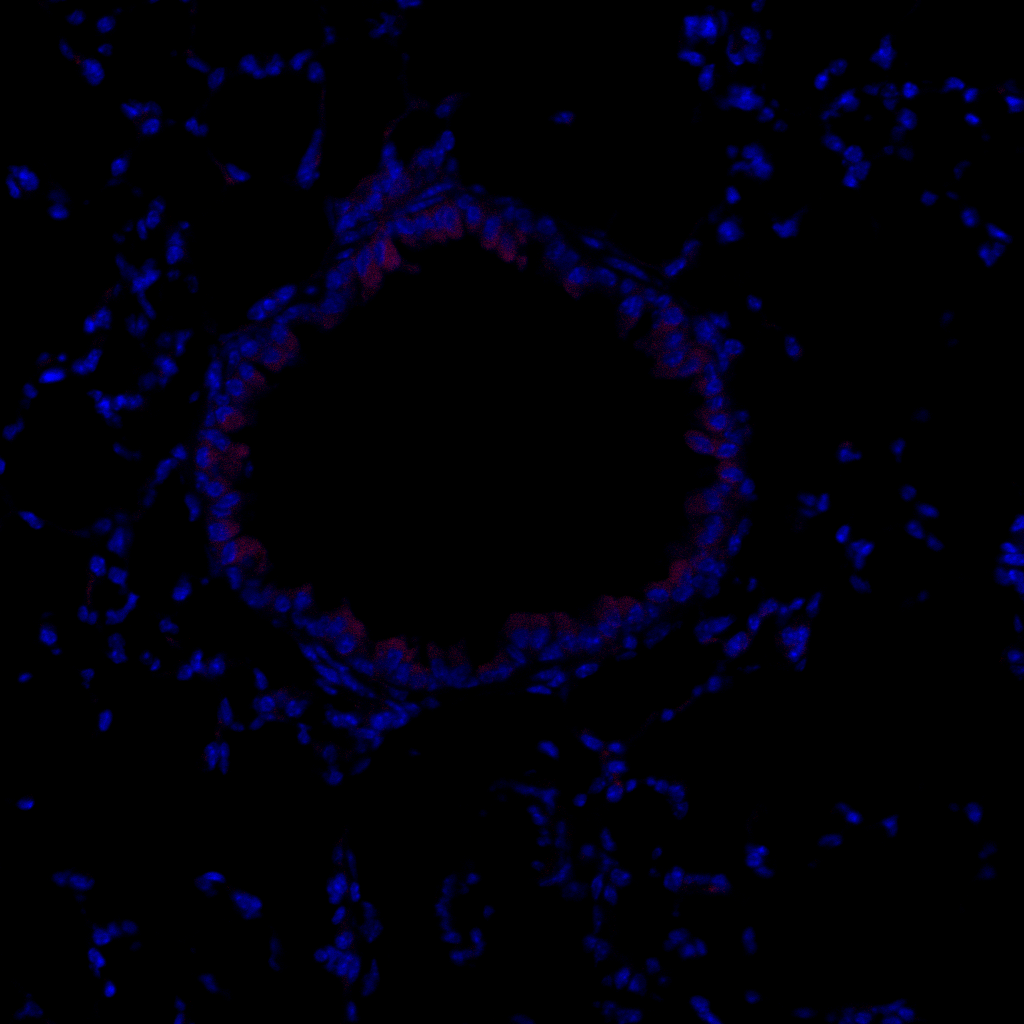

Supplement: Supplementary file 1 [file Data_Sheet_1.ZIP › Immunofluorescence/HO-1/OVA+CIH/1.tif]

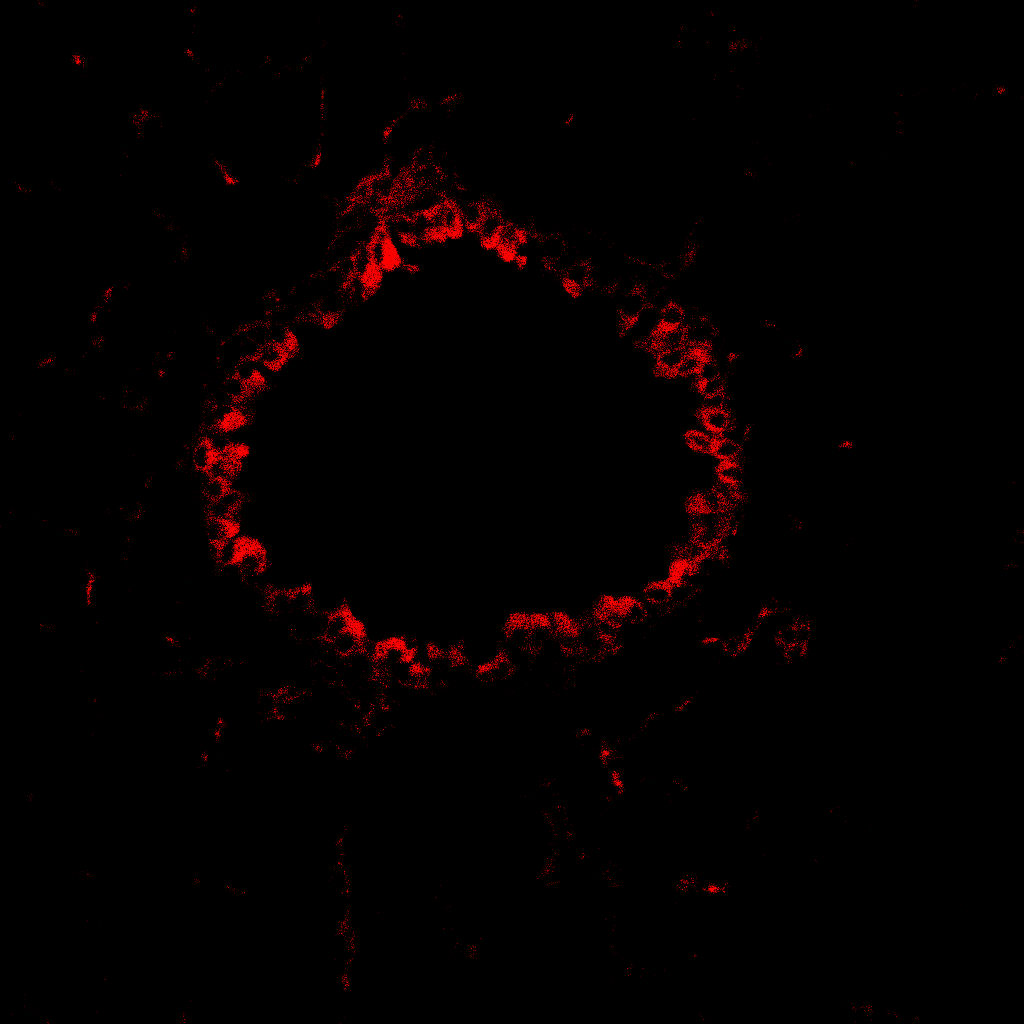

Supplement: Supplementary file 1 [file Data_Sheet_1.ZIP › Immunofluorescence/HO-1/OVA+CIH/2.tif]

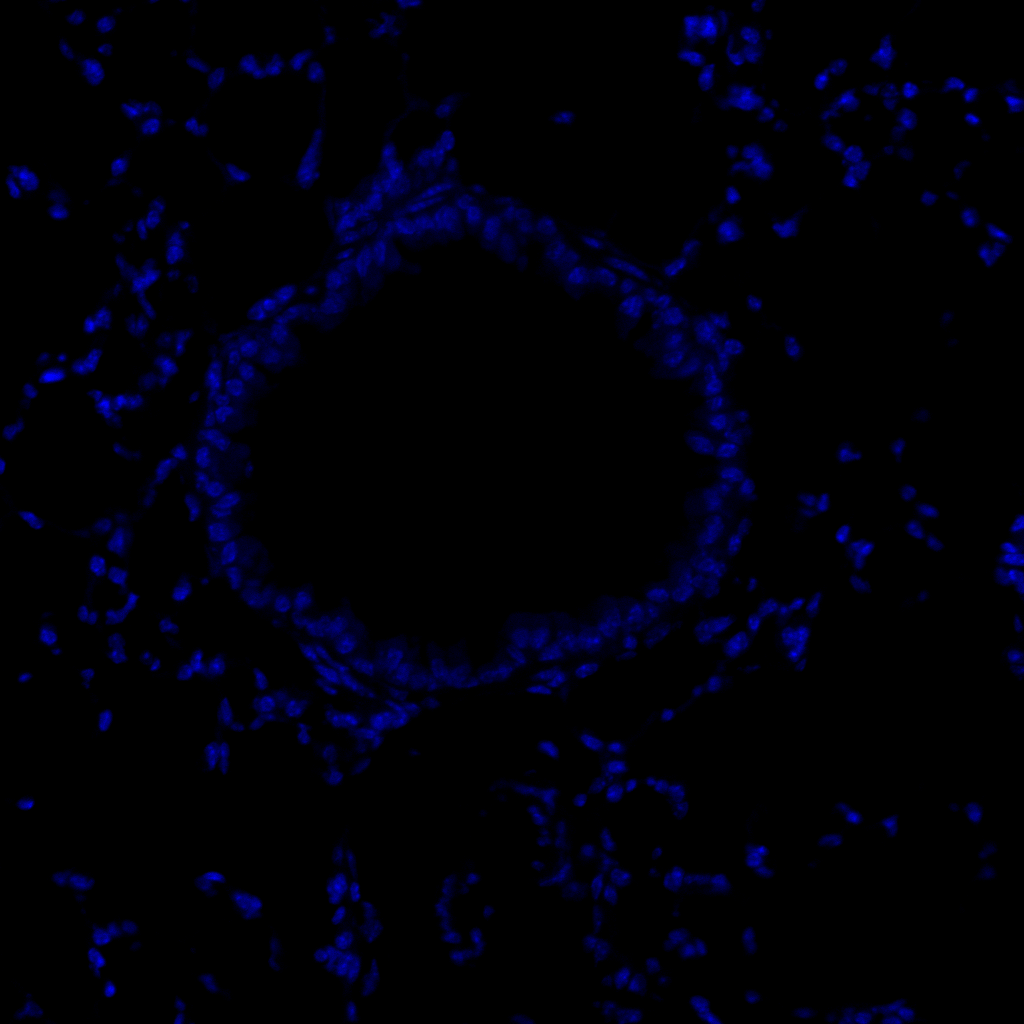

Supplement: Supplementary file 1 [file Data_Sheet_1.ZIP › Immunofluorescence/HO-1/OVA+CIH/3.tif]

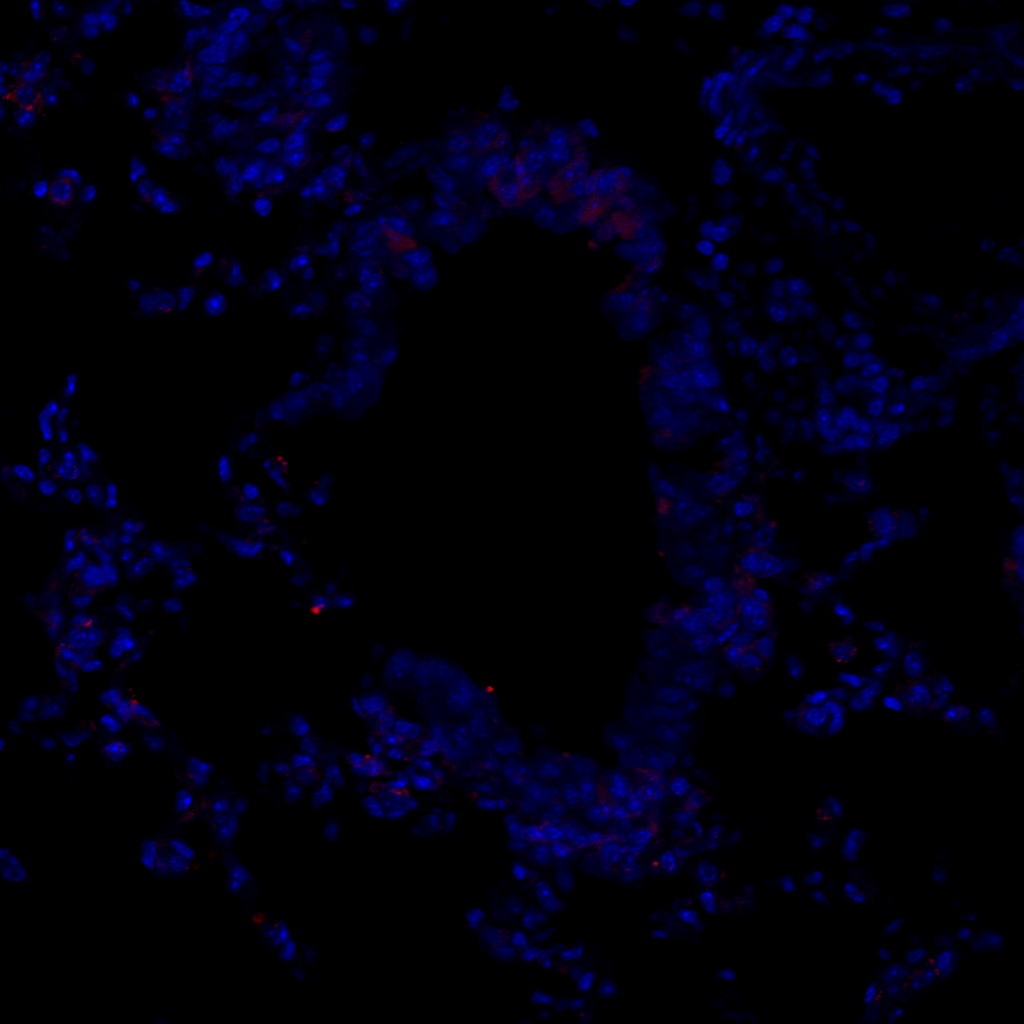

Supplement: Supplementary file 1 [file Data_Sheet_1.ZIP › Immunofluorescence/HO-1/OVA+CIH+DEX/1.tif]

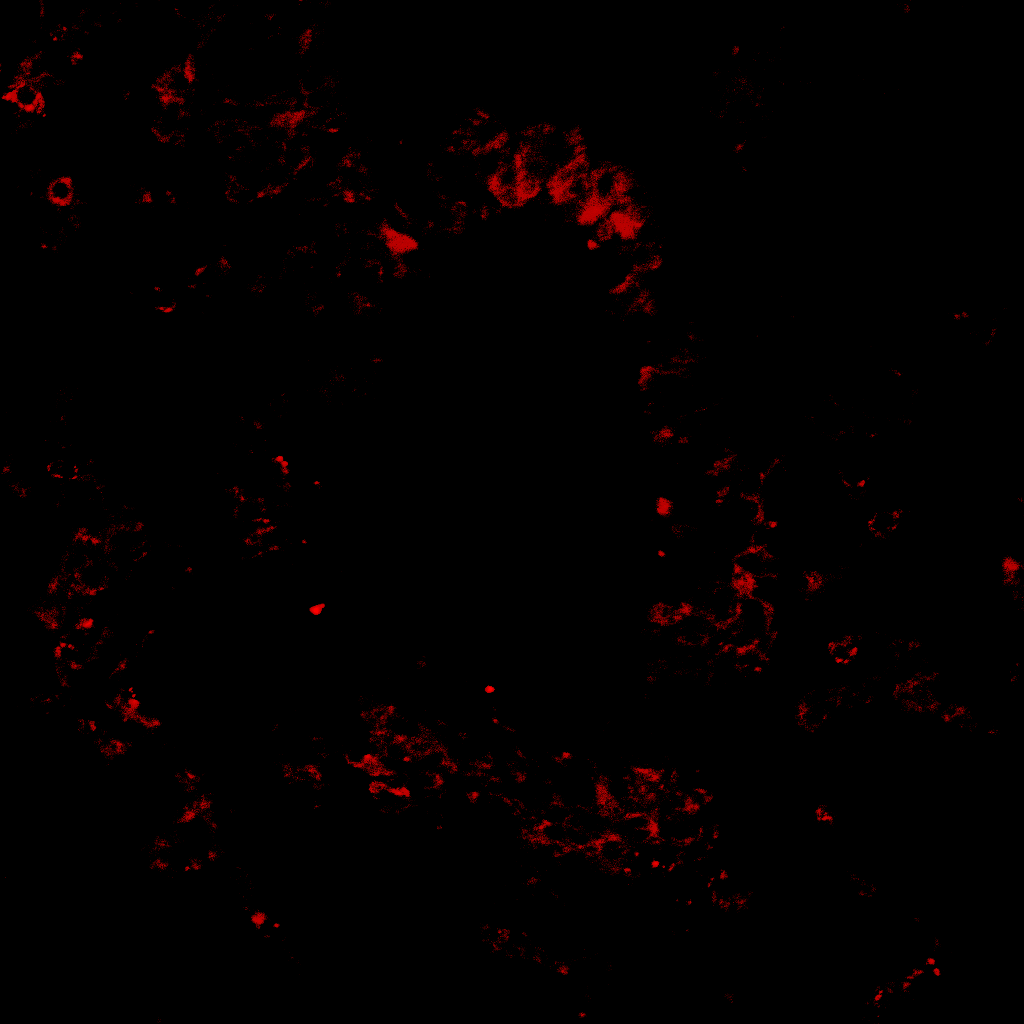

Supplement: Supplementary file 1 [file Data_Sheet_1.ZIP › Immunofluorescence/HO-1/OVA+CIH+DEX/2.tif]

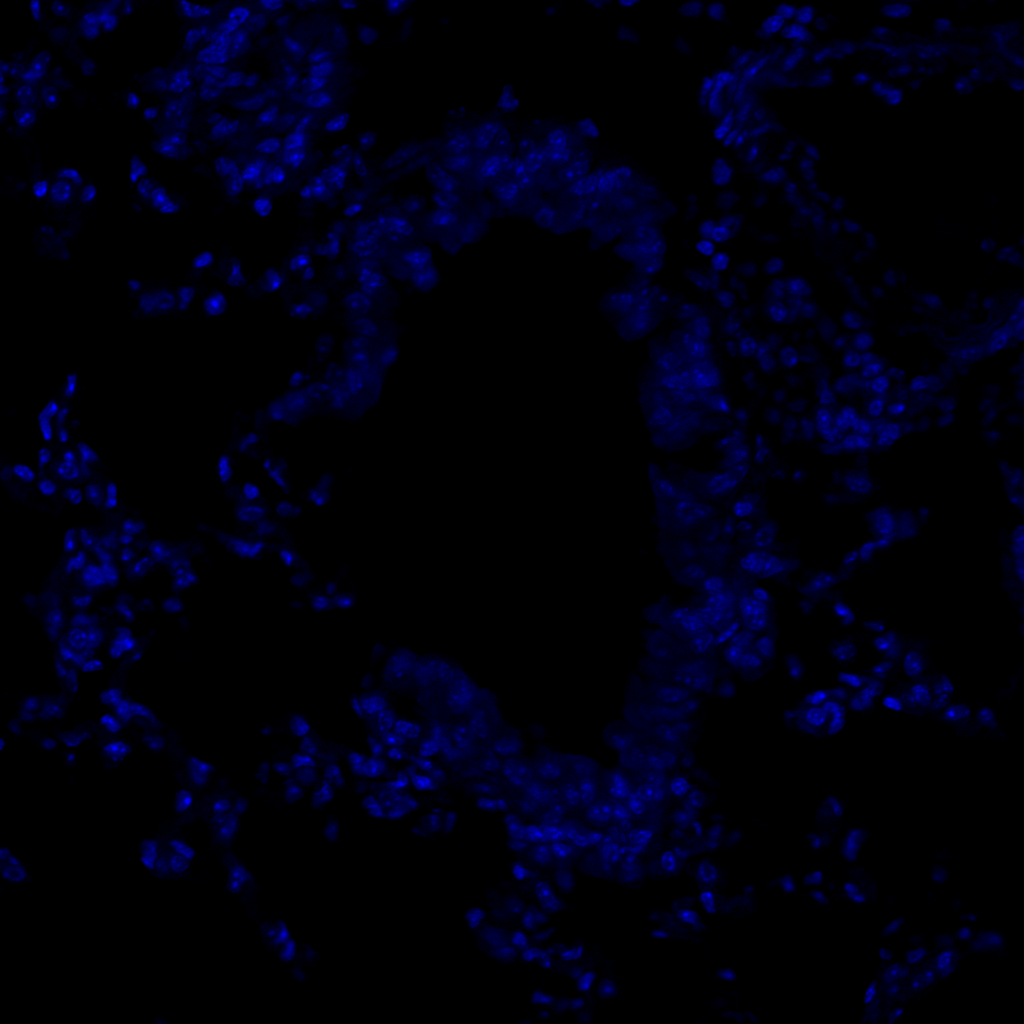

Supplement: Supplementary file 1 [file Data_Sheet_1.ZIP › Immunofluorescence/HO-1/OVA+CIH+DEX/3.tif]

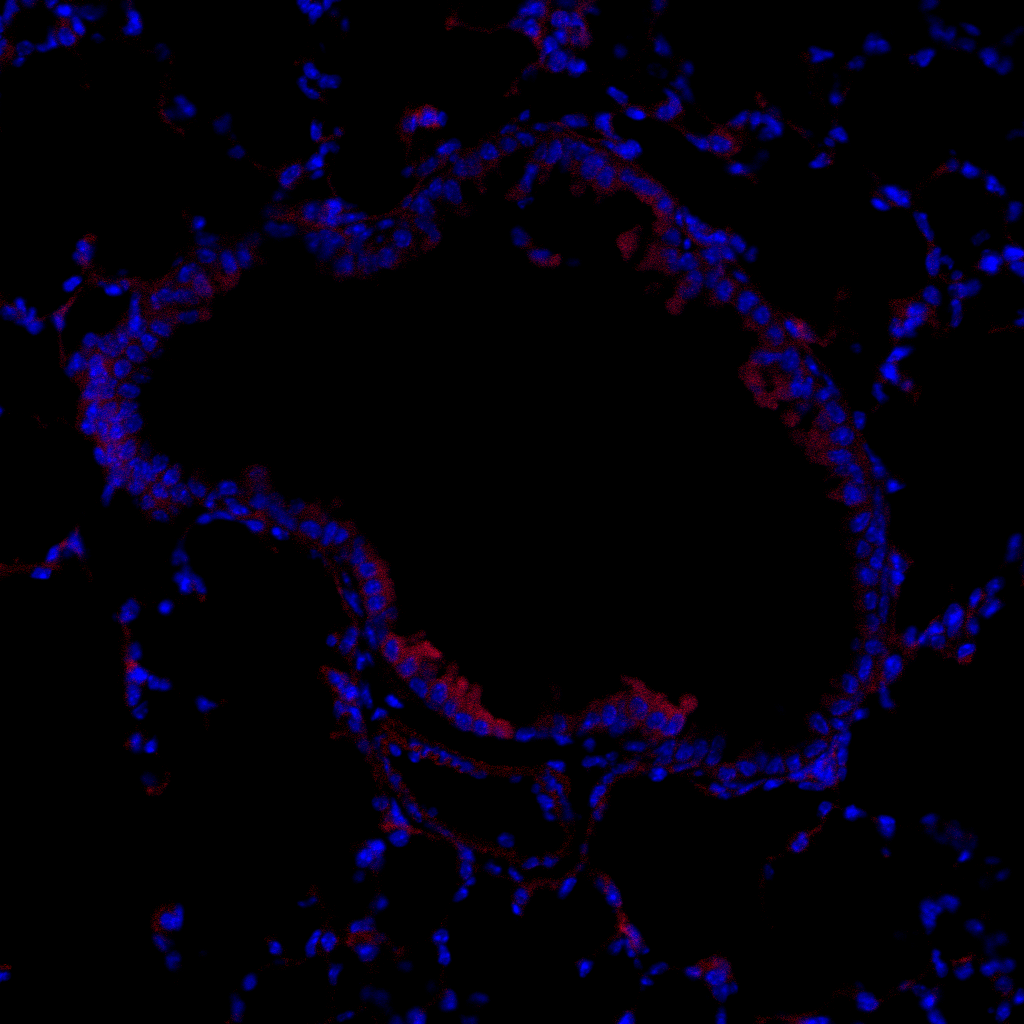

Supplement: Supplementary file 1 [file Data_Sheet_1.ZIP › Immunofluorescence/HO-1/OVA+CIH+DEX+SB/1.tif]

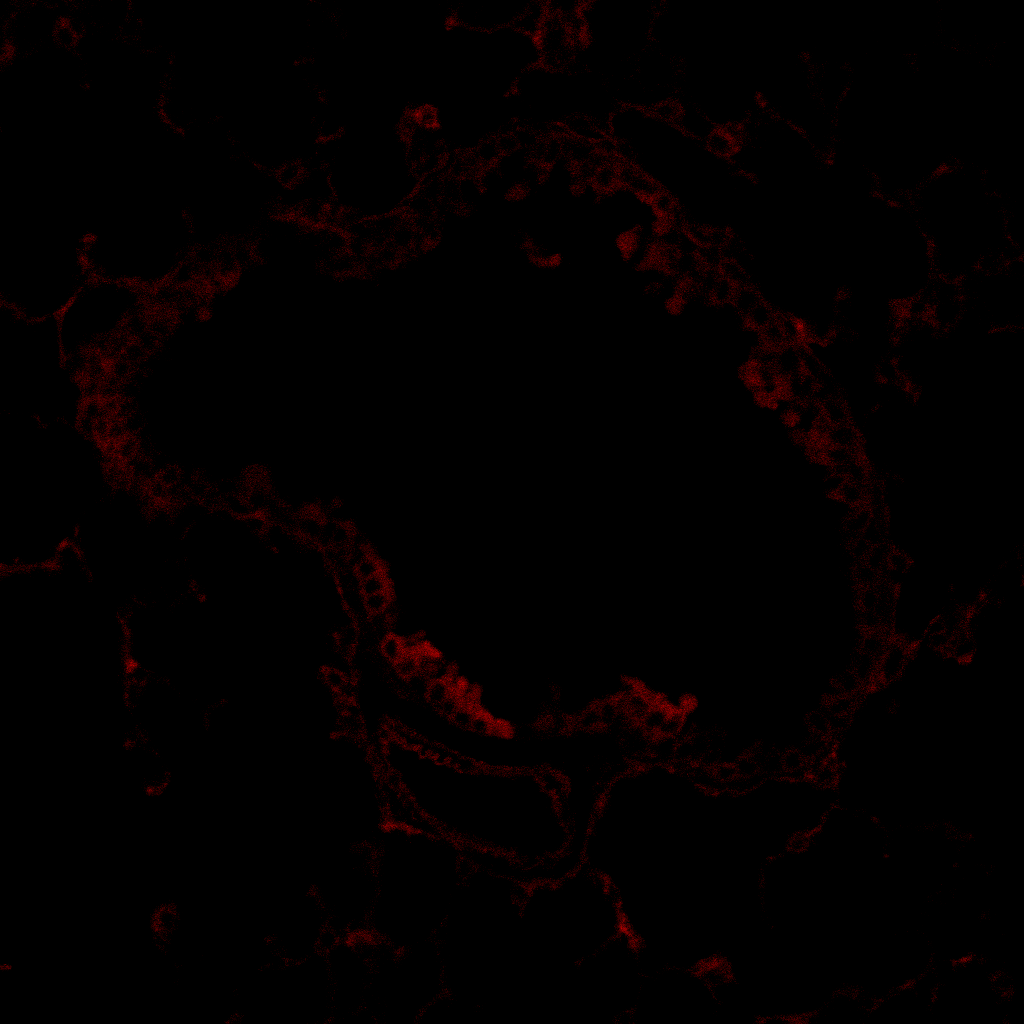

Supplement: Supplementary file 1 [file Data_Sheet_1.ZIP › Immunofluorescence/HO-1/OVA+CIH+DEX+SB/2.tif]

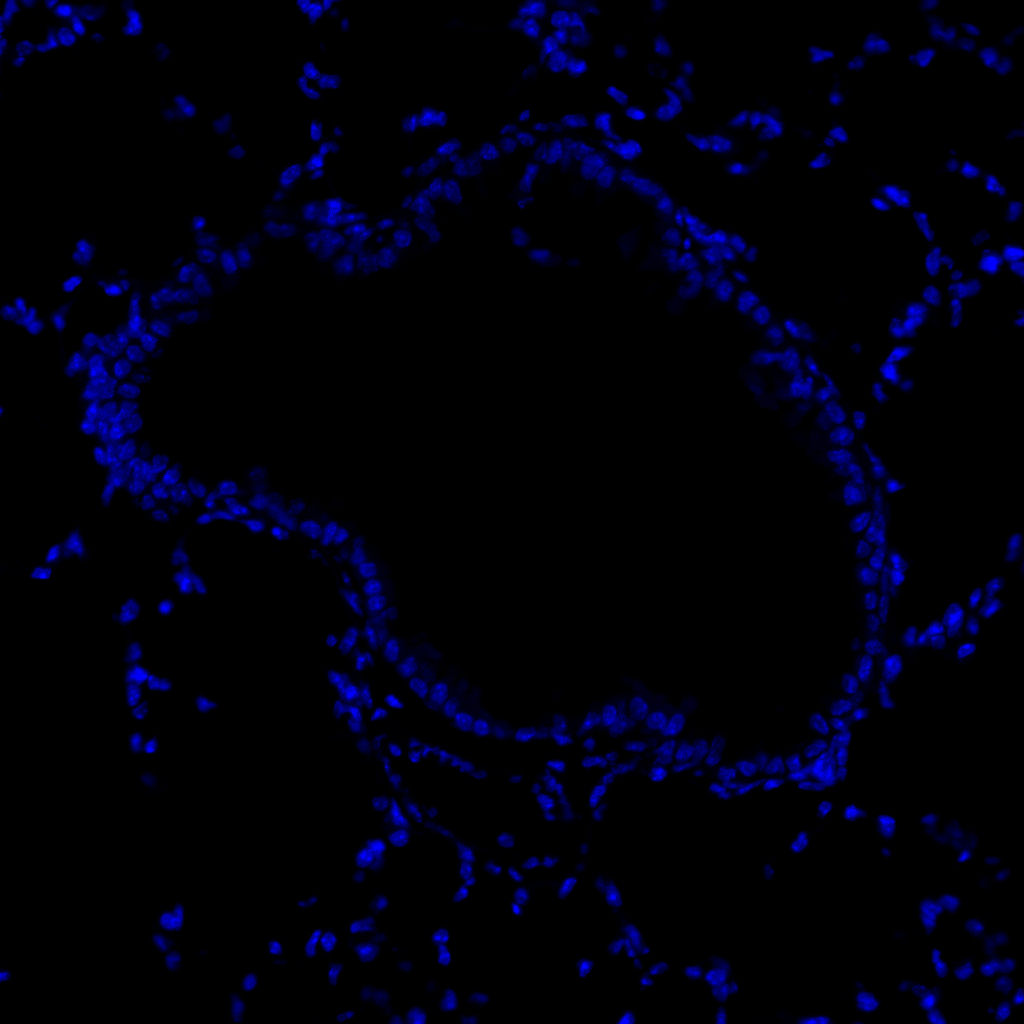

Supplement: Supplementary file 1 [file Data_Sheet_1.ZIP › Immunofluorescence/HO-1/OVA+CIH+DEX+SB/3.tif]

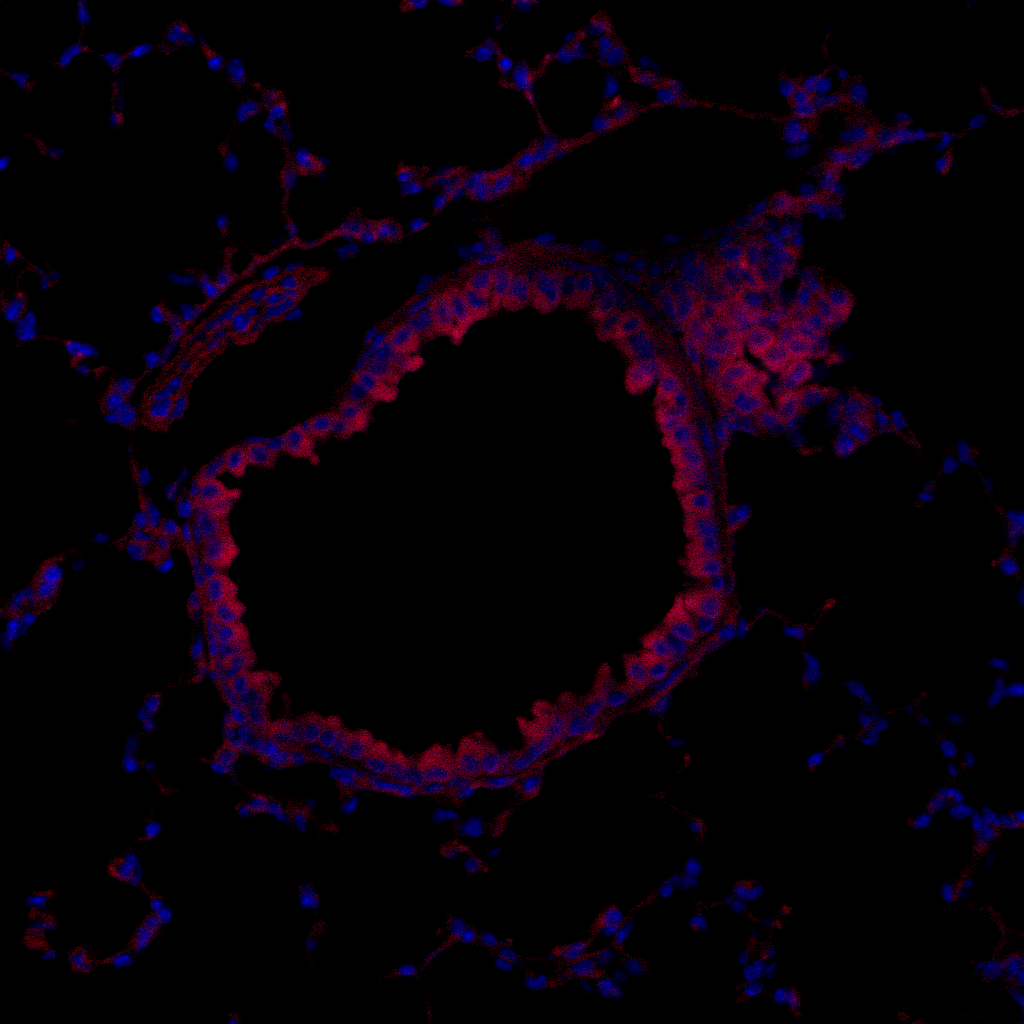

Supplement: Supplementary file 1 [file Data_Sheet_1.ZIP › Immunofluorescence/HO-1/OVA+DEX/1.tif]

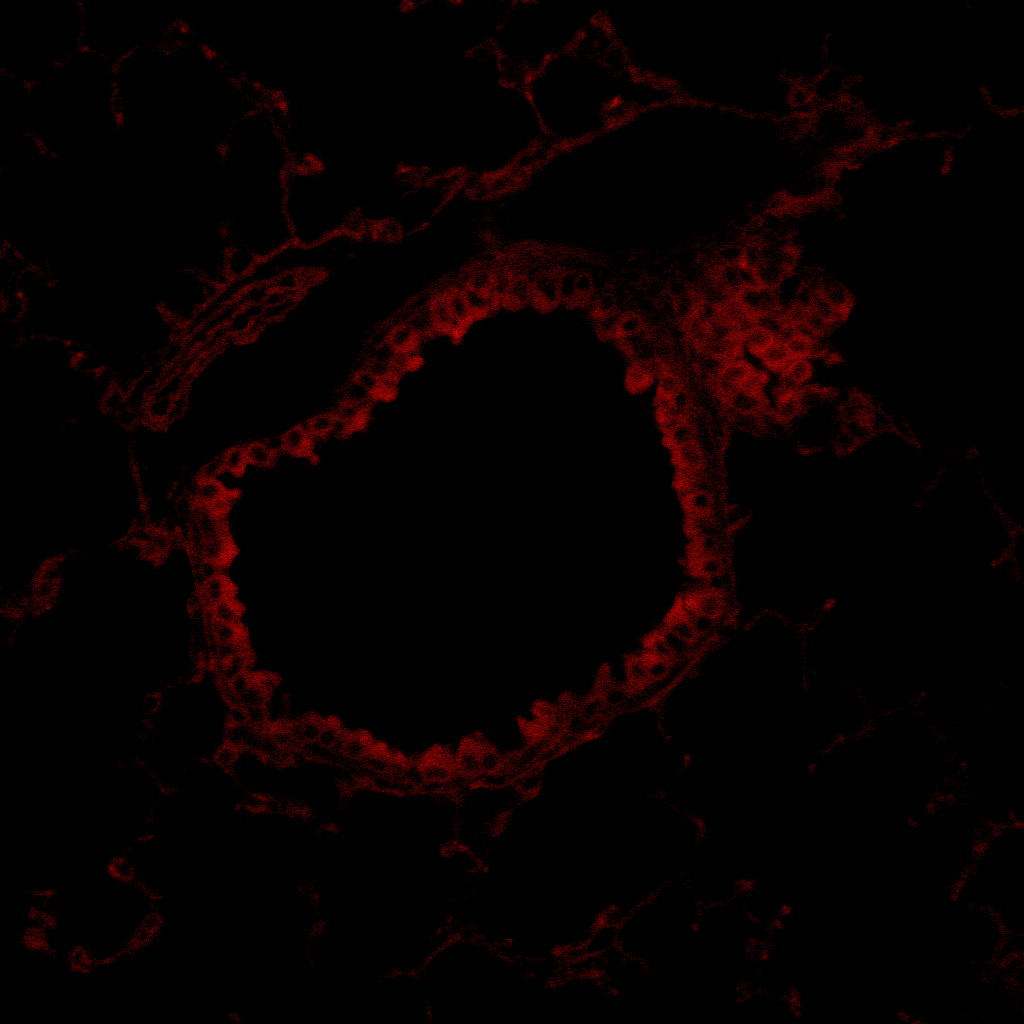

Supplement: Supplementary file 1 [file Data_Sheet_1.ZIP › Immunofluorescence/HO-1/OVA+DEX/2.tif]

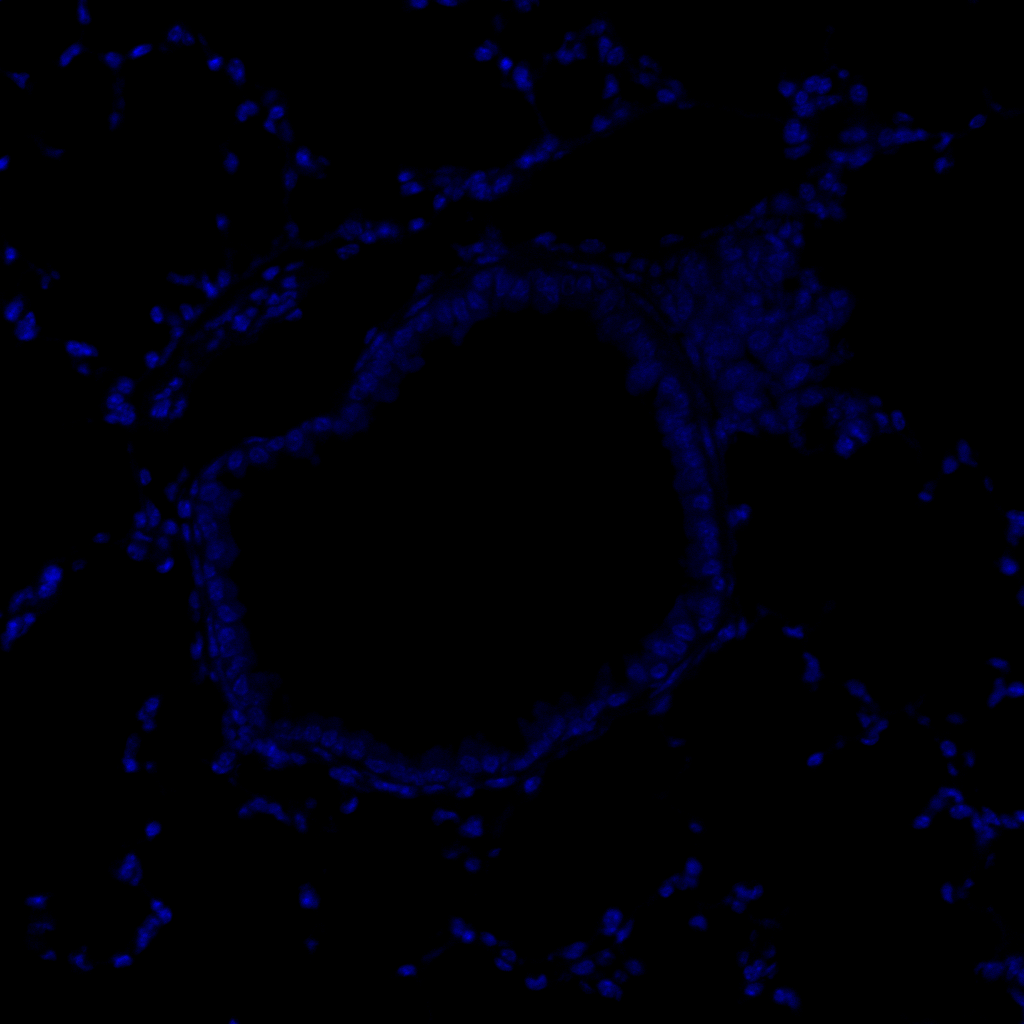

Supplement: Supplementary file 1 [file Data_Sheet_1.ZIP › Immunofluorescence/HO-1/OVA+DEX/3.tif]

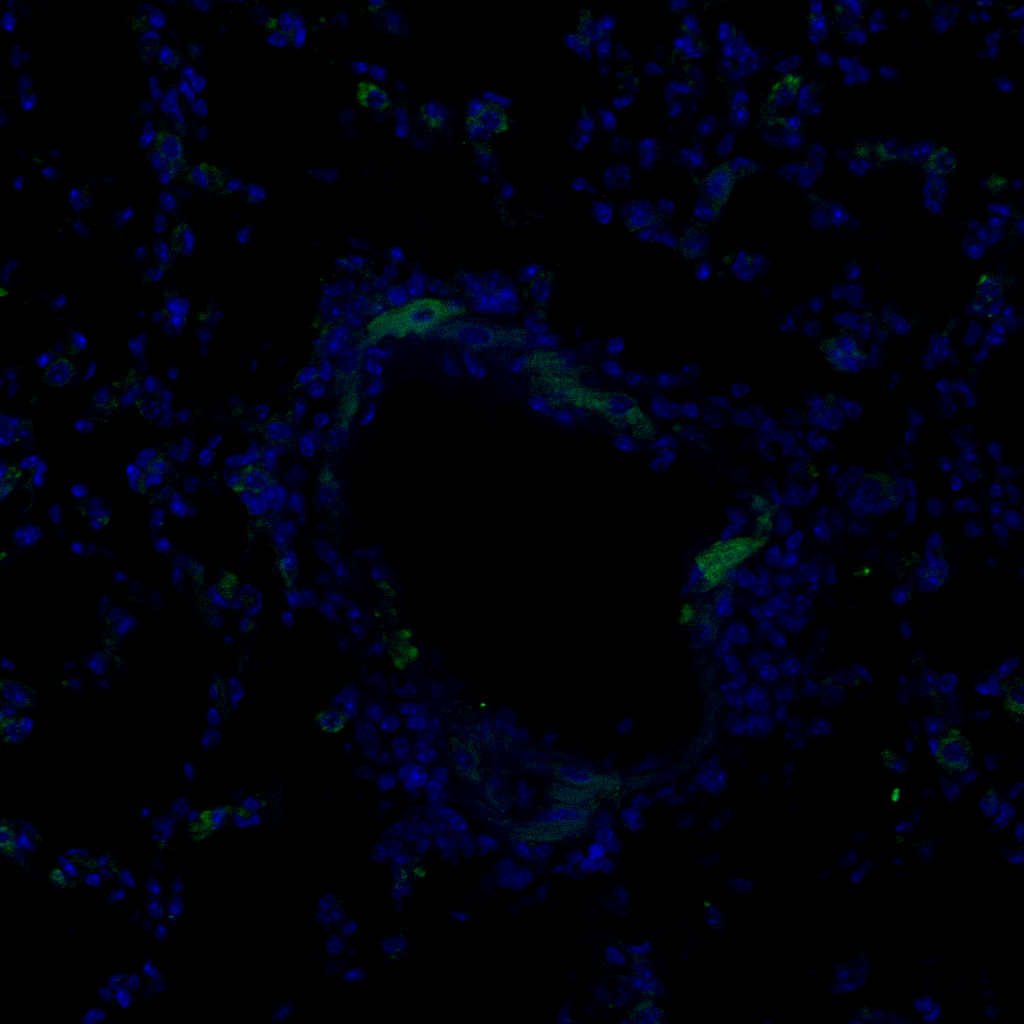

Supplement: Supplementary file 1 [file Data_Sheet_1.ZIP › Immunofluorescence/p-p38/control/1.tif]

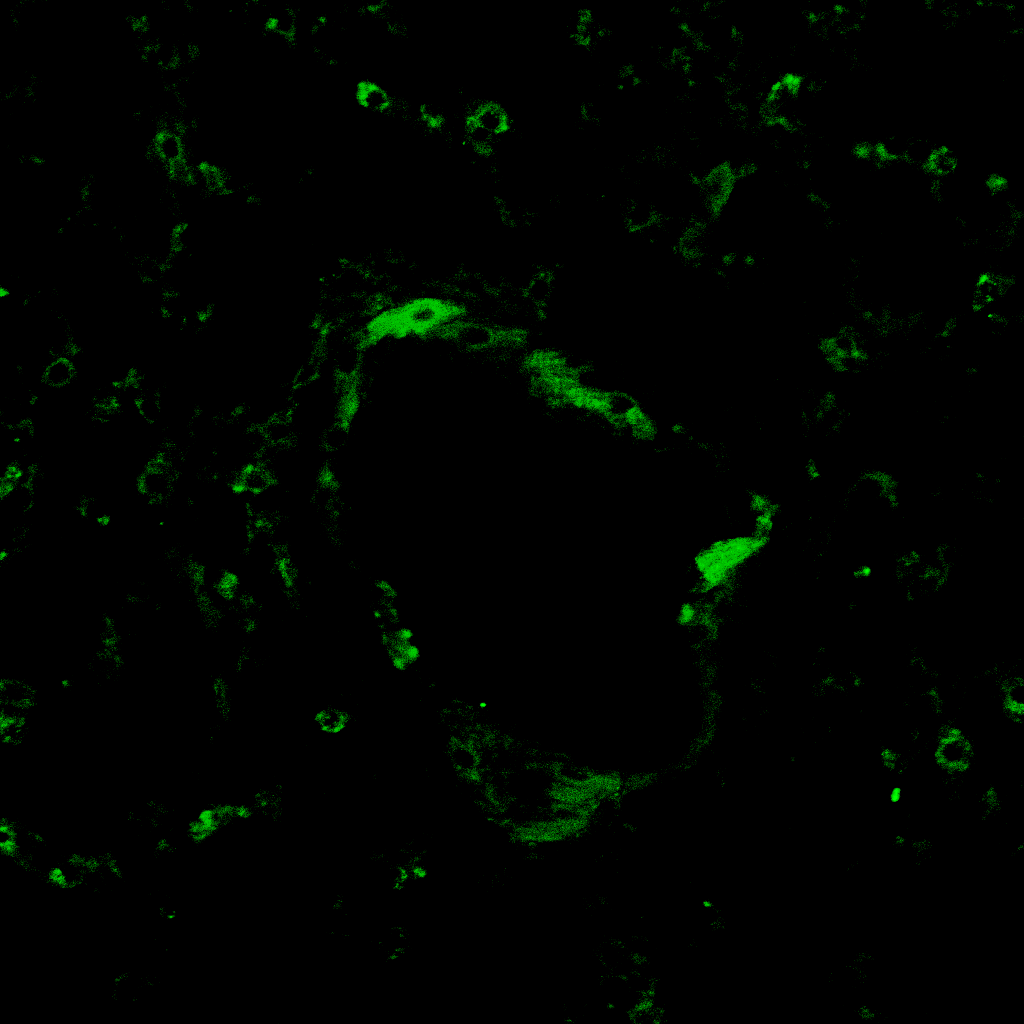

Supplement: Supplementary file 1 [file Data_Sheet_1.ZIP › Immunofluorescence/p-p38/control/2.tif]

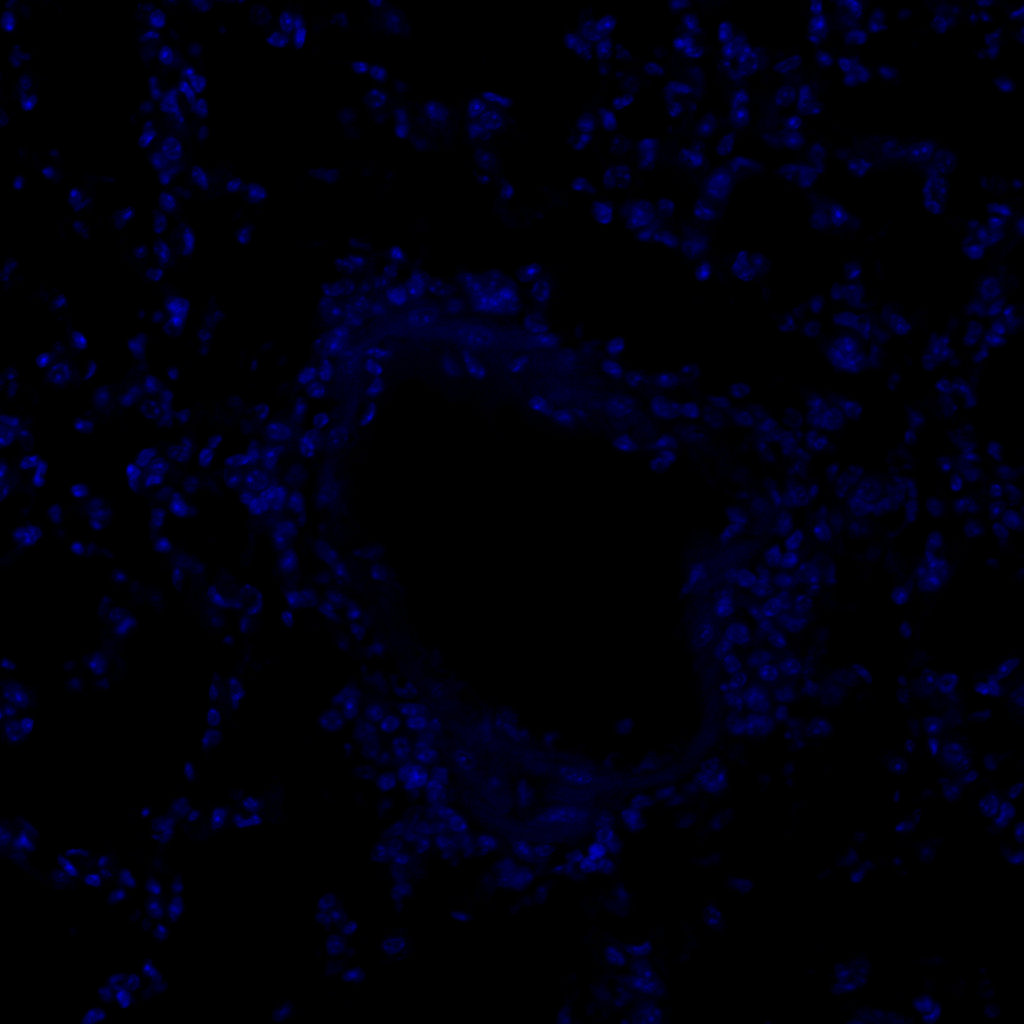

Supplement: Supplementary file 1 [file Data_Sheet_1.ZIP › Immunofluorescence/p-p38/control/3.tif]

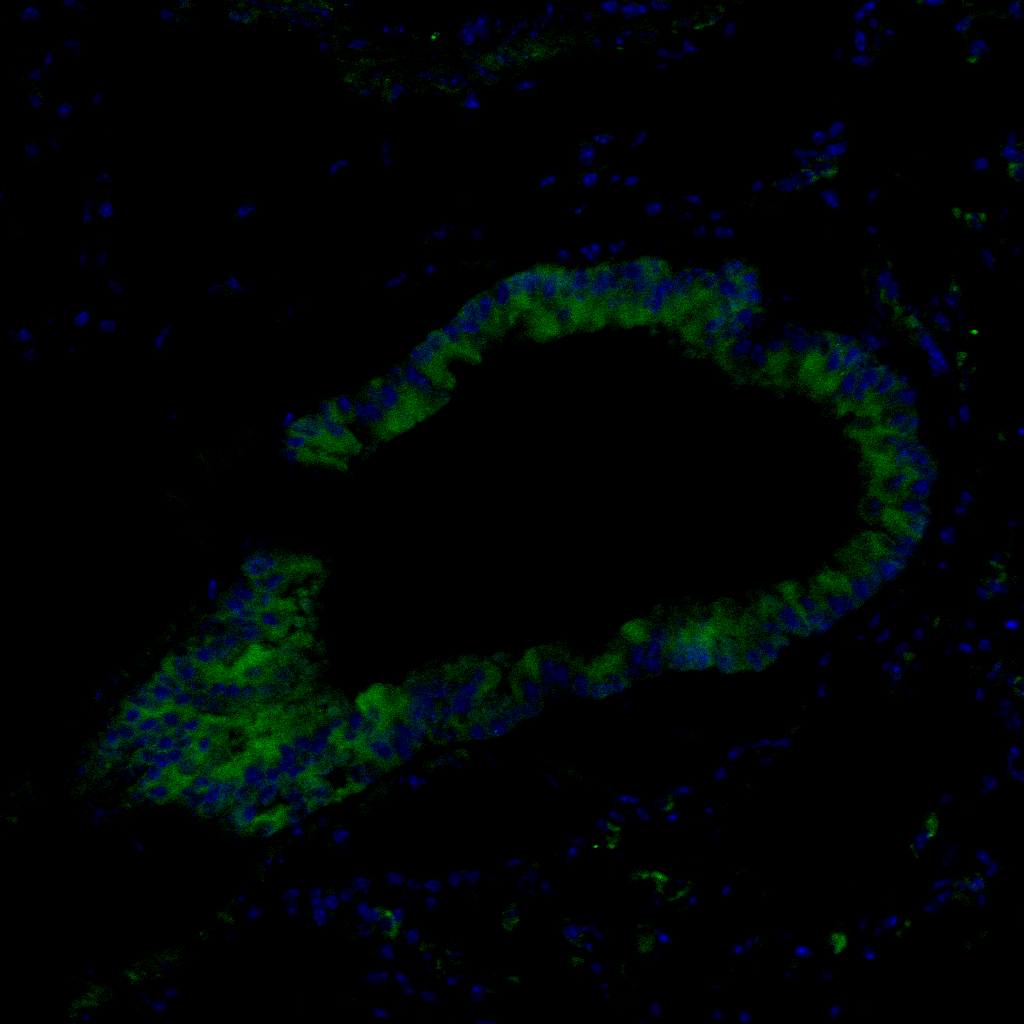

Supplement: Supplementary file 1 [file Data_Sheet_1.ZIP › Immunofluorescence/p-p38/OVA/1.tif]

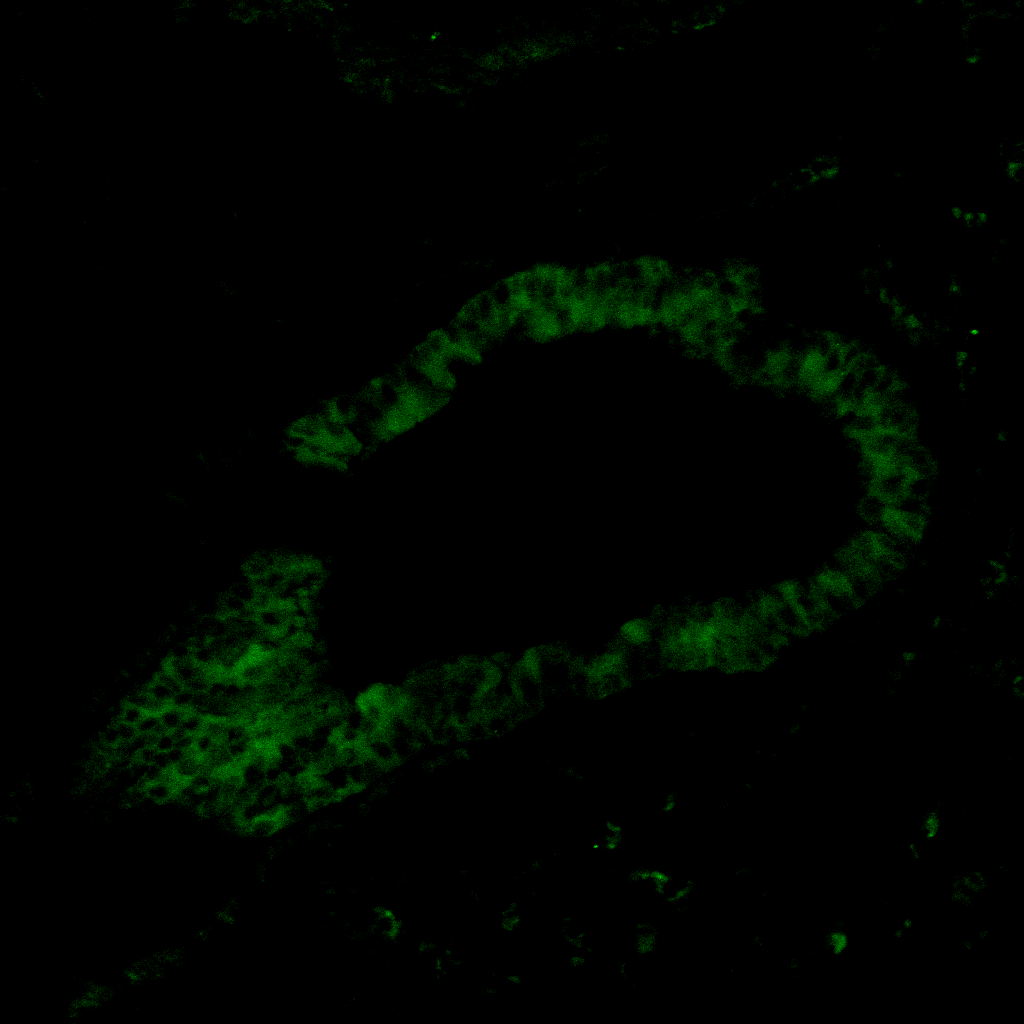

Supplement: Supplementary file 1 [file Data_Sheet_1.ZIP › Immunofluorescence/p-p38/OVA/2.tif]

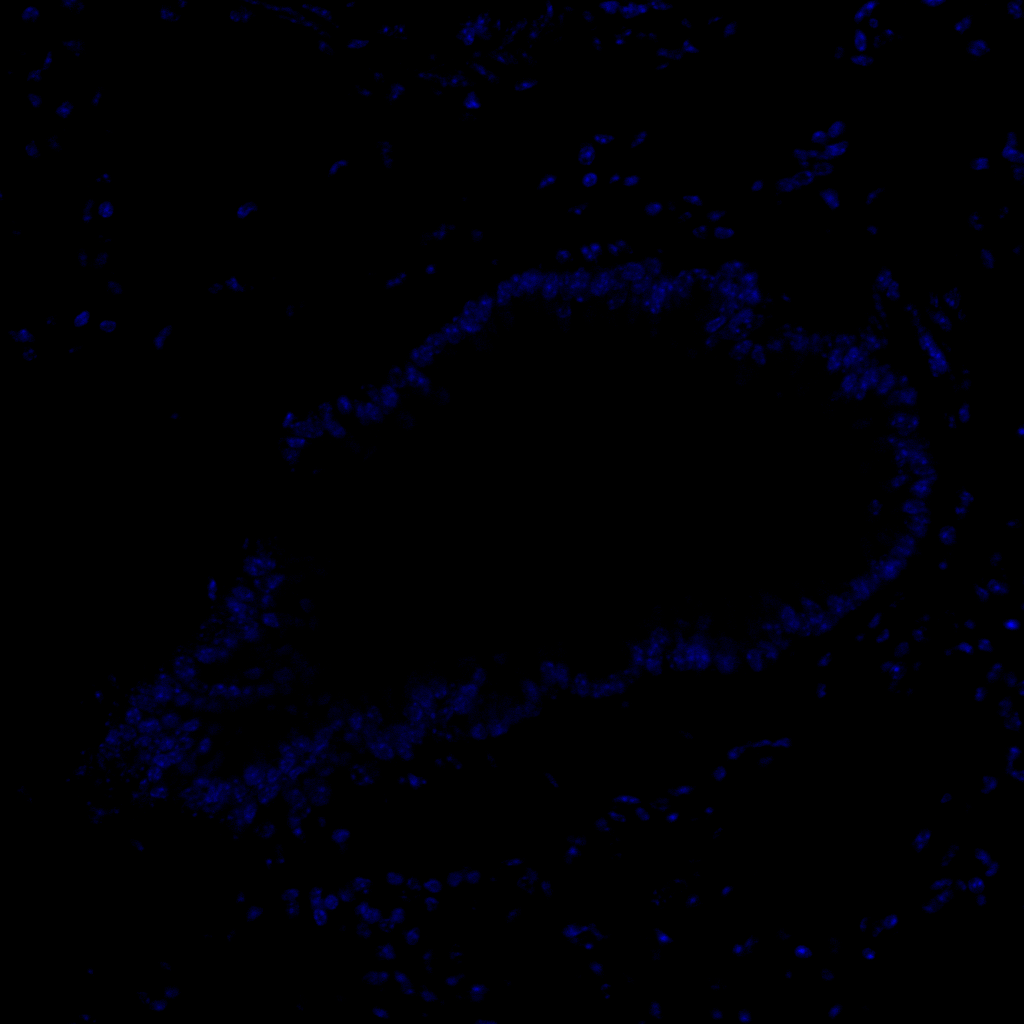

Supplement: Supplementary file 1 [file Data_Sheet_1.ZIP › Immunofluorescence/p-p38/OVA/3.tif]

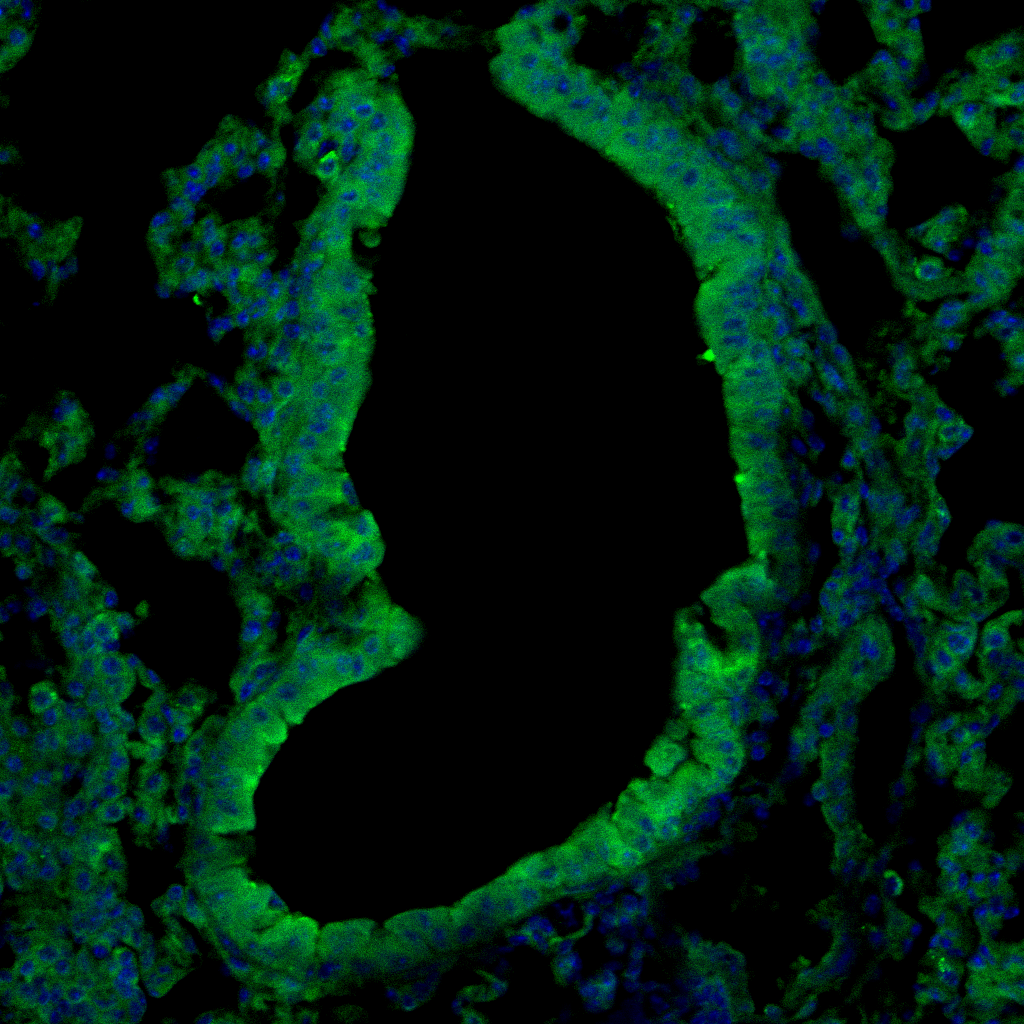

Supplement: Supplementary file 1 [file Data_Sheet_1.ZIP › Immunofluorescence/p-p38/OVA+CIH/1.tif]

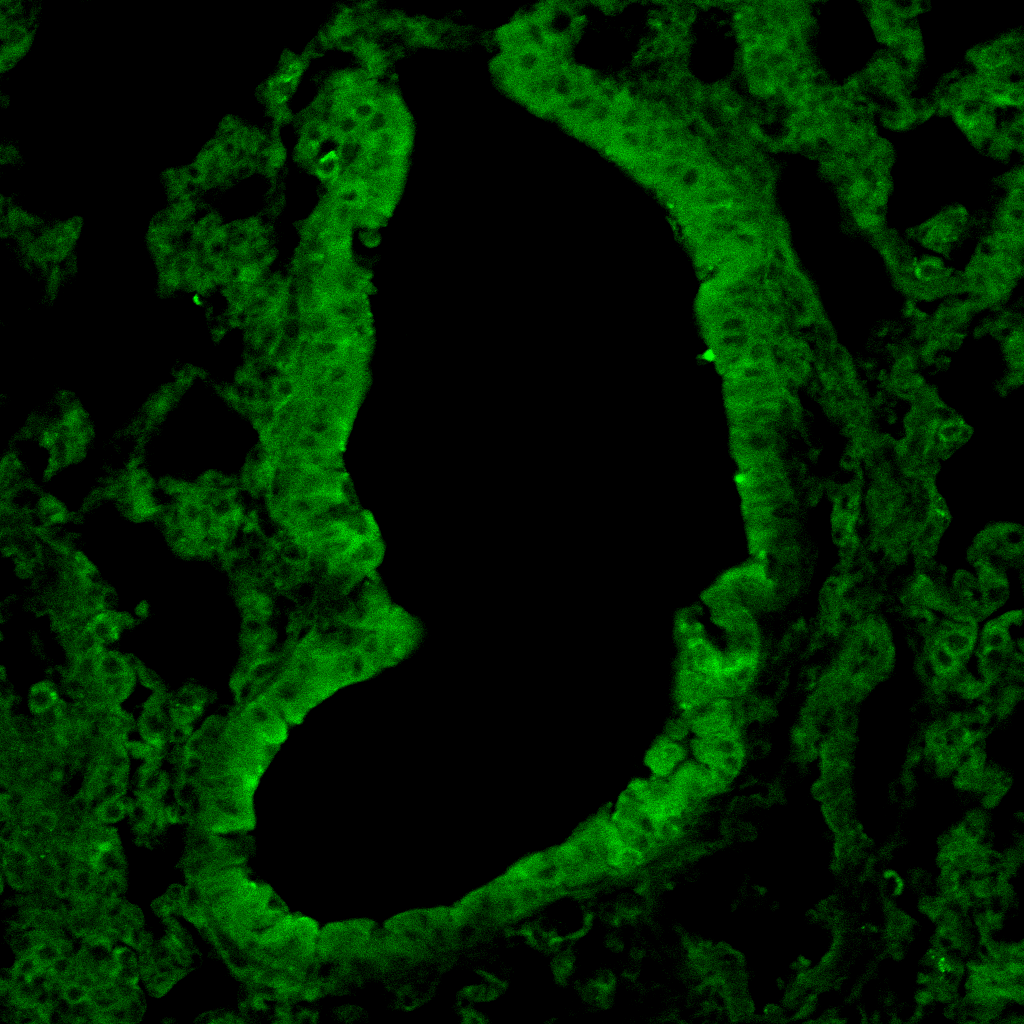

Supplement: Supplementary file 1 [file Data_Sheet_1.ZIP › Immunofluorescence/p-p38/OVA+CIH/2.tif]

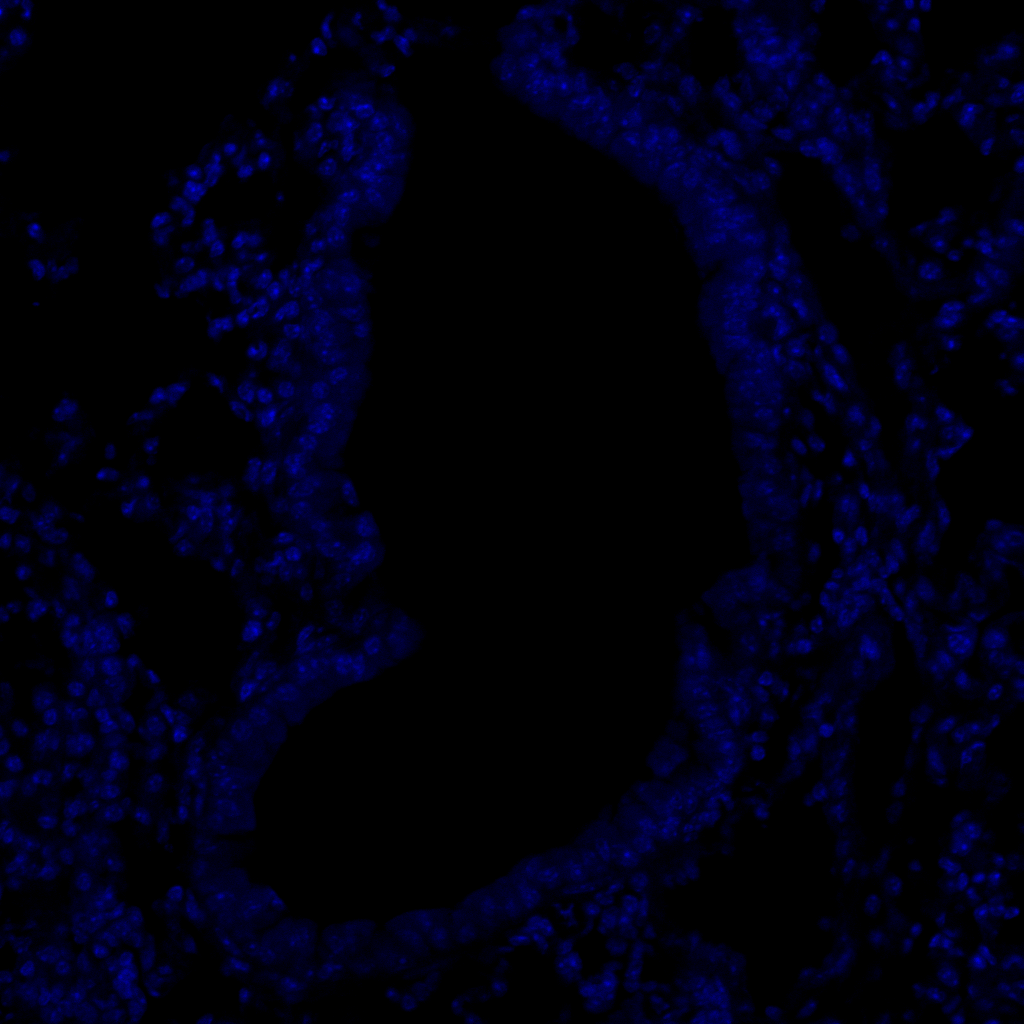

Supplement: Supplementary file 1 [file Data_Sheet_1.ZIP › Immunofluorescence/p-p38/OVA+CIH/3.tif]

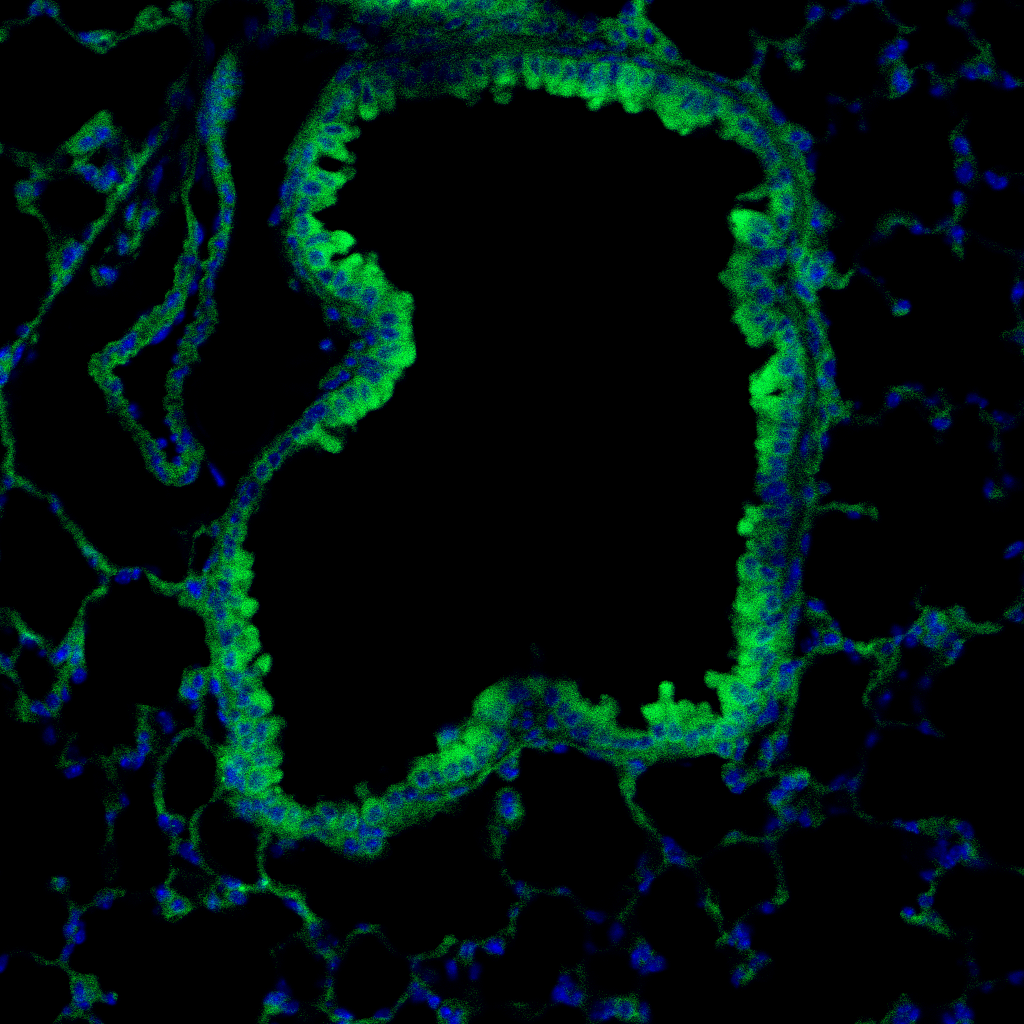

Supplement: Supplementary file 1 [file Data_Sheet_1.ZIP › Immunofluorescence/p-p38/OVA+CIH+DEX/1.tif]

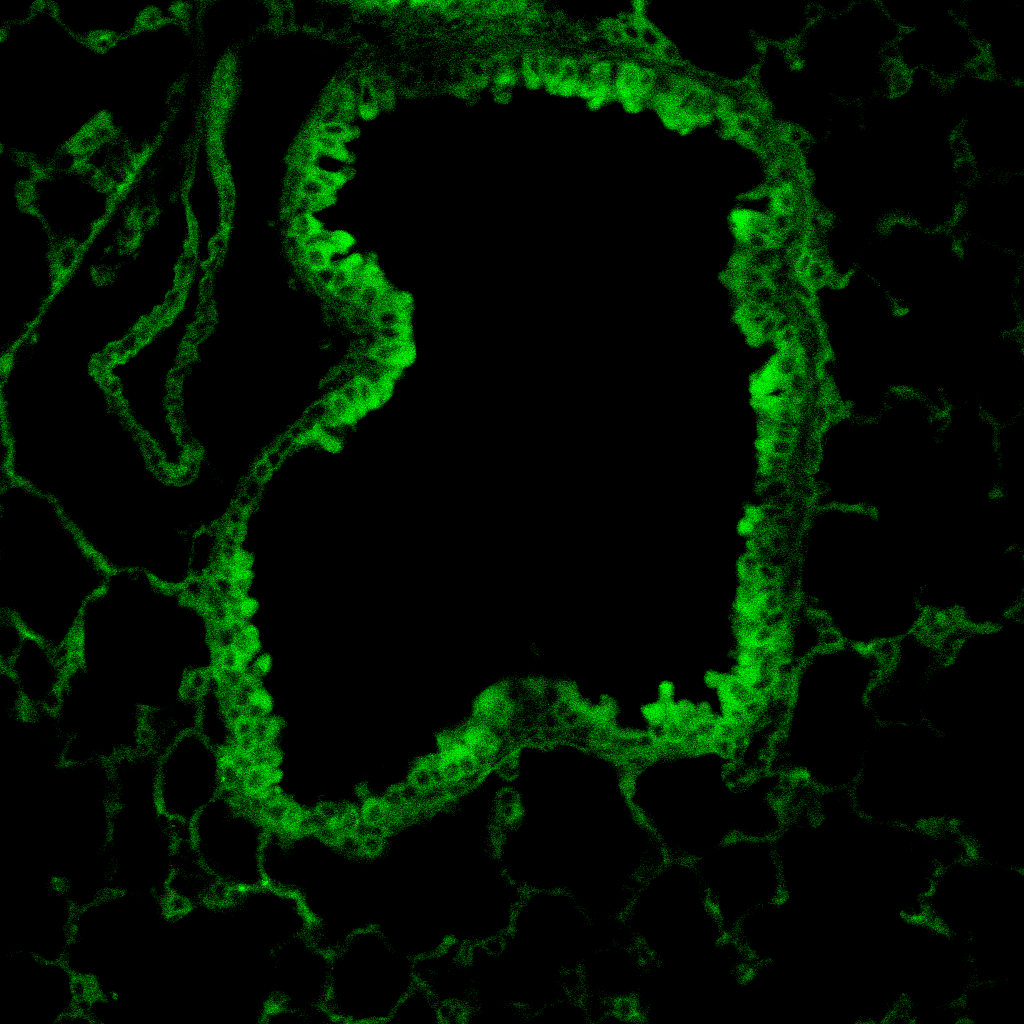

Supplement: Supplementary file 1 [file Data_Sheet_1.ZIP › Immunofluorescence/p-p38/OVA+CIH+DEX/2.tif]

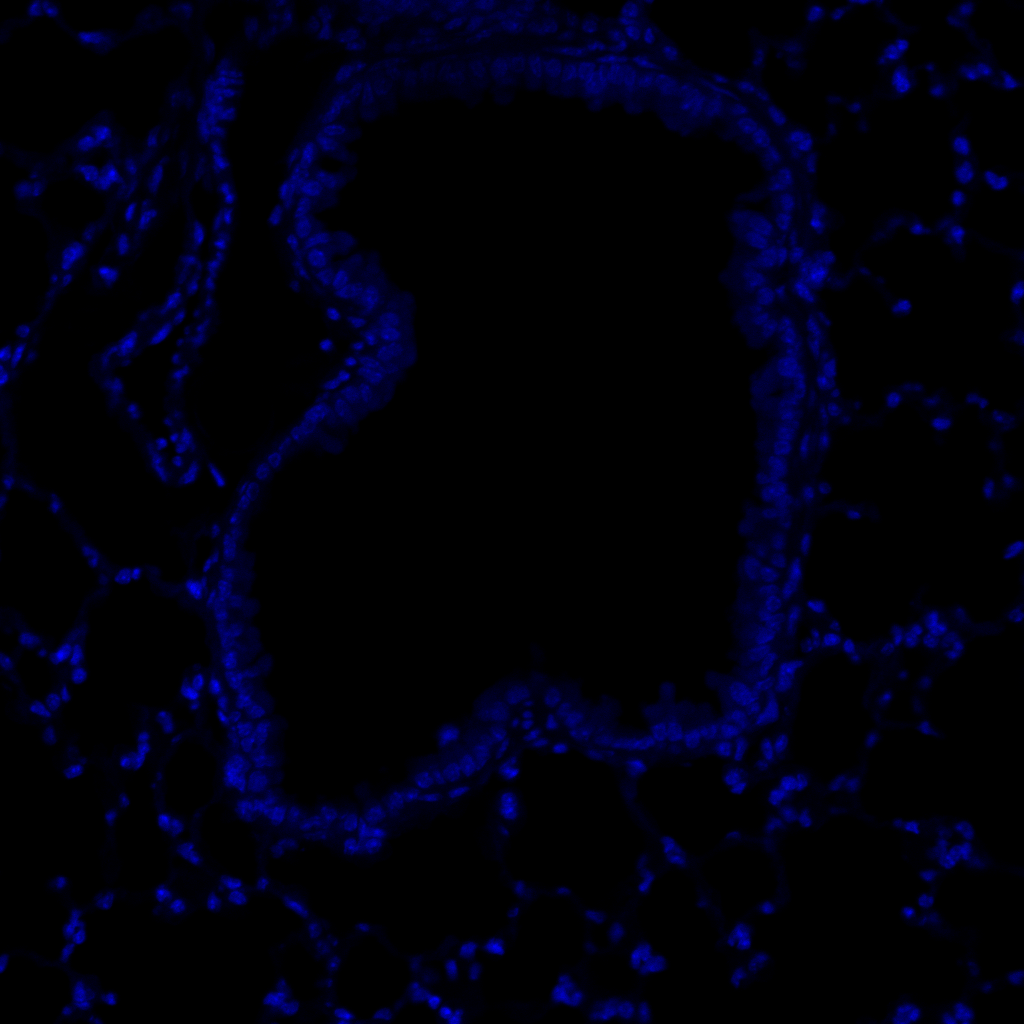

Supplement: Supplementary file 1 [file Data_Sheet_1.ZIP › Immunofluorescence/p-p38/OVA+CIH+DEX/3.tif]

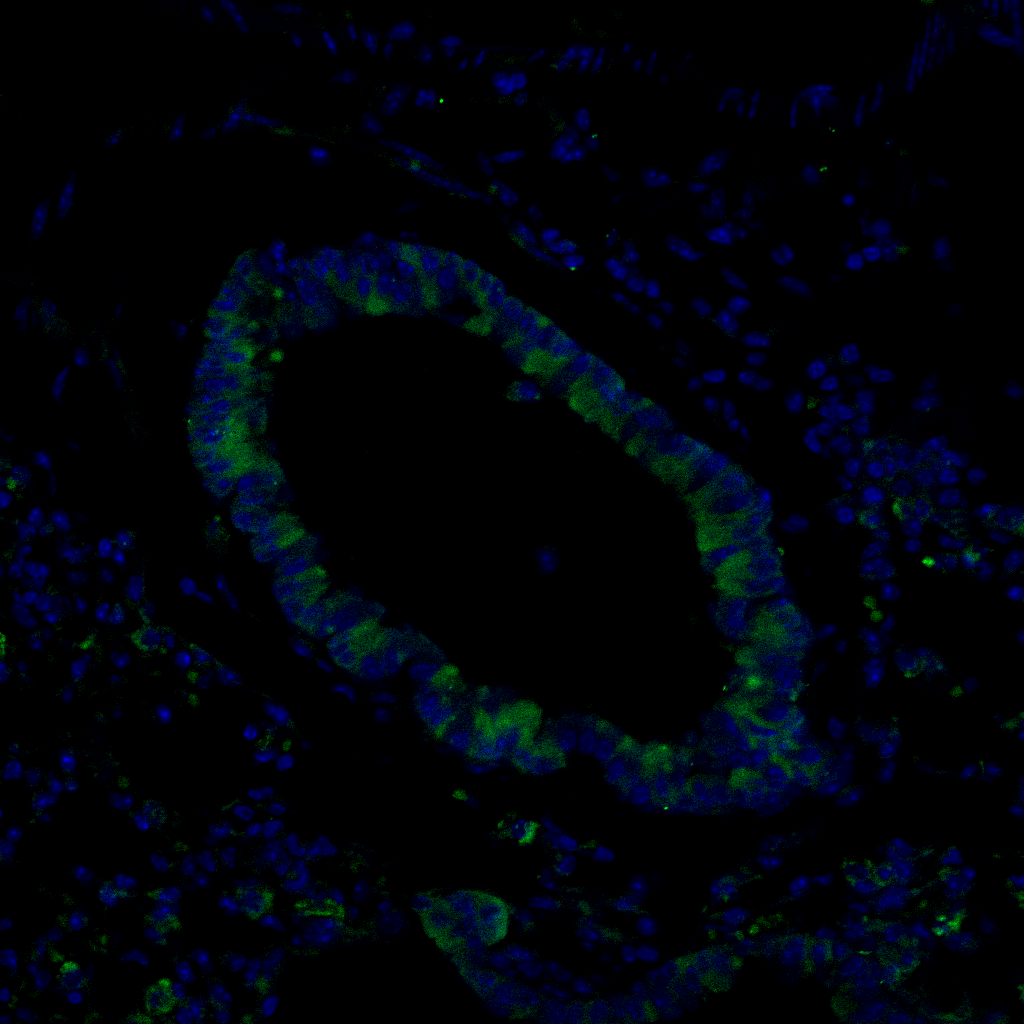

Supplement: Supplementary file 1 [file Data_Sheet_1.ZIP › Immunofluorescence/p-p38/OVA+CIH+DEX+SB/1.tif]

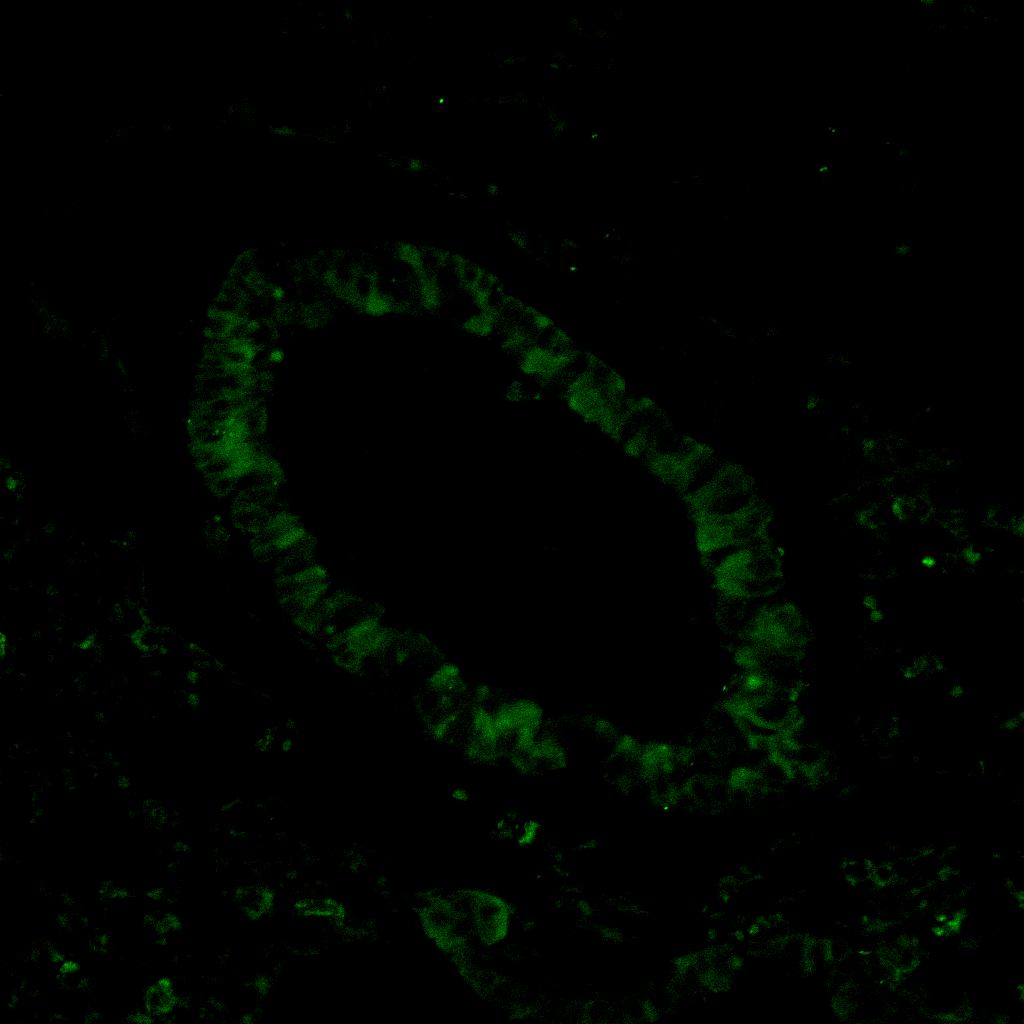

Supplement: Supplementary file 1 [file Data_Sheet_1.ZIP › Immunofluorescence/p-p38/OVA+CIH+DEX+SB/2.tif]

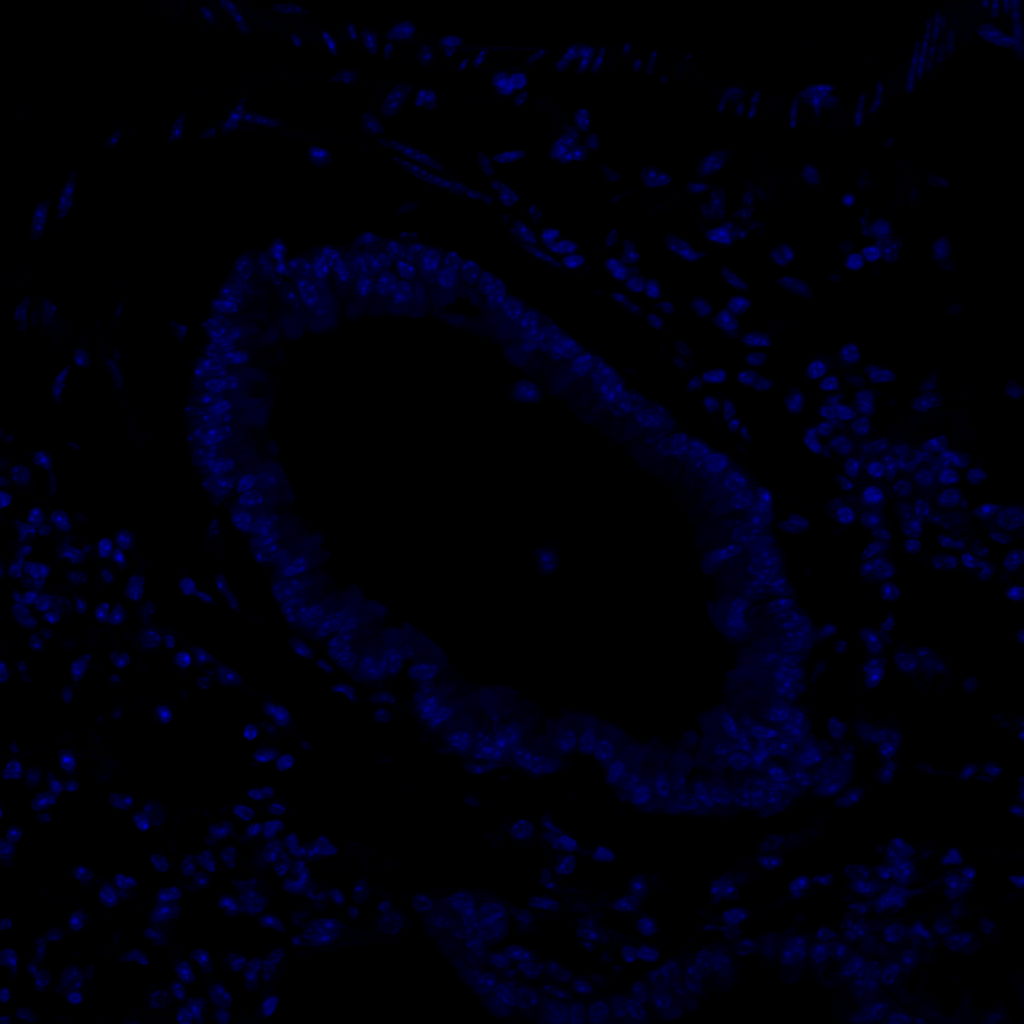

Supplement: Supplementary file 1 [file Data_Sheet_1.ZIP › Immunofluorescence/p-p38/OVA+CIH+DEX+SB/3.tif]

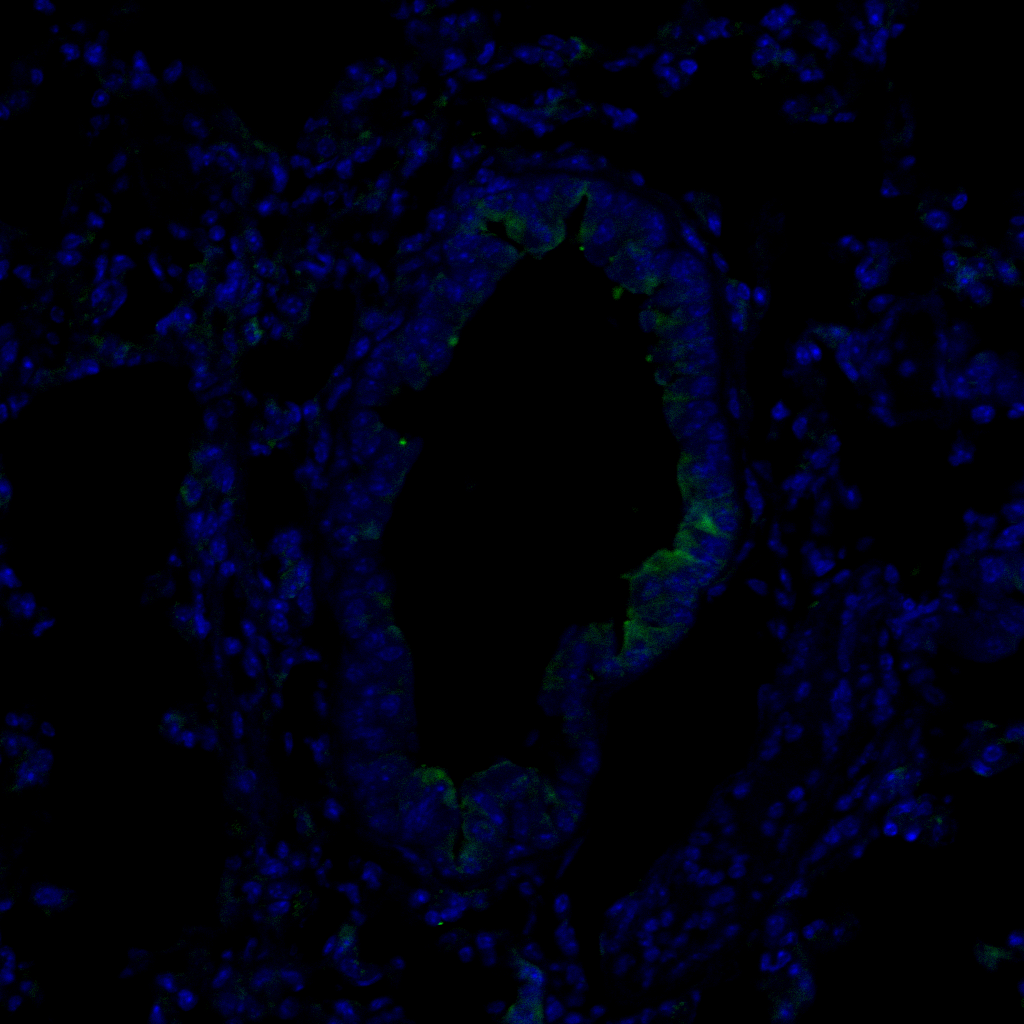

Supplement: Supplementary file 1 [file Data_Sheet_1.ZIP › Immunofluorescence/p-p38/OVA+DEX/1.tif]

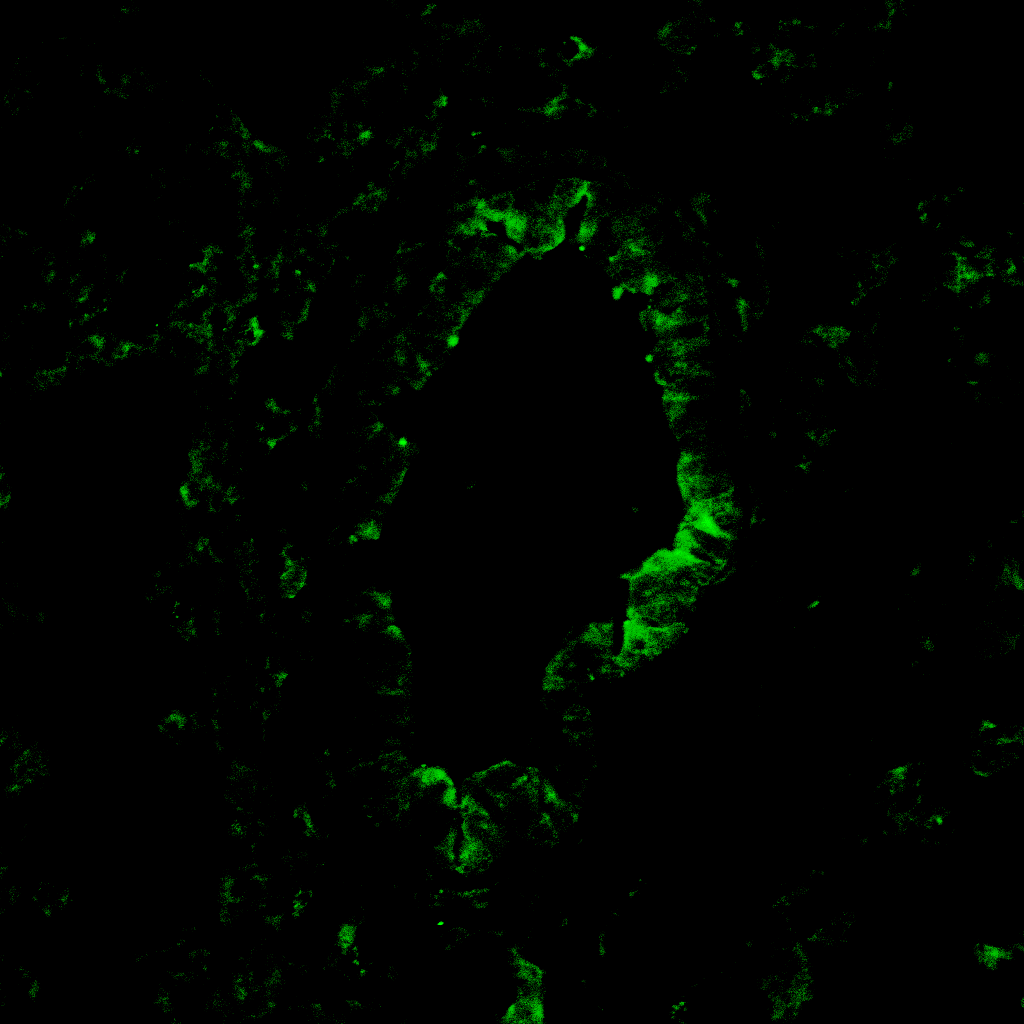

Supplement: Supplementary file 1 [file Data_Sheet_1.ZIP › Immunofluorescence/p-p38/OVA+DEX/2.tif]

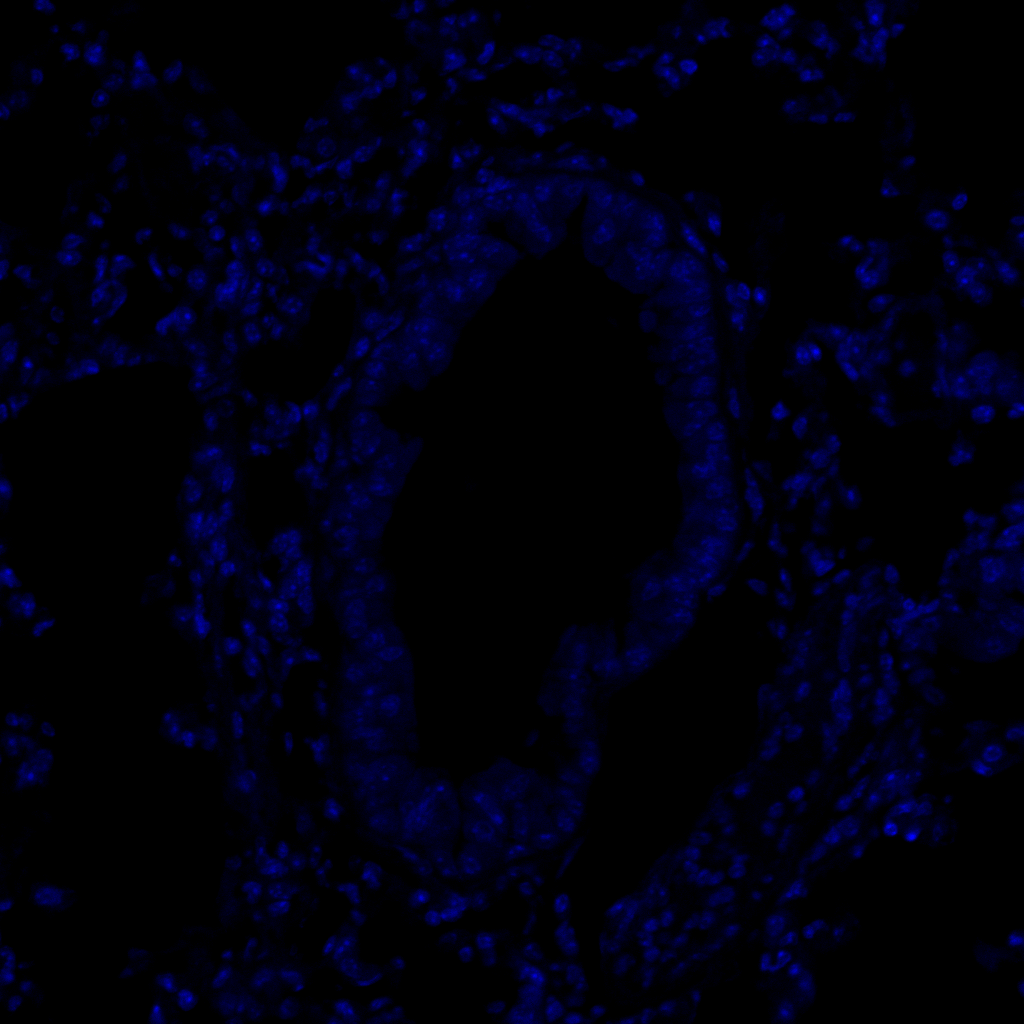

Supplement: Supplementary file 1 [file Data_Sheet_1.ZIP › Immunofluorescence/p-p38/OVA+DEX/3.tif]

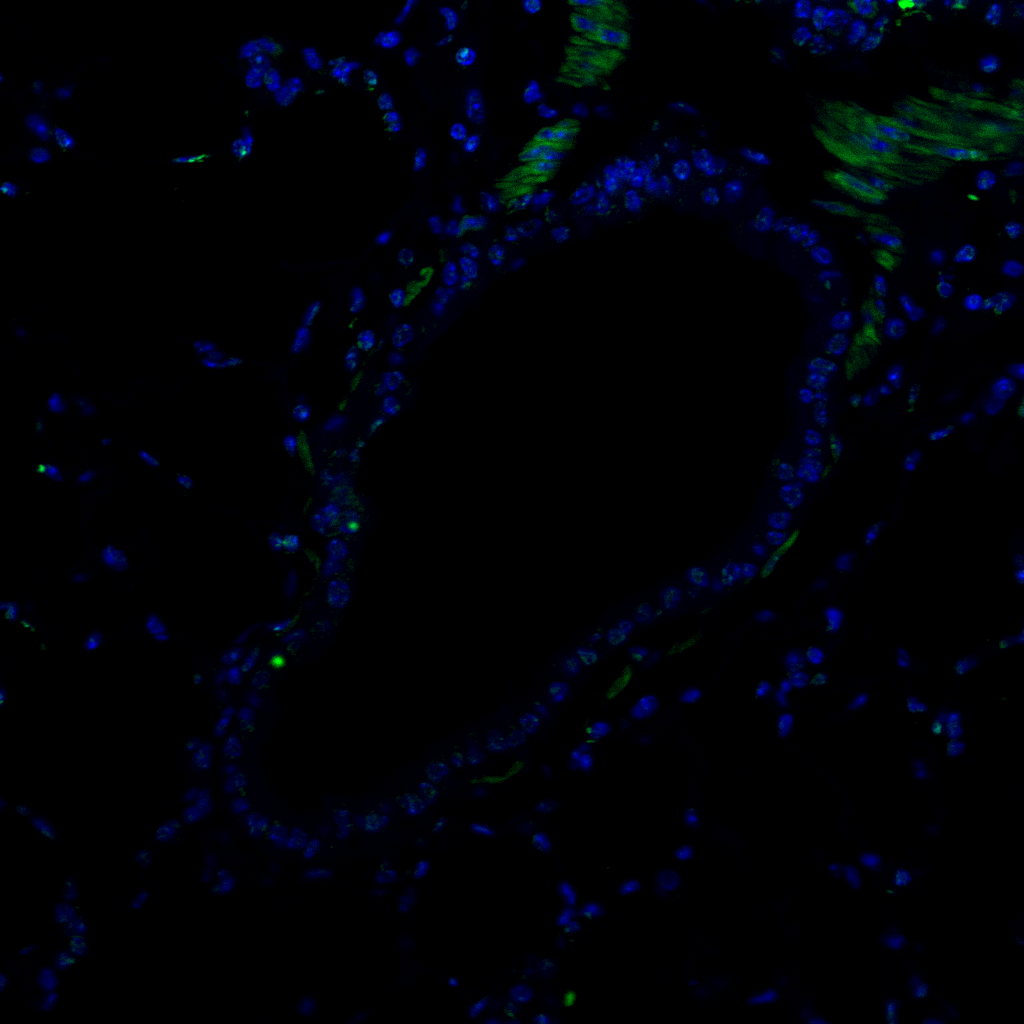

Supplement: Supplementary file 1 [file Data_Sheet_1.ZIP › Immunofluorescence/P65/control/1.tif]

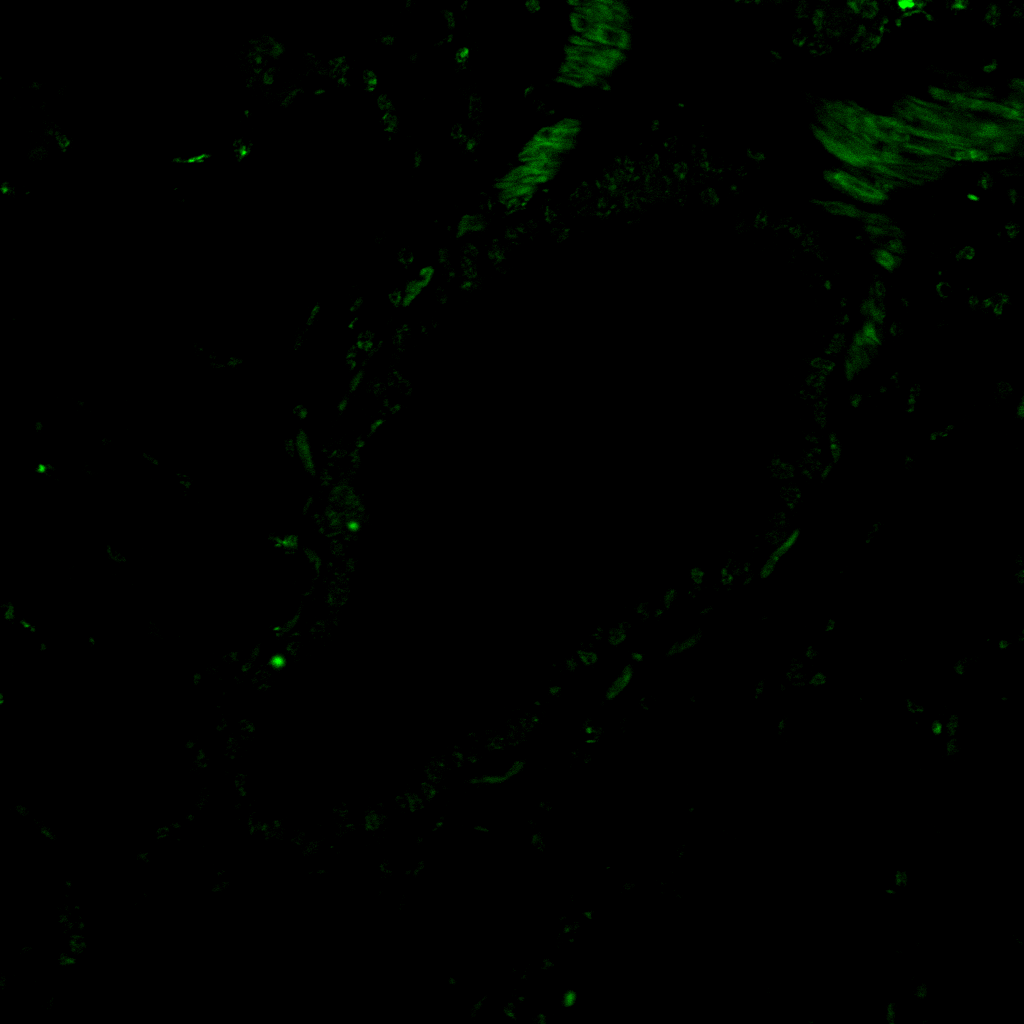

Supplement: Supplementary file 1 [file Data_Sheet_1.ZIP › Immunofluorescence/P65/control/2.tif]

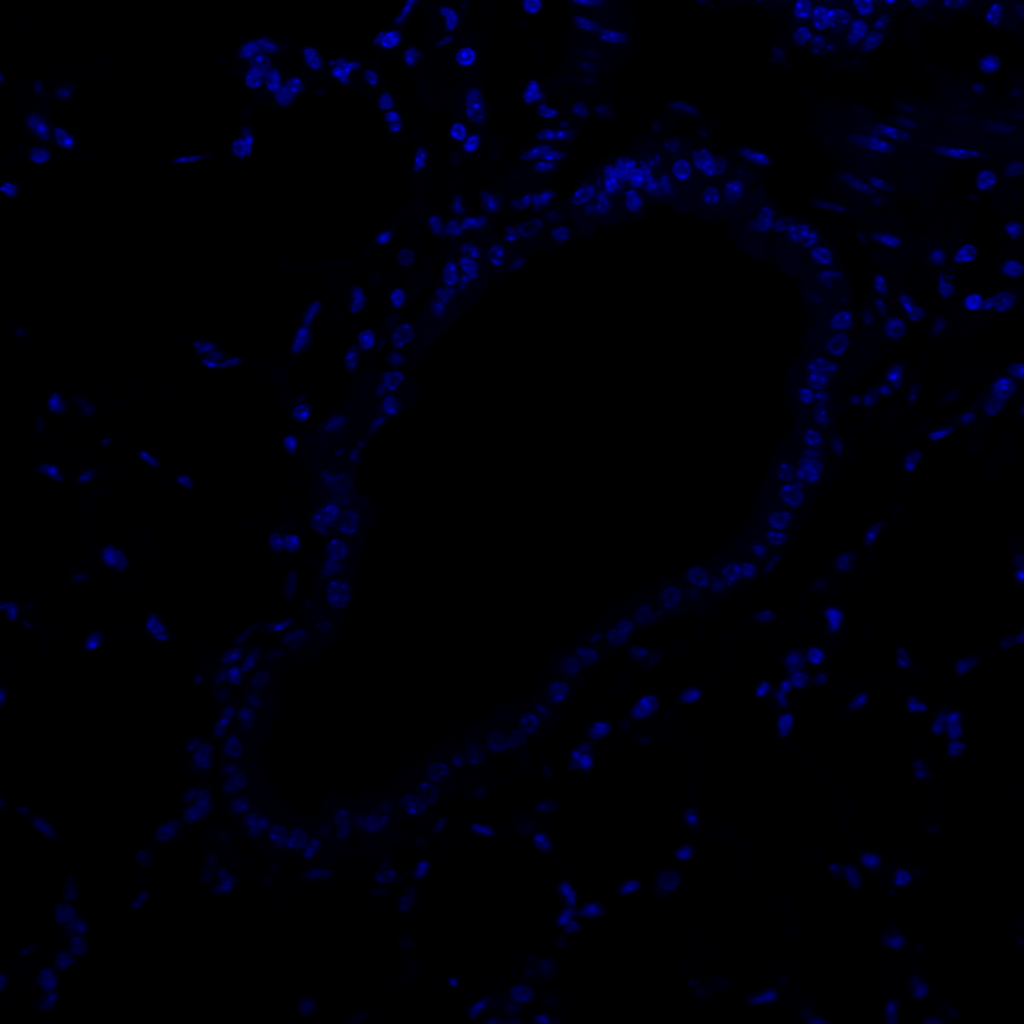

Supplement: Supplementary file 1 [file Data_Sheet_1.ZIP › Immunofluorescence/P65/control/3.tif]

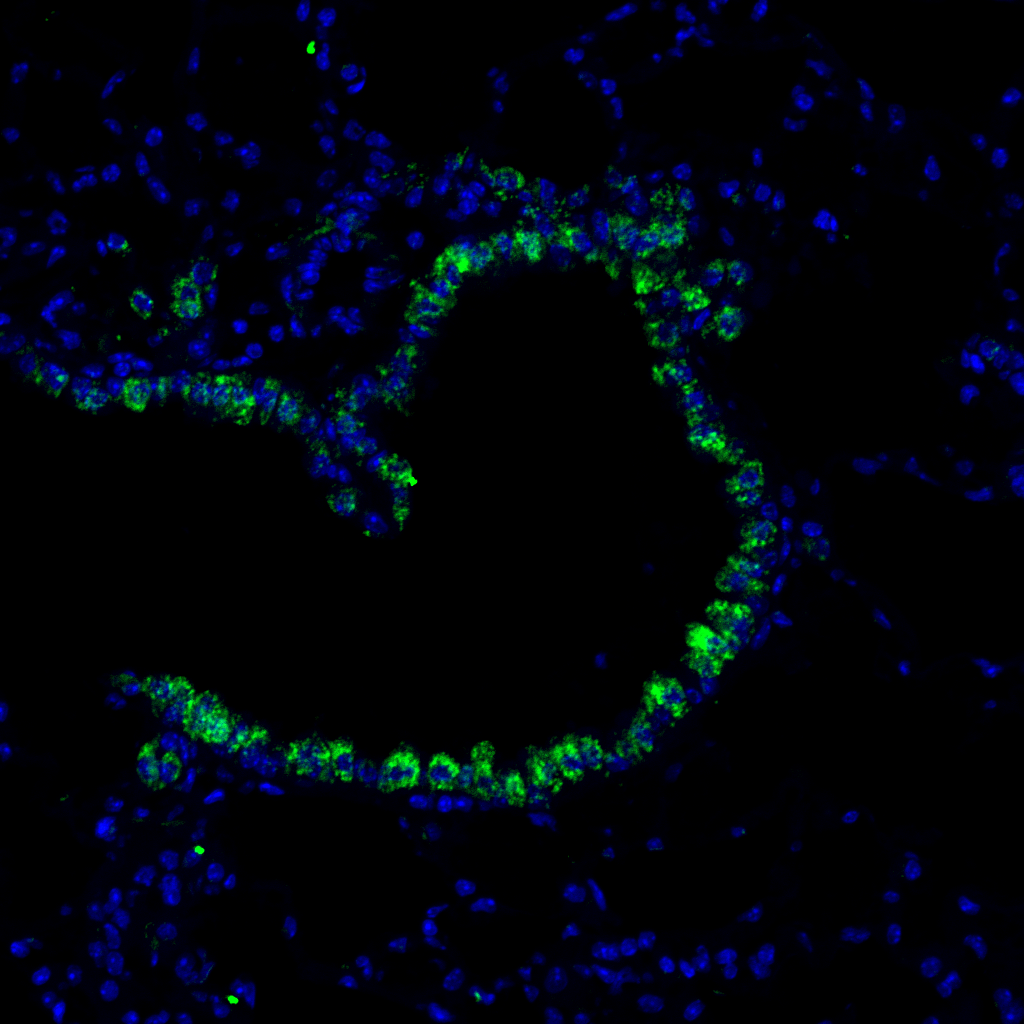

Supplement: Supplementary file 1 [file Data_Sheet_1.ZIP › Immunofluorescence/P65/OVA/1.tif]

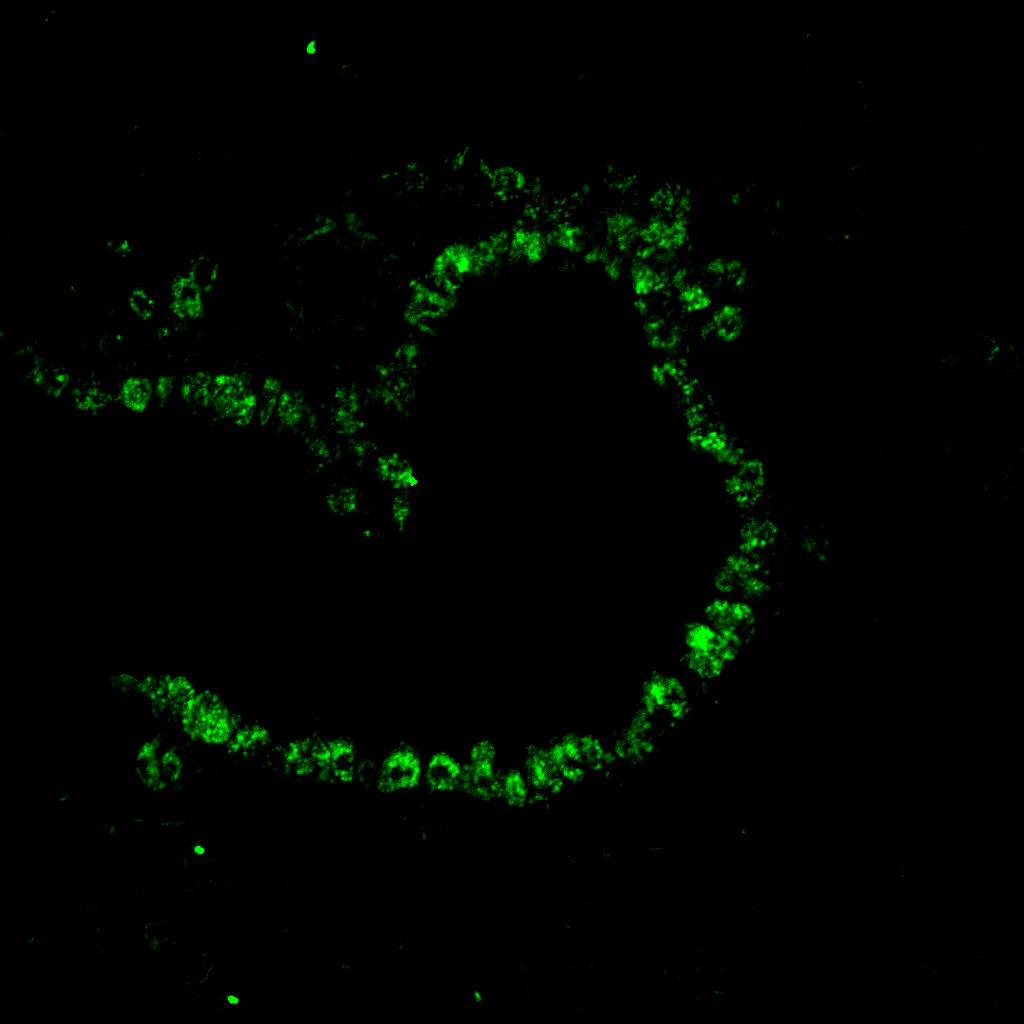

Supplement: Supplementary file 1 [file Data_Sheet_1.ZIP › Immunofluorescence/P65/OVA/2.tif]

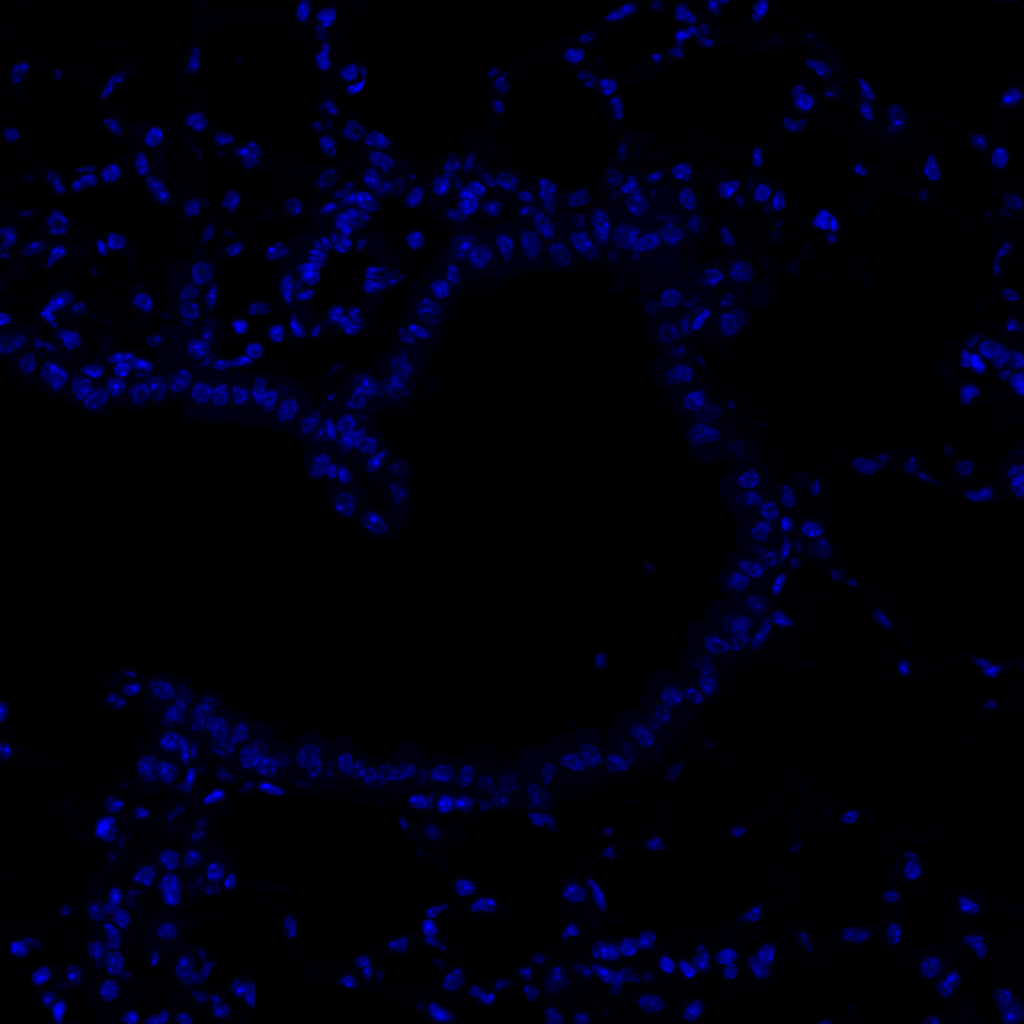

Supplement: Supplementary file 1 [file Data_Sheet_1.ZIP › Immunofluorescence/P65/OVA/3.tif]

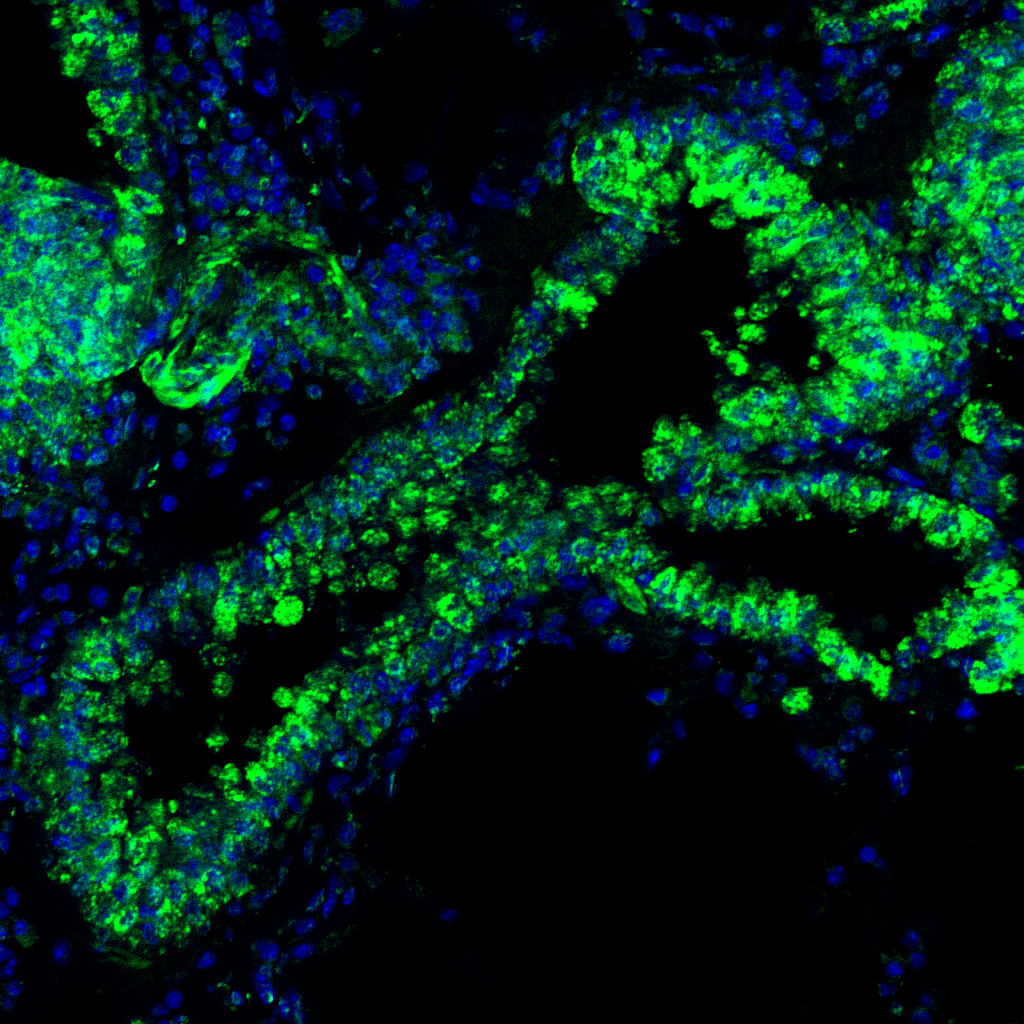

Supplement: Supplementary file 1 [file Data_Sheet_1.ZIP › Immunofluorescence/P65/OVA+CIH/1.tif]

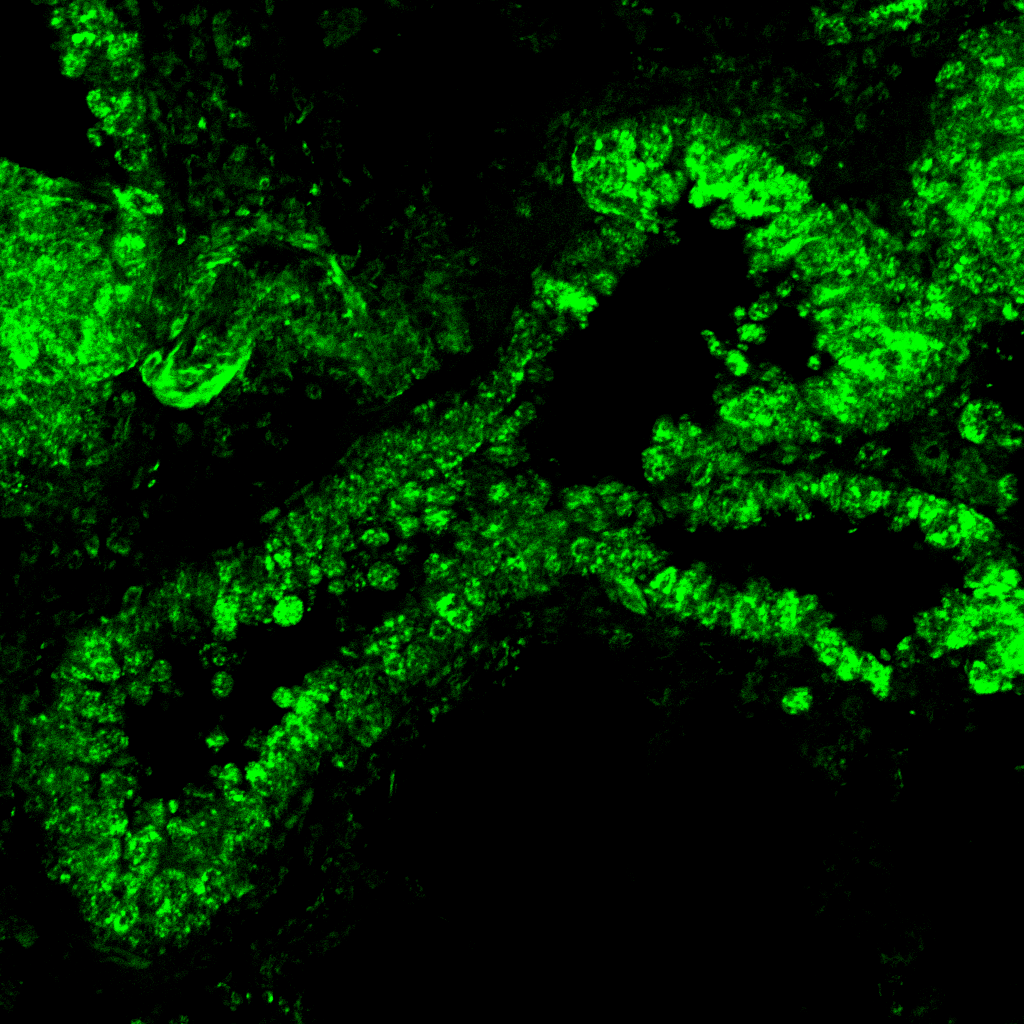

Supplement: Supplementary file 1 [file Data_Sheet_1.ZIP › Immunofluorescence/P65/OVA+CIH/2.tif]

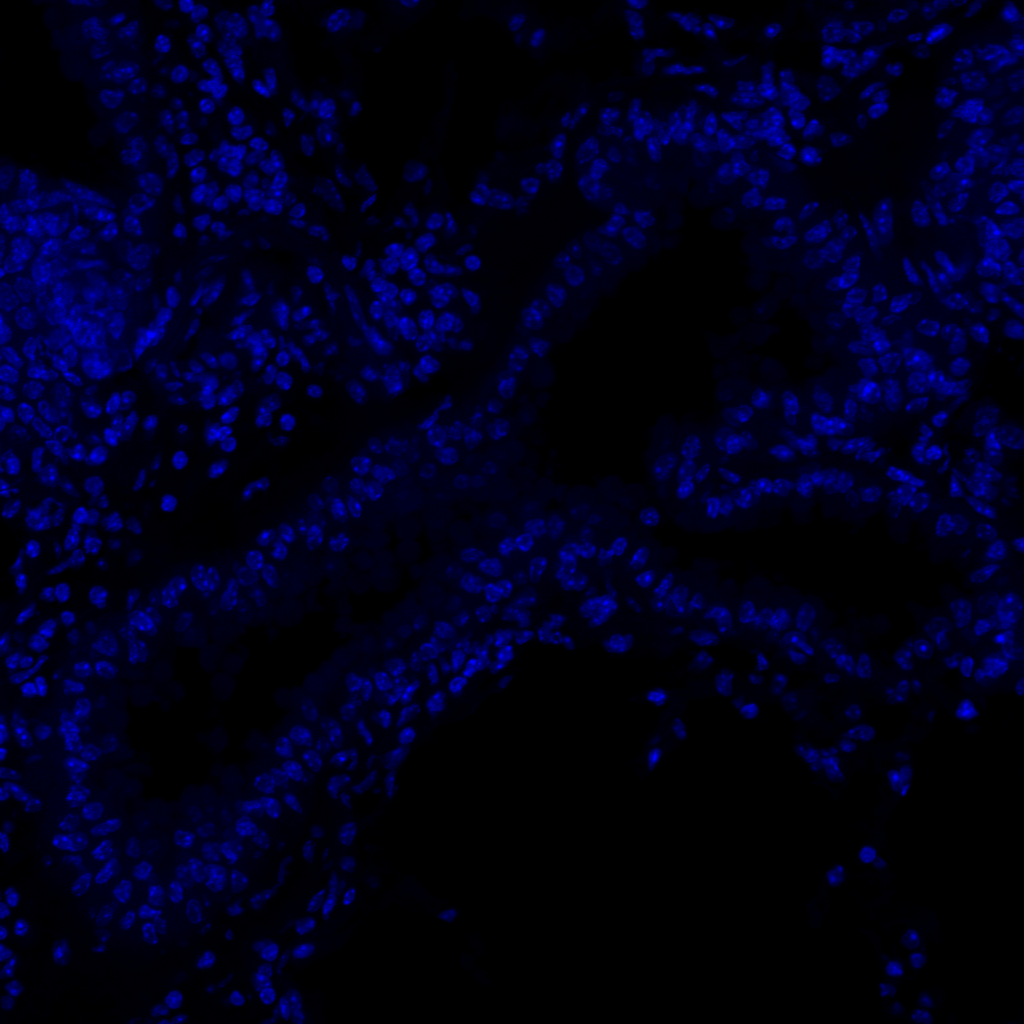

Supplement: Supplementary file 1 [file Data_Sheet_1.ZIP › Immunofluorescence/P65/OVA+CIH/3.tif]

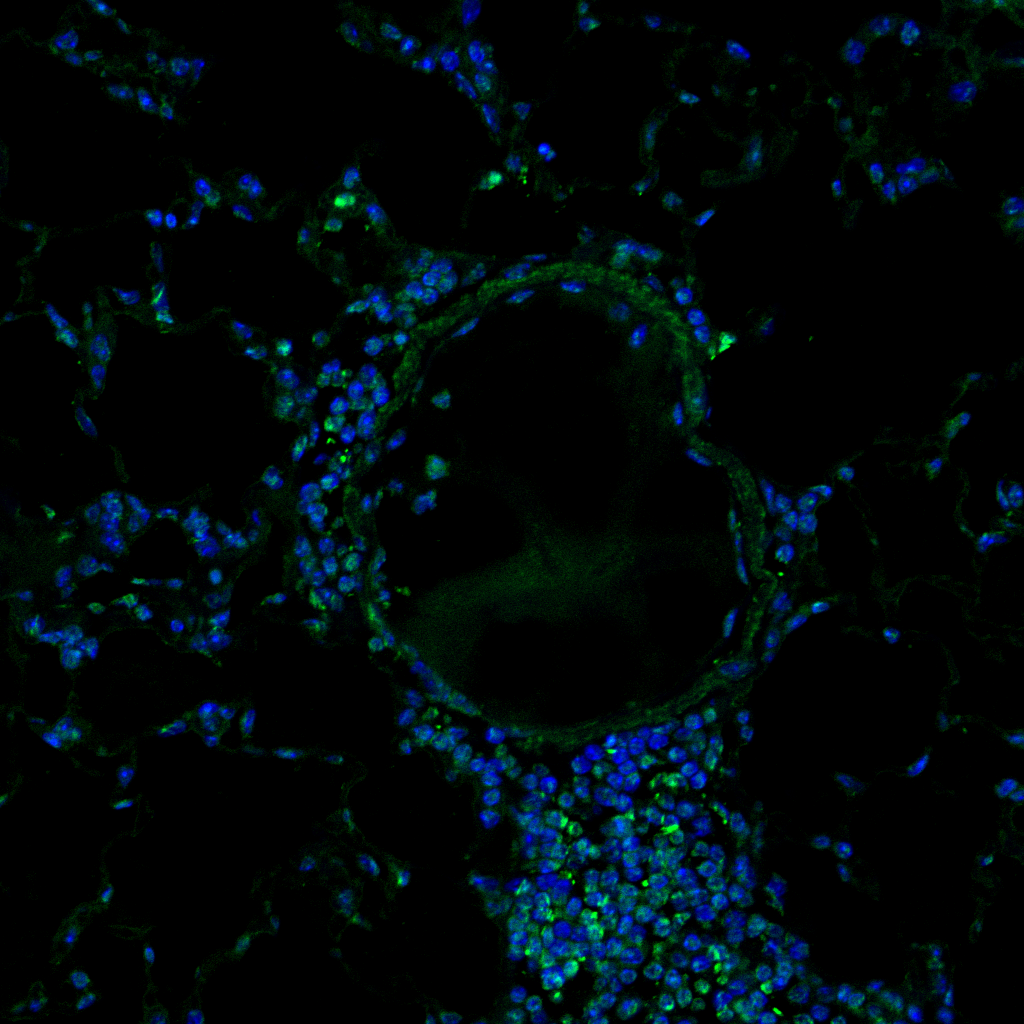

Supplement: Supplementary file 1 [file Data_Sheet_1.ZIP › Immunofluorescence/P65/OVA+CIH+DEX/1.tif]

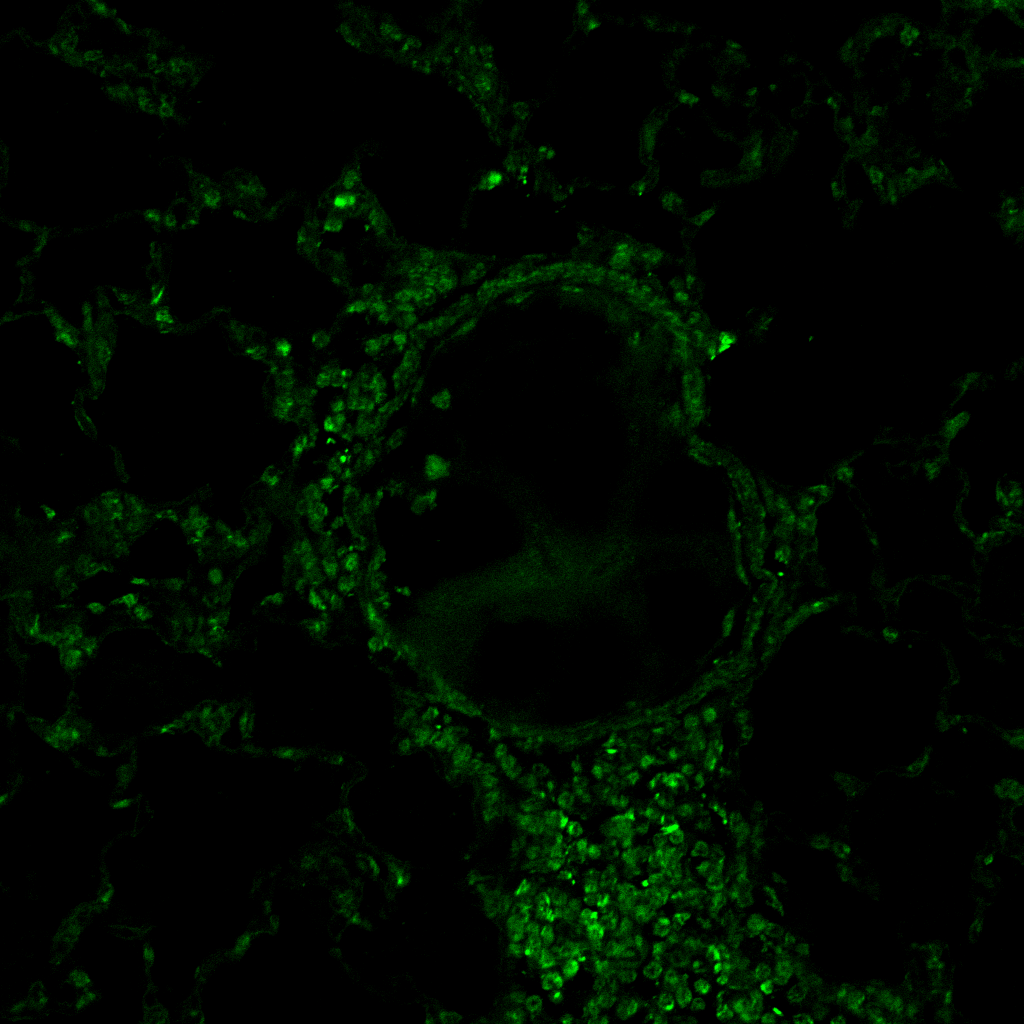

Supplement: Supplementary file 1 [file Data_Sheet_1.ZIP › Immunofluorescence/P65/OVA+CIH+DEX/2.tif]

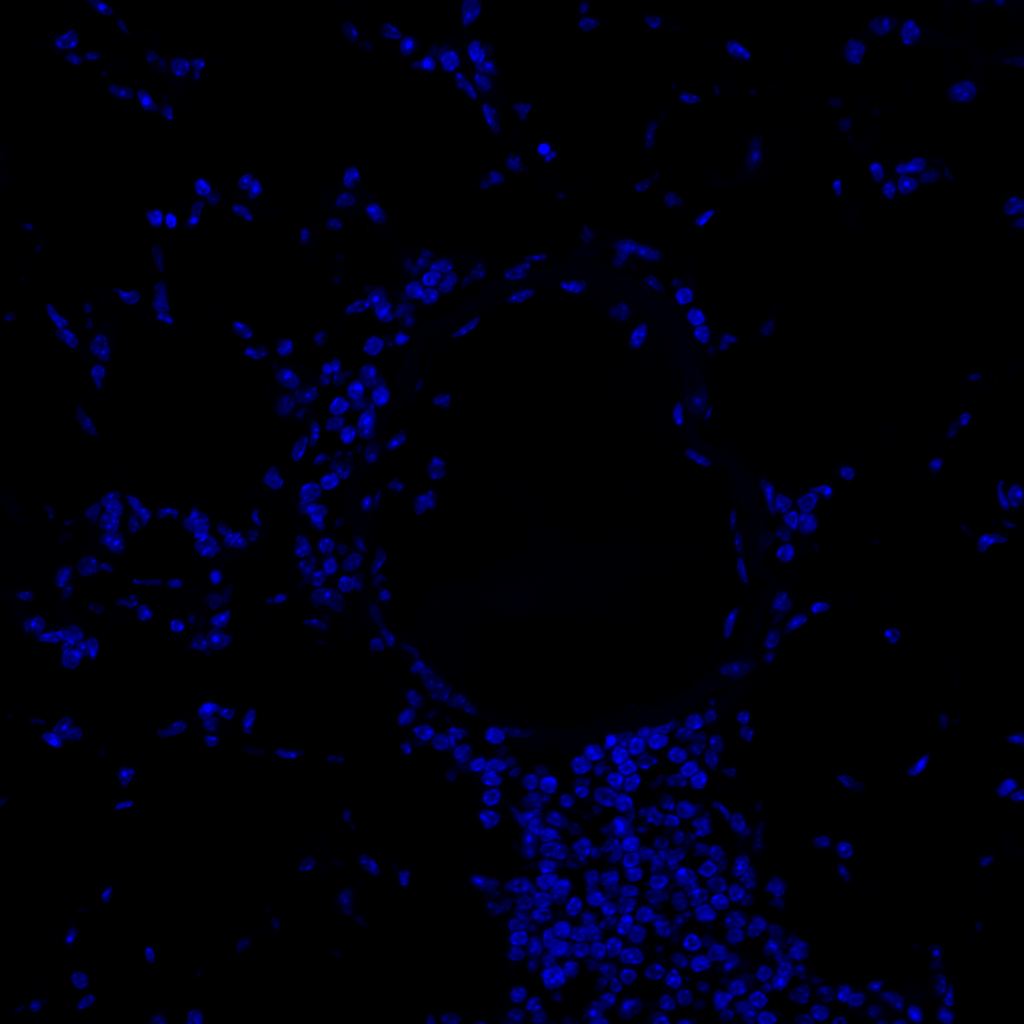

Supplement: Supplementary file 1 [file Data_Sheet_1.ZIP › Immunofluorescence/P65/OVA+CIH+DEX/3.tif]

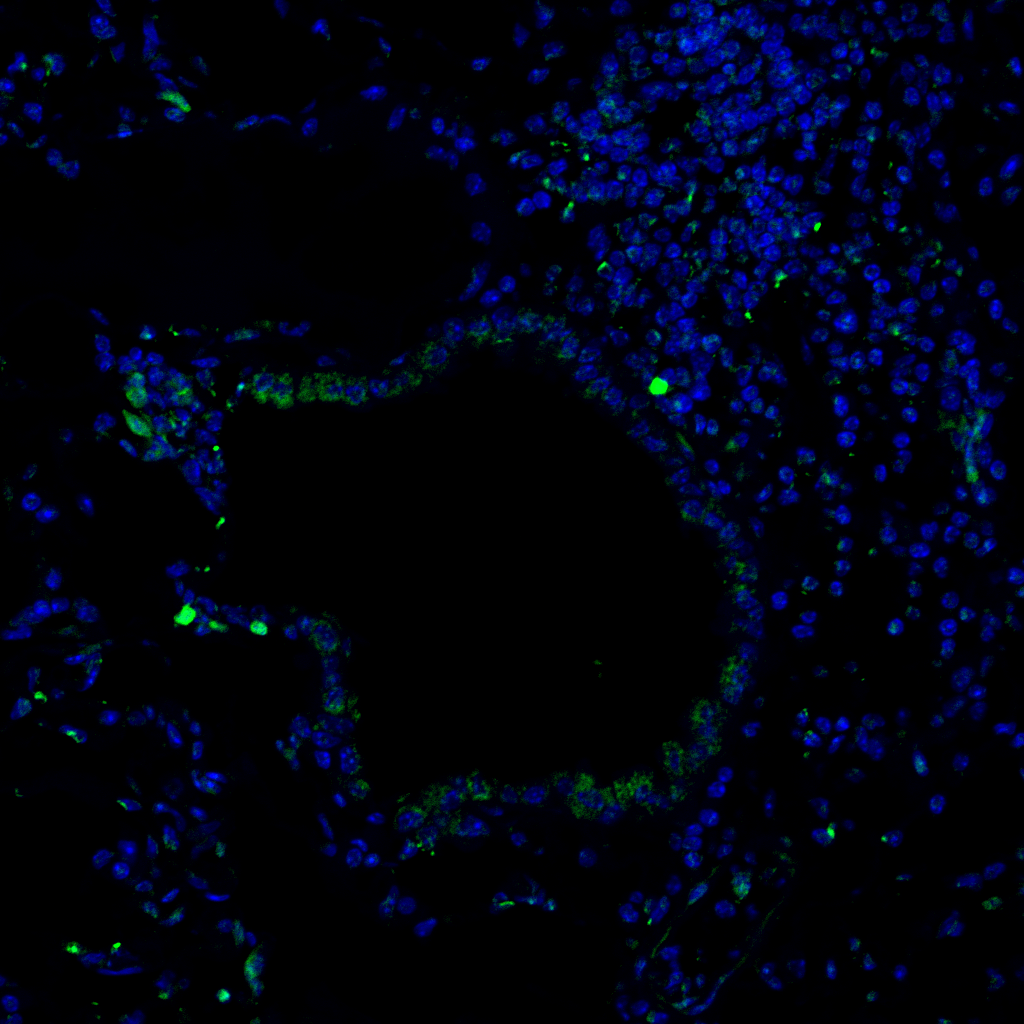

Supplement: Supplementary file 1 [file Data_Sheet_1.ZIP › Immunofluorescence/P65/OVA+CIH+DEX+SB/1.tif]

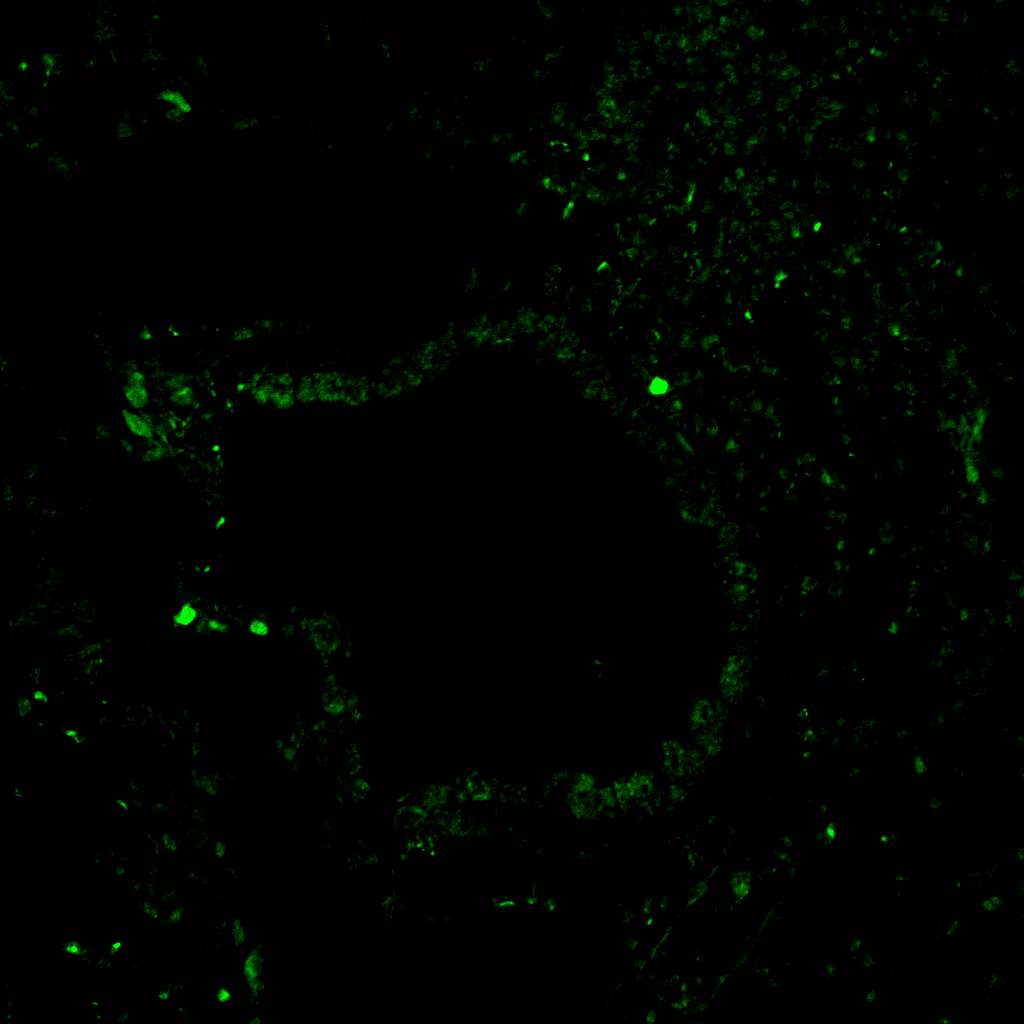

Supplement: Supplementary file 1 [file Data_Sheet_1.ZIP › Immunofluorescence/P65/OVA+CIH+DEX+SB/2.tif]

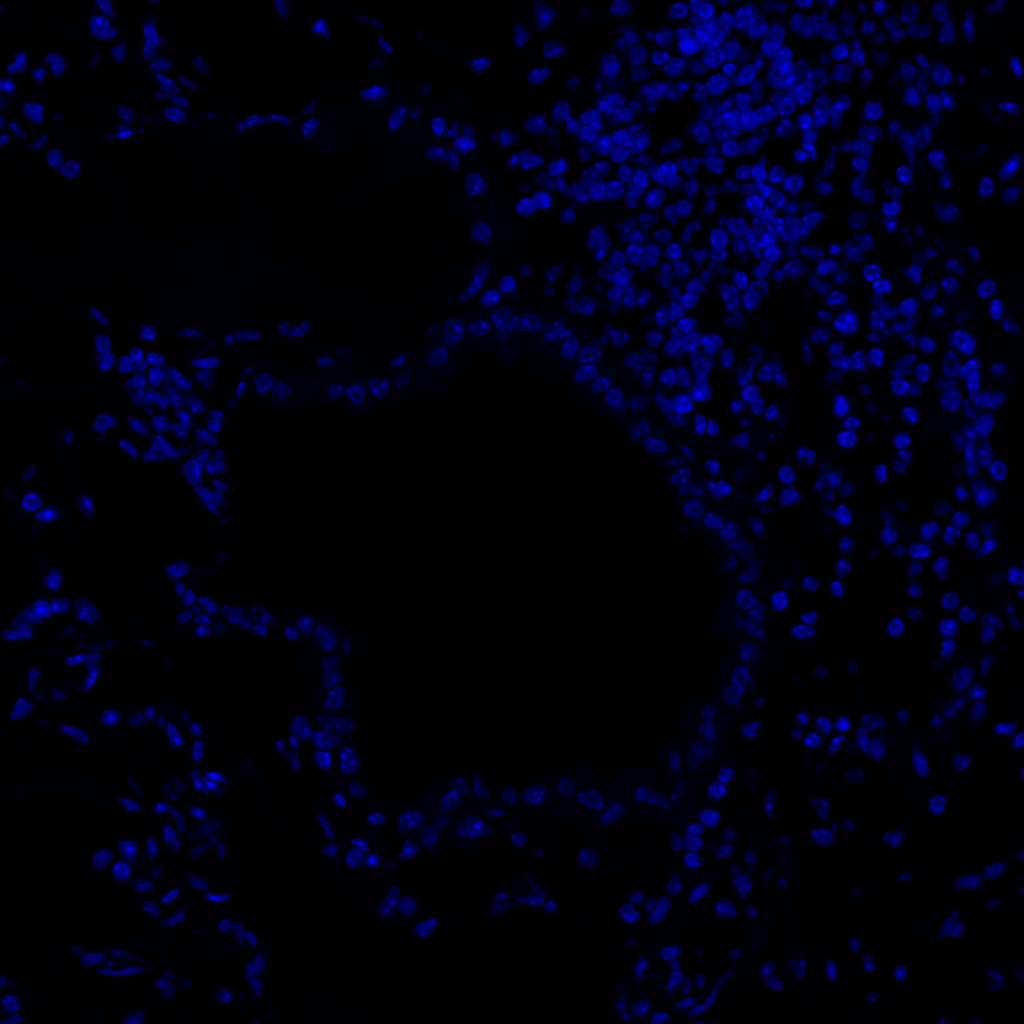

Supplement: Supplementary file 1 [file Data_Sheet_1.ZIP › Immunofluorescence/P65/OVA+CIH+DEX+SB/3.tif]

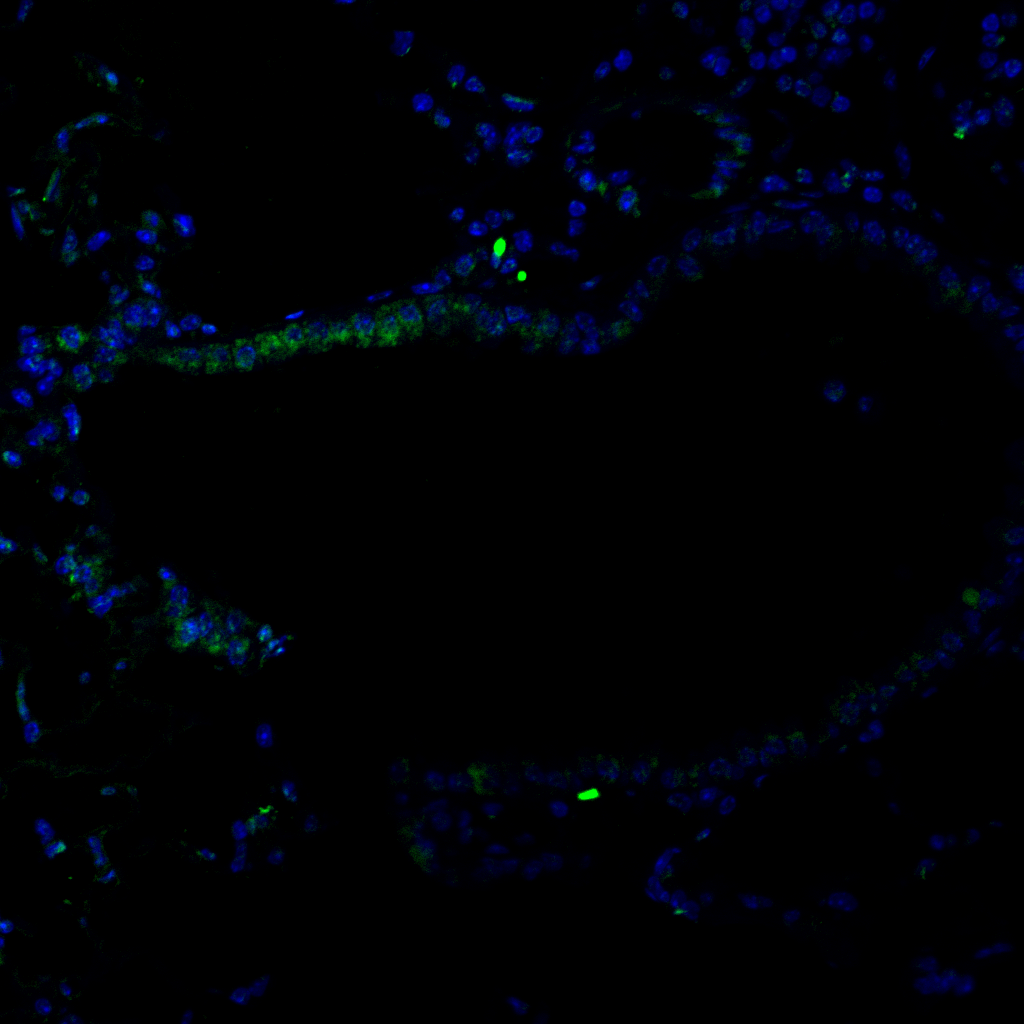

Supplement: Supplementary file 1 [file Data_Sheet_1.ZIP › Immunofluorescence/P65/OVA+DEX/1.tif]

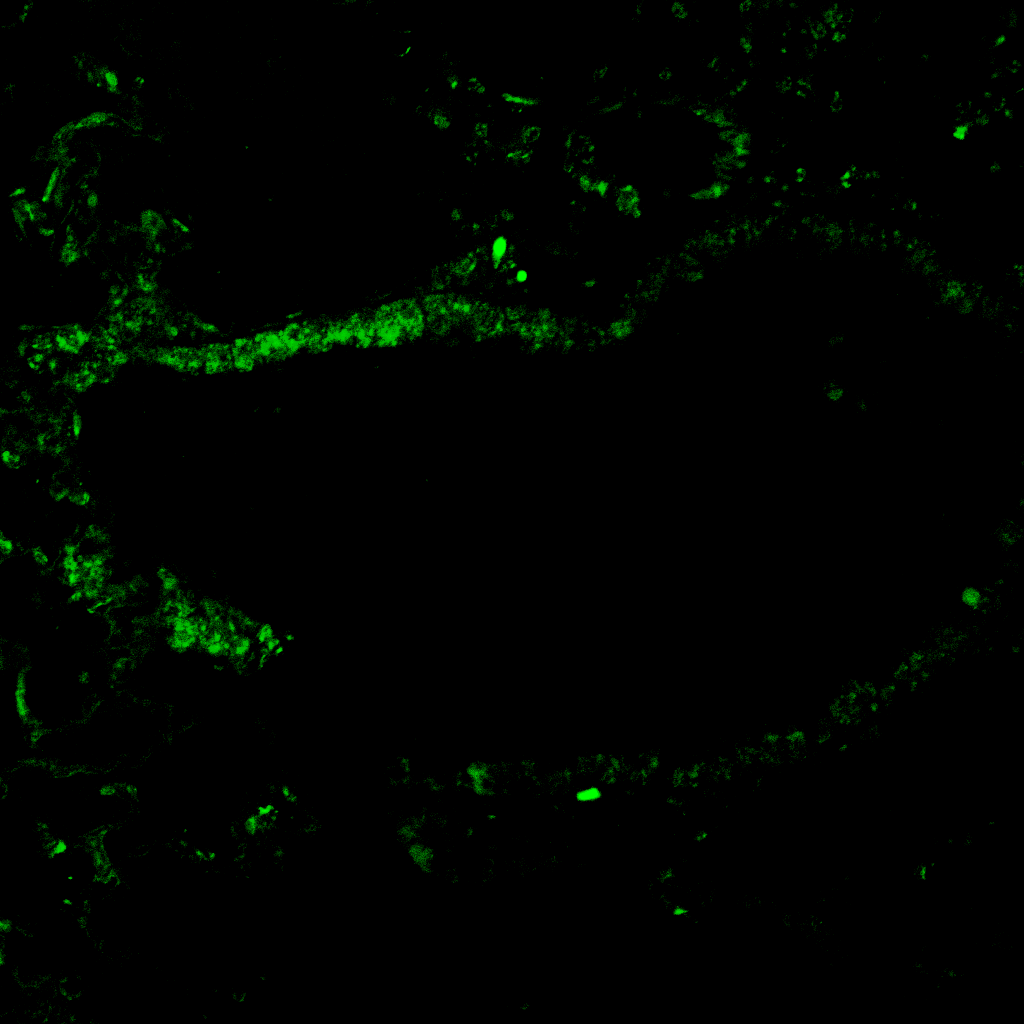

Supplement: Supplementary file 1 [file Data_Sheet_1.ZIP › Immunofluorescence/P65/OVA+DEX/2.tif]

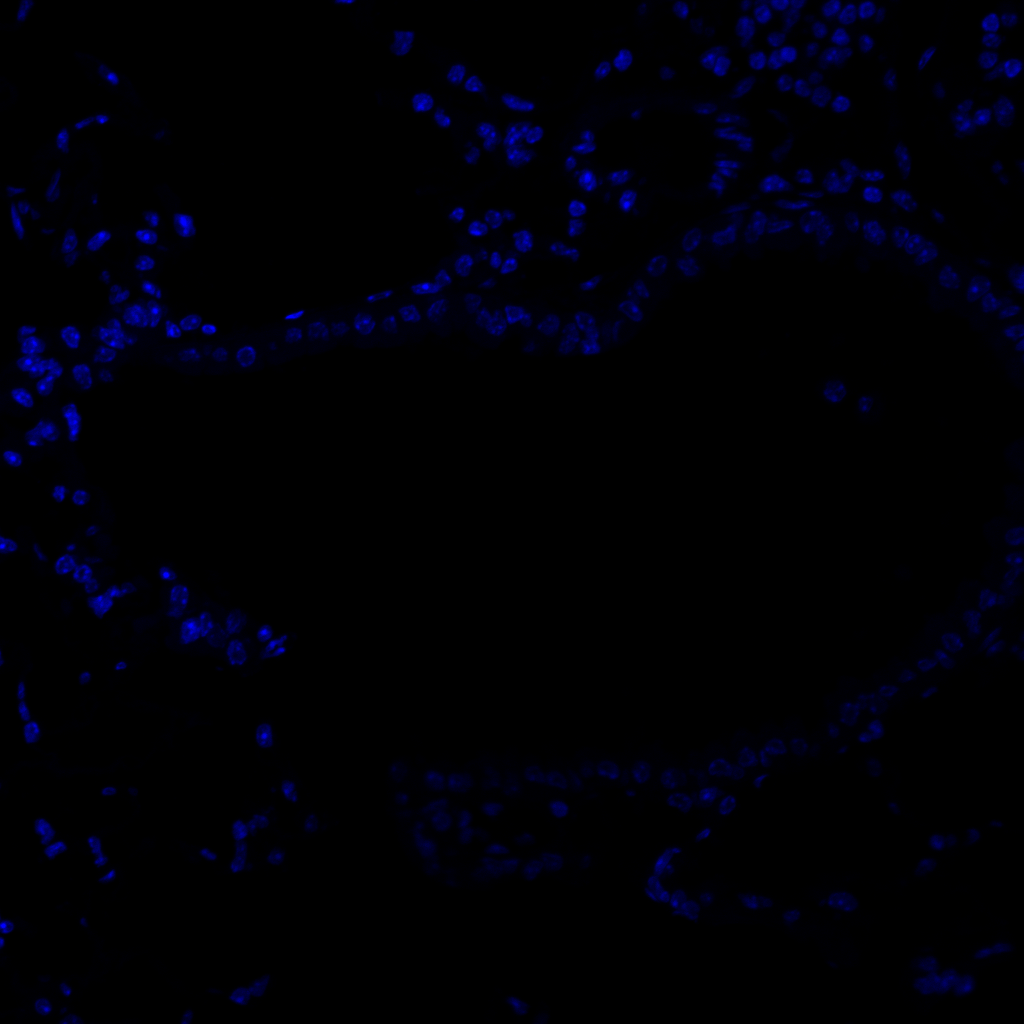

Supplement: Supplementary file 1 [file Data_Sheet_1.ZIP › Immunofluorescence/P65/OVA+DEX/3.tif]

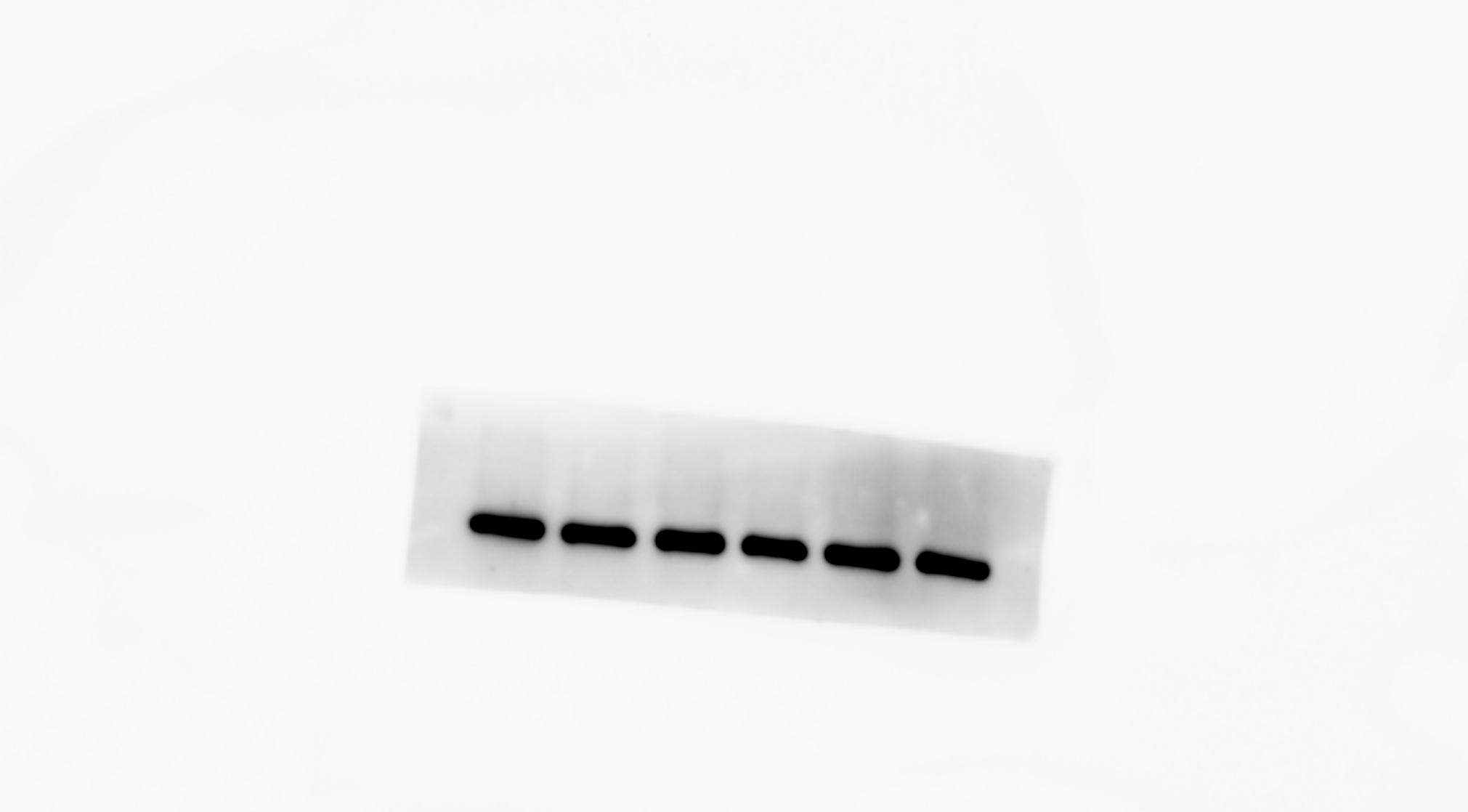

Supplement: Supplementary file 2 [file Data_Sheet_2.ZIP › WB/Figure 3/actin/1.tif]

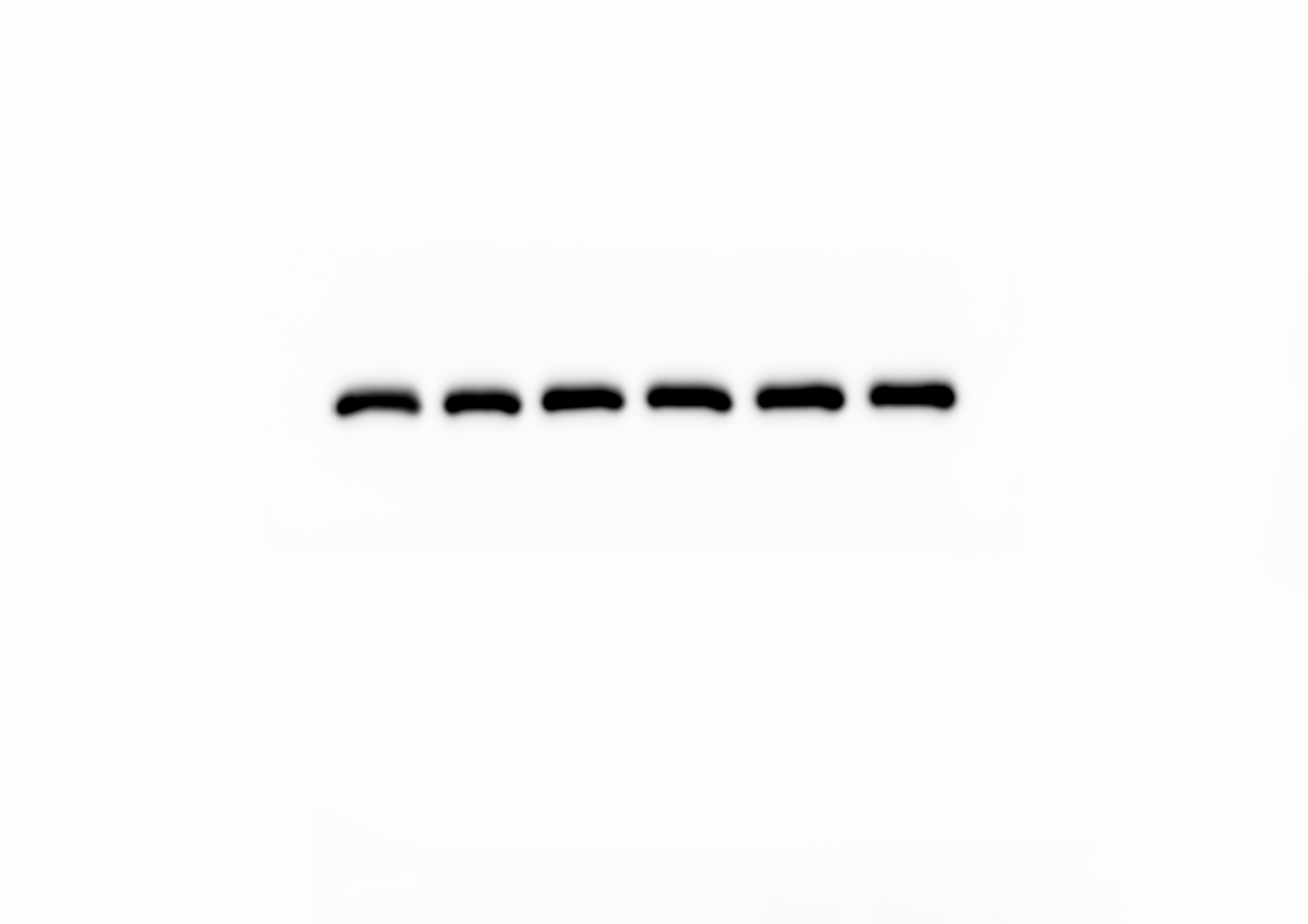

Supplement: Supplementary file 2 [file Data_Sheet_2.ZIP › WB/Figure 3/actin/2.tif]

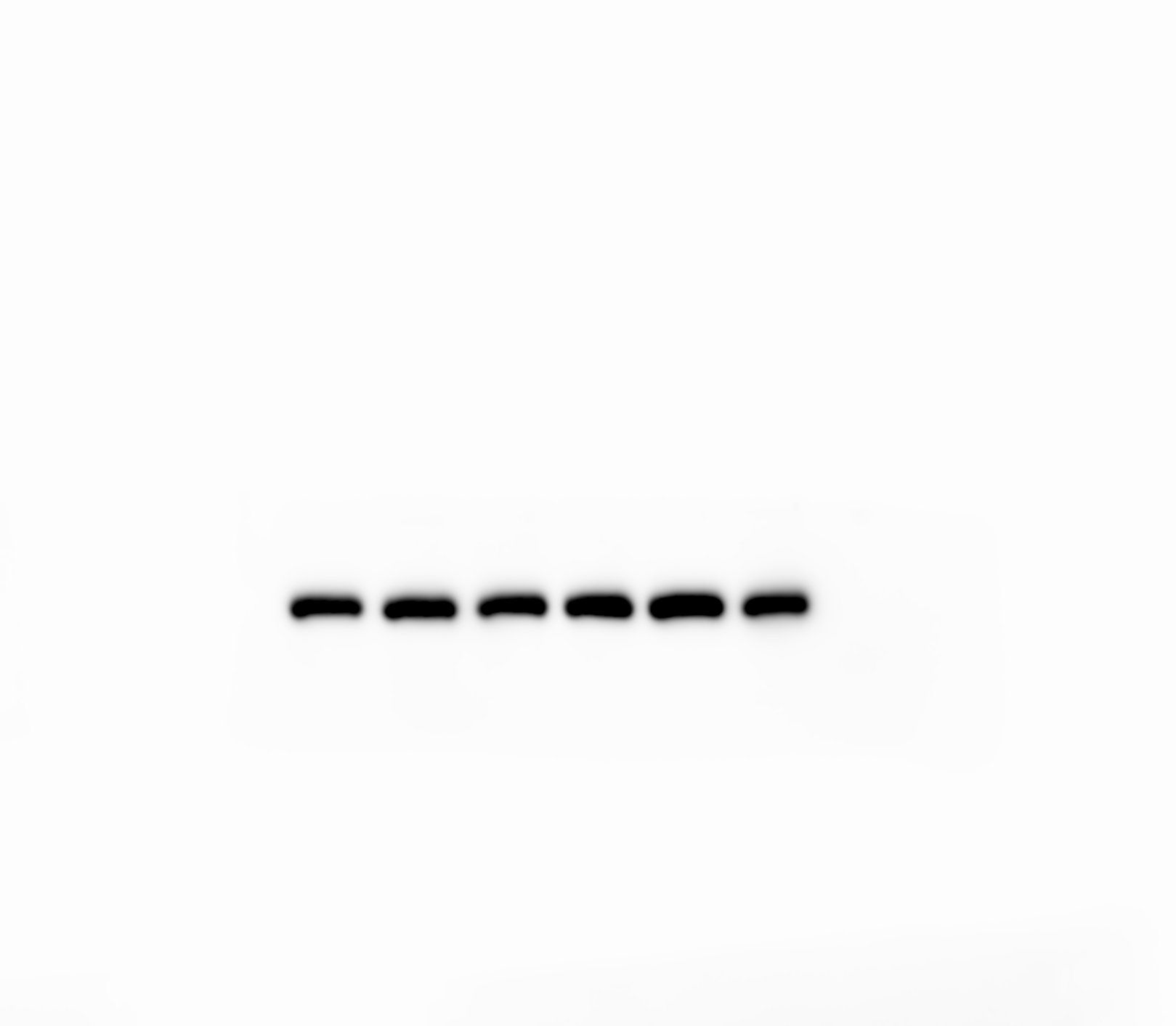

Supplement: Supplementary file 2 [file Data_Sheet_2.ZIP › WB/Figure 3/actin/3.tif]

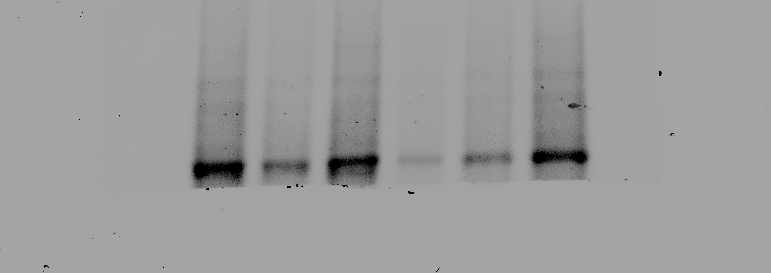

Supplement: Supplementary file 2 [file Data_Sheet_2.ZIP › WB/Figure 3/HO-1/1.tif]

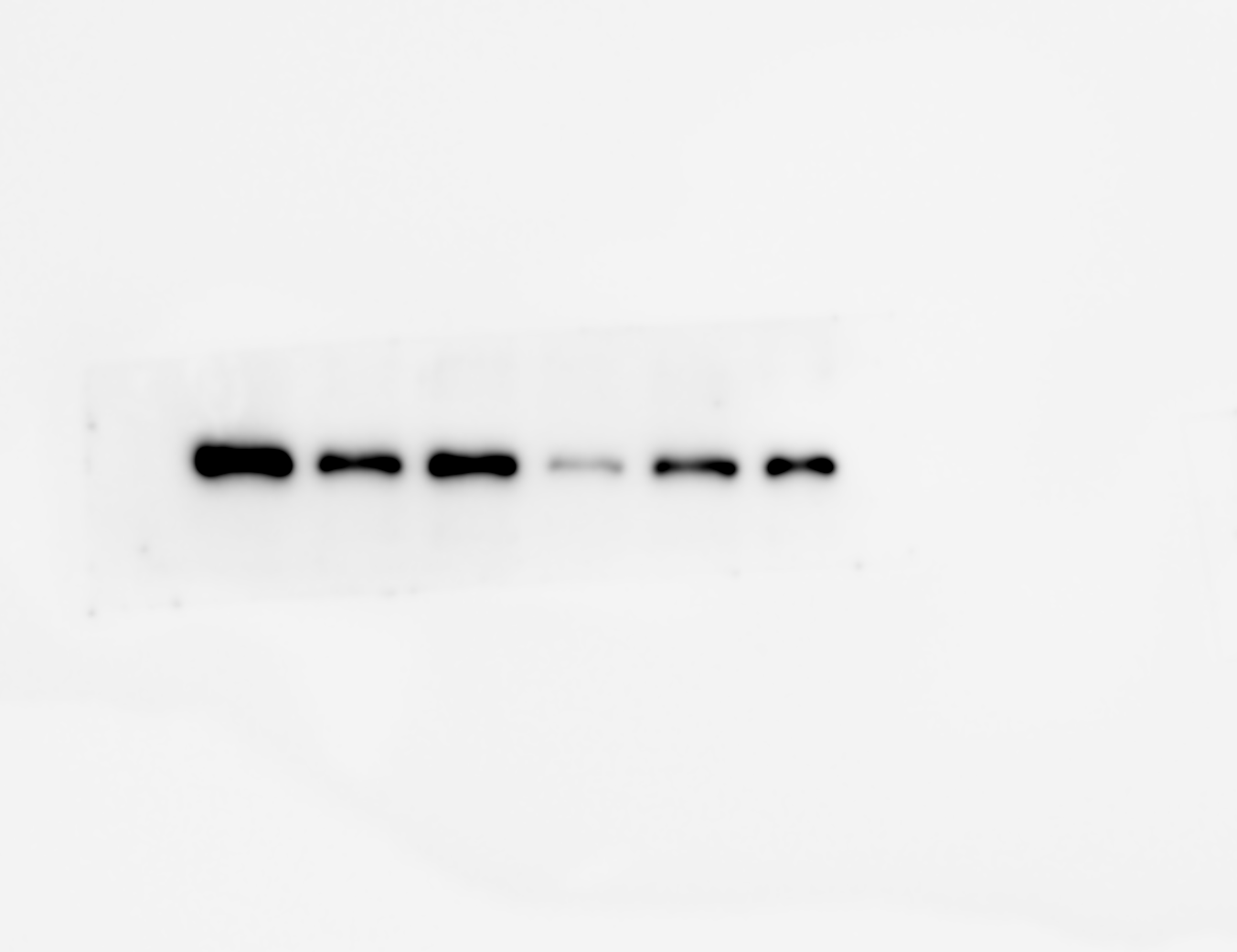

Supplement: Supplementary file 2 [file Data_Sheet_2.ZIP › WB/Figure 3/HO-1/2.tif]

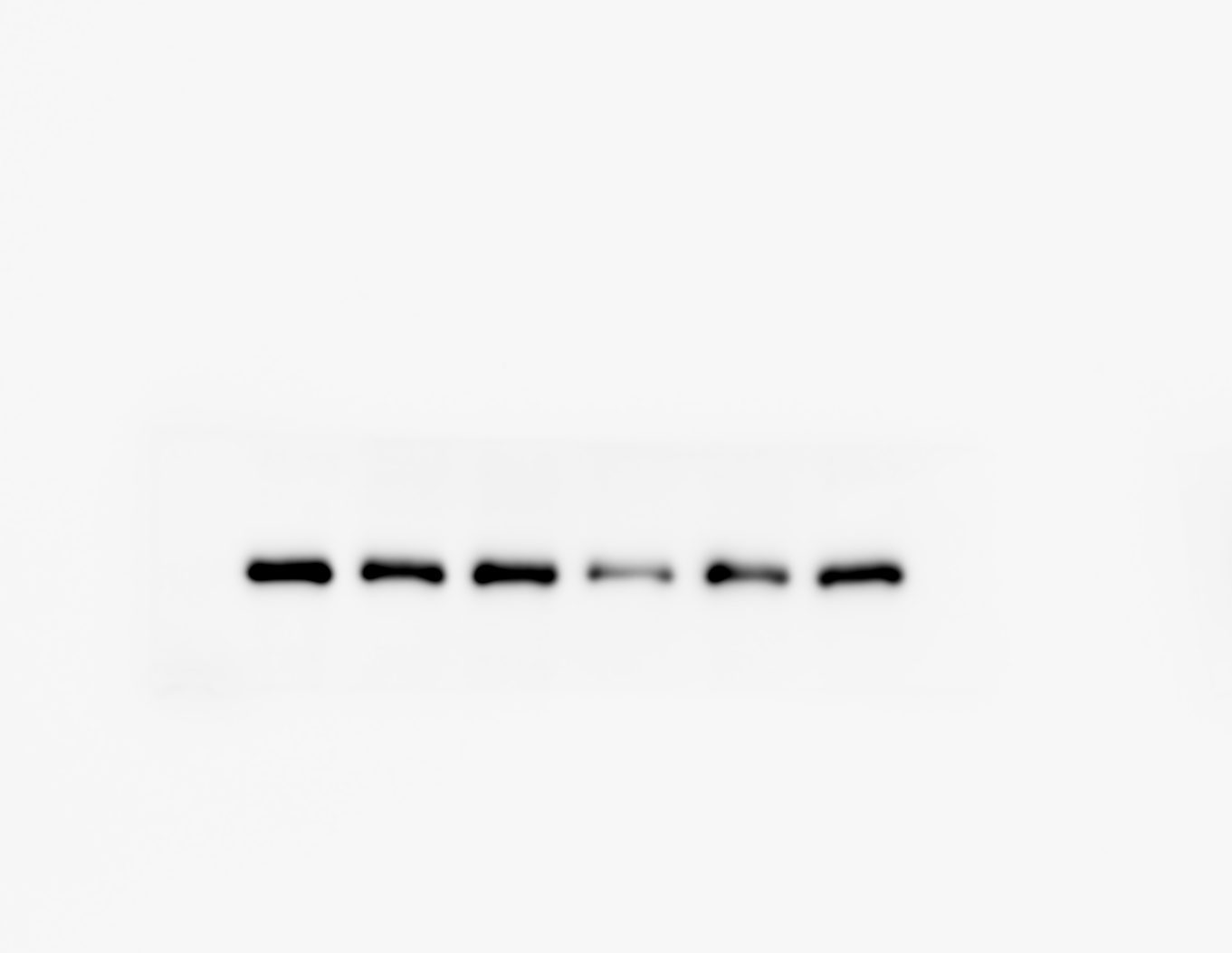

Supplement: Supplementary file 2 [file Data_Sheet_2.ZIP › WB/Figure 3/HO-1/3.tif]

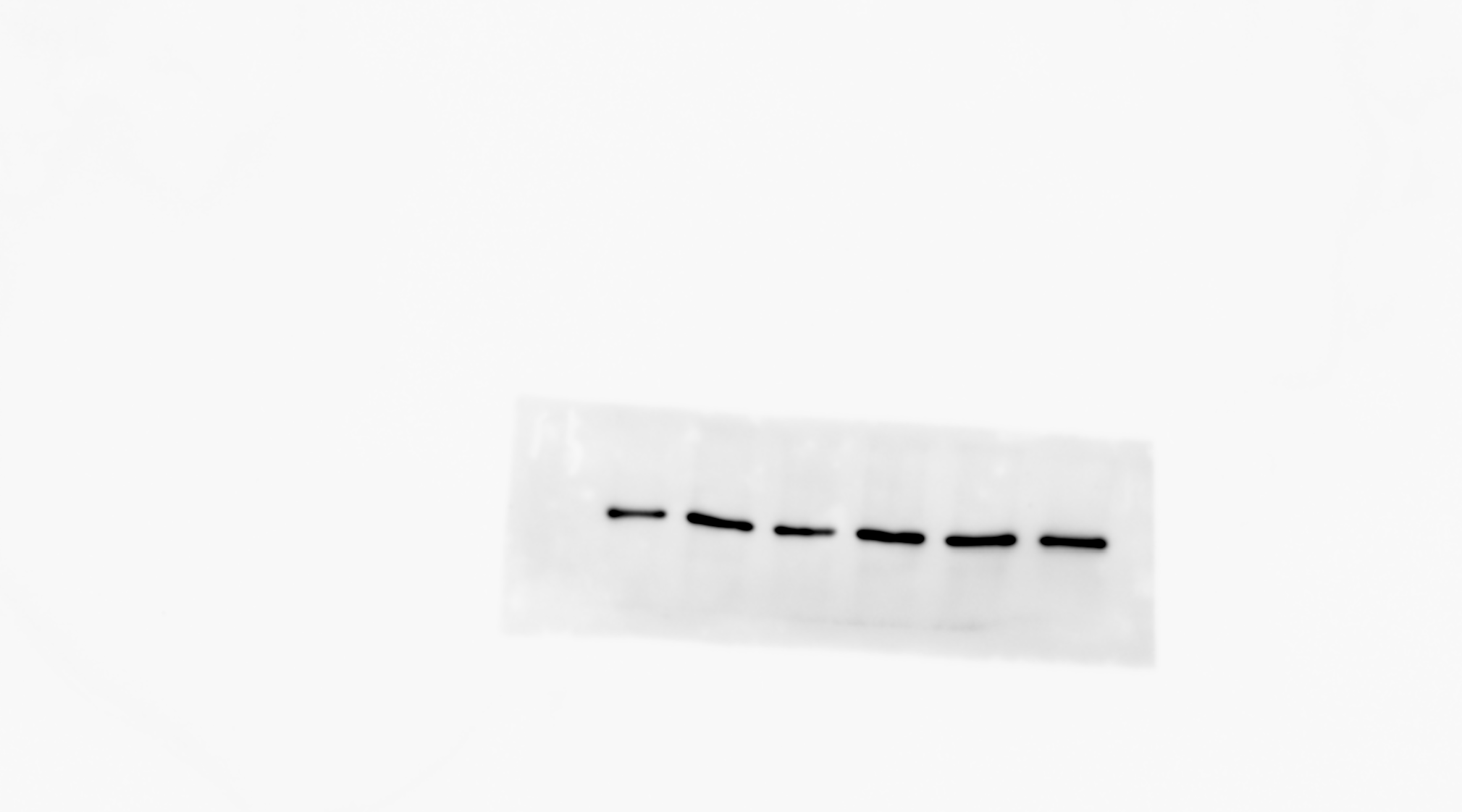

Supplement: Supplementary file 2 [file Data_Sheet_2.ZIP › WB/Figure 3/laminb/1.tif]

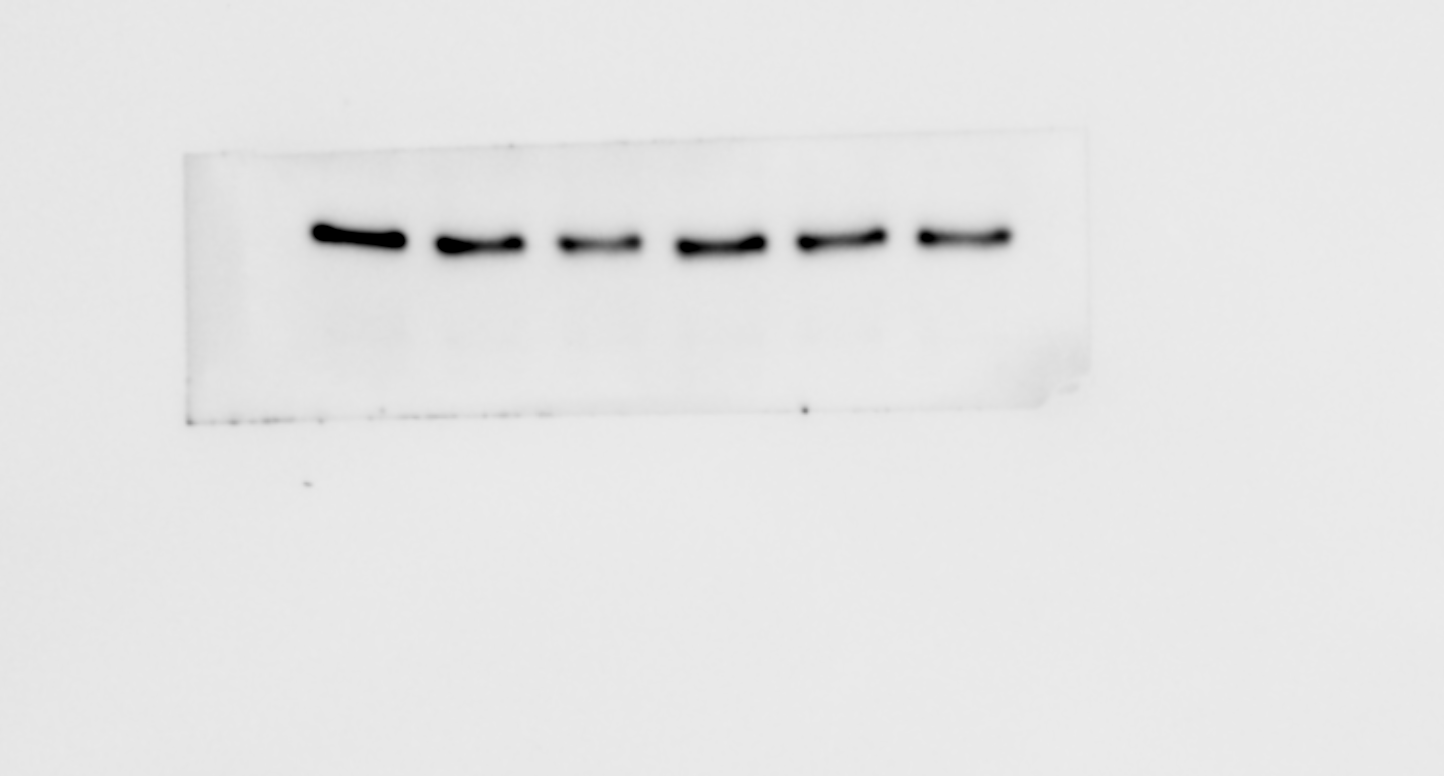

Supplement: Supplementary file 2 [file Data_Sheet_2.ZIP › WB/Figure 3/laminb/2.tif]

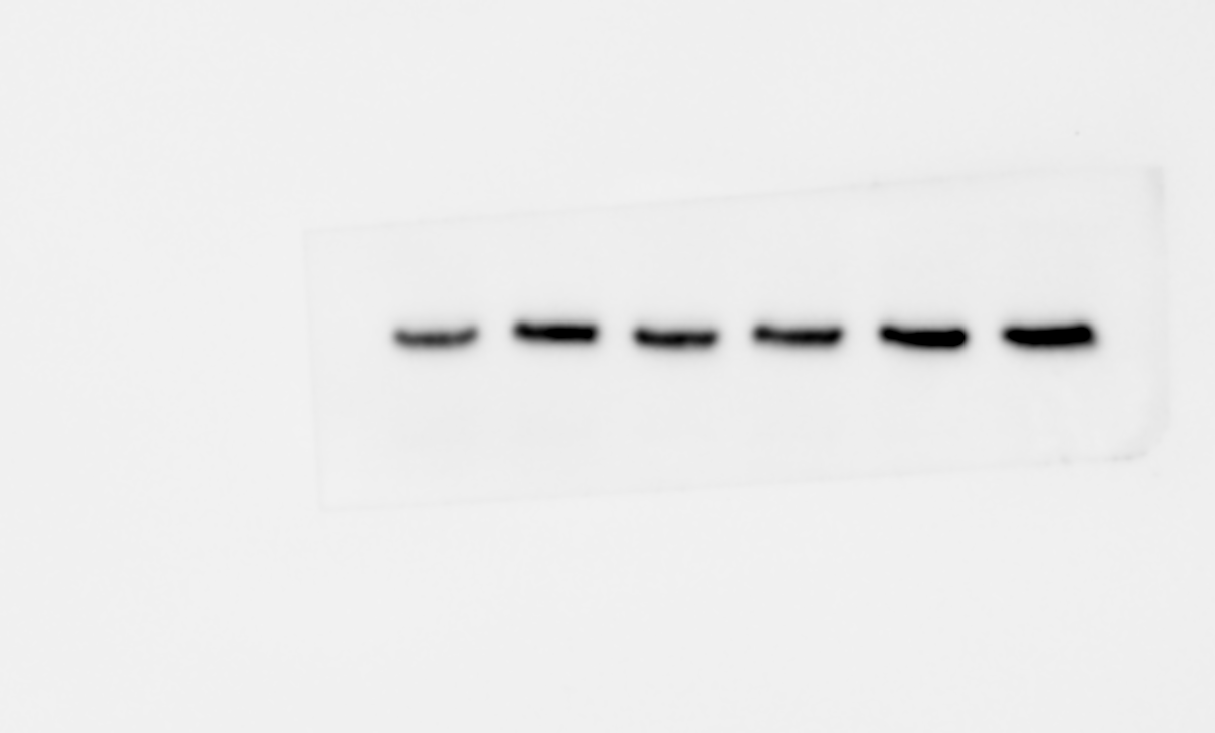

Supplement: Supplementary file 2 [file Data_Sheet_2.ZIP › WB/Figure 3/laminb/3.tif]

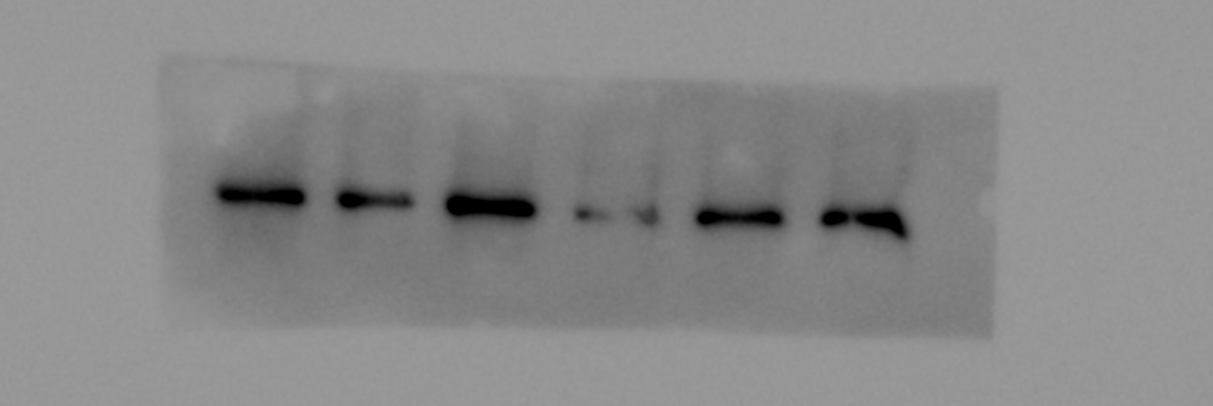

Supplement: Supplementary file 2 [file Data_Sheet_2.ZIP › WB/Figure 3/MKP1/1.tif]

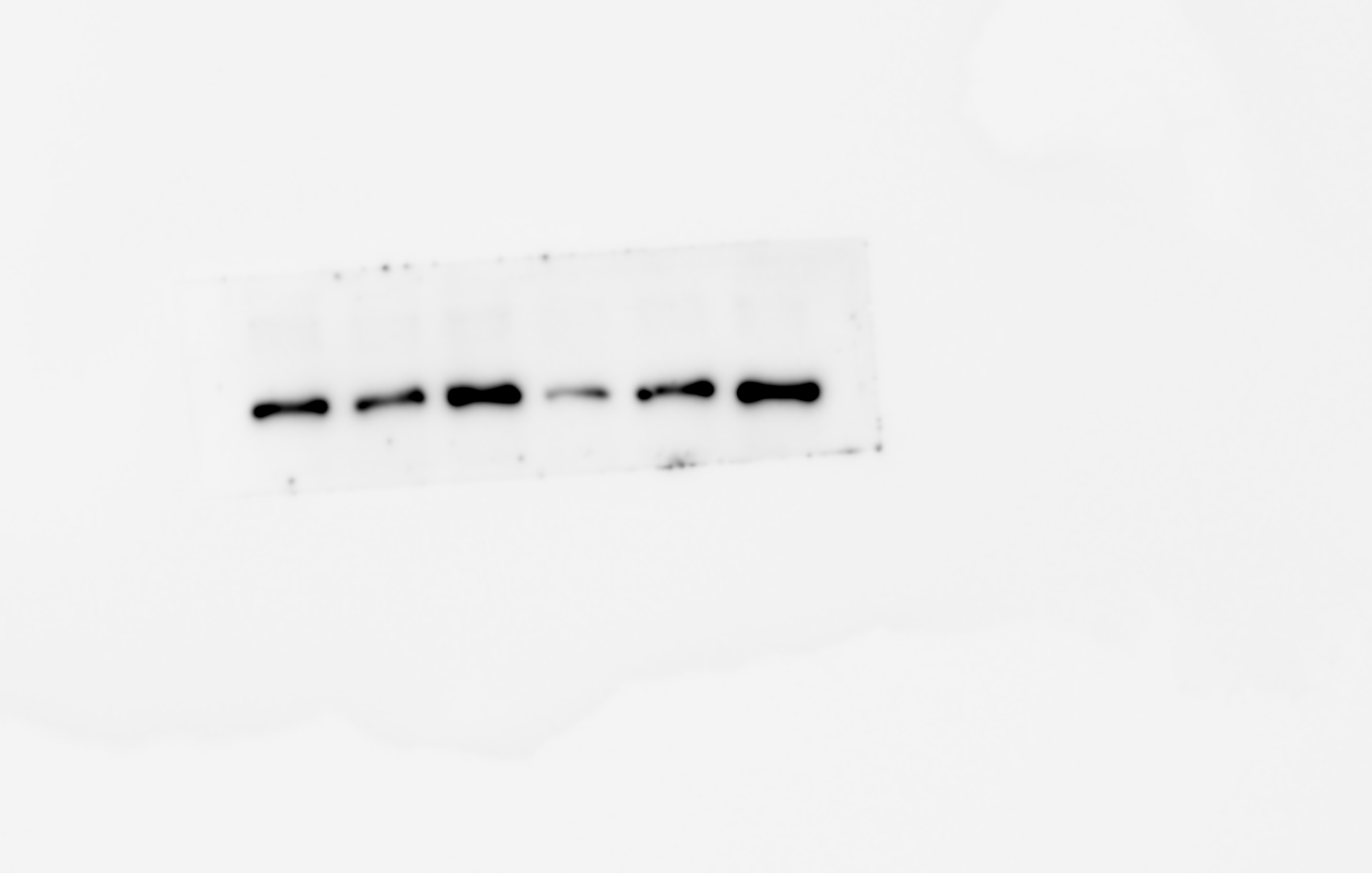

Supplement: Supplementary file 2 [file Data_Sheet_2.ZIP › WB/Figure 3/MKP1/2.tif]

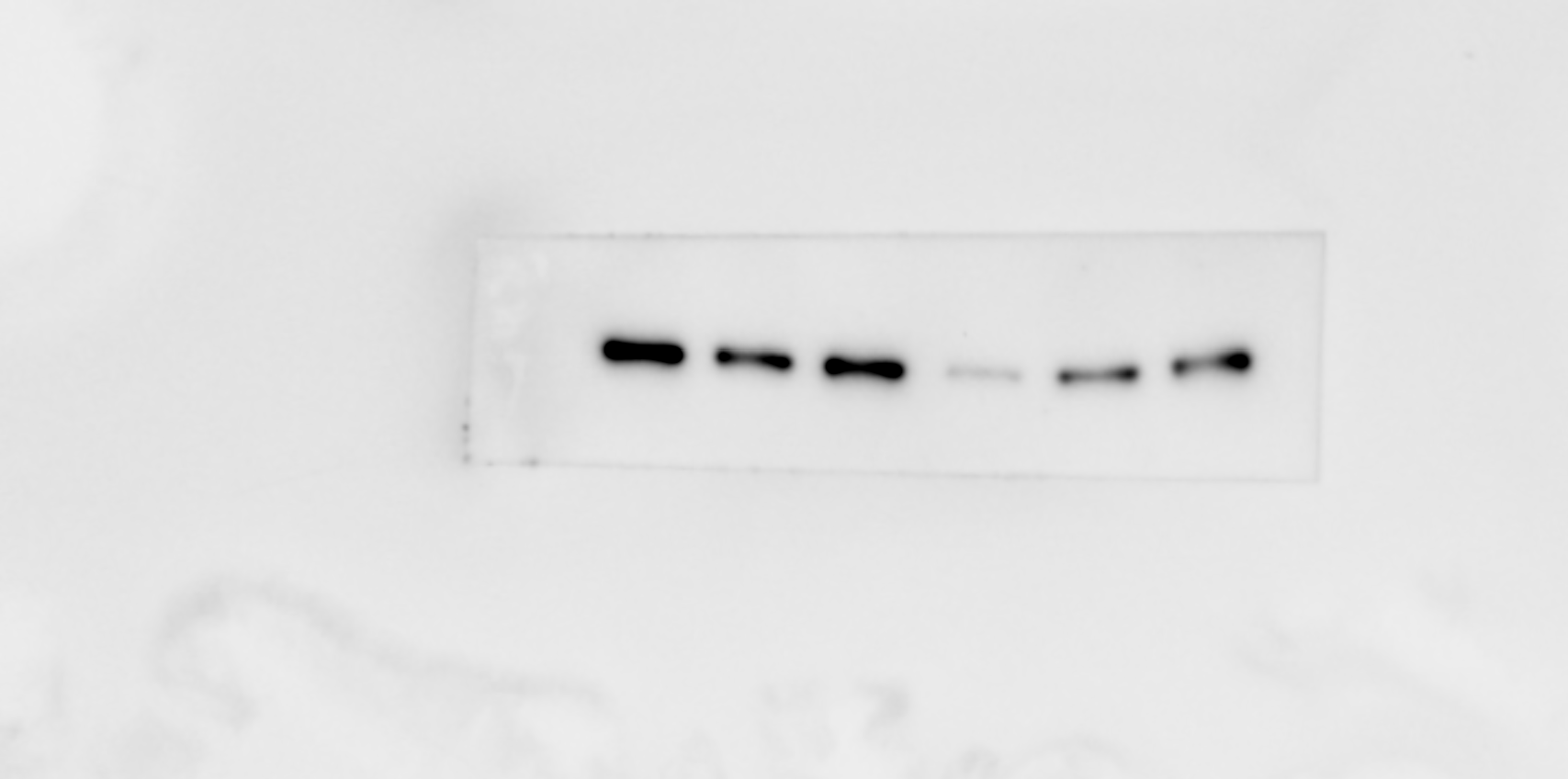

Supplement: Supplementary file 2 [file Data_Sheet_2.ZIP › WB/Figure 3/MKP1/3.tif]

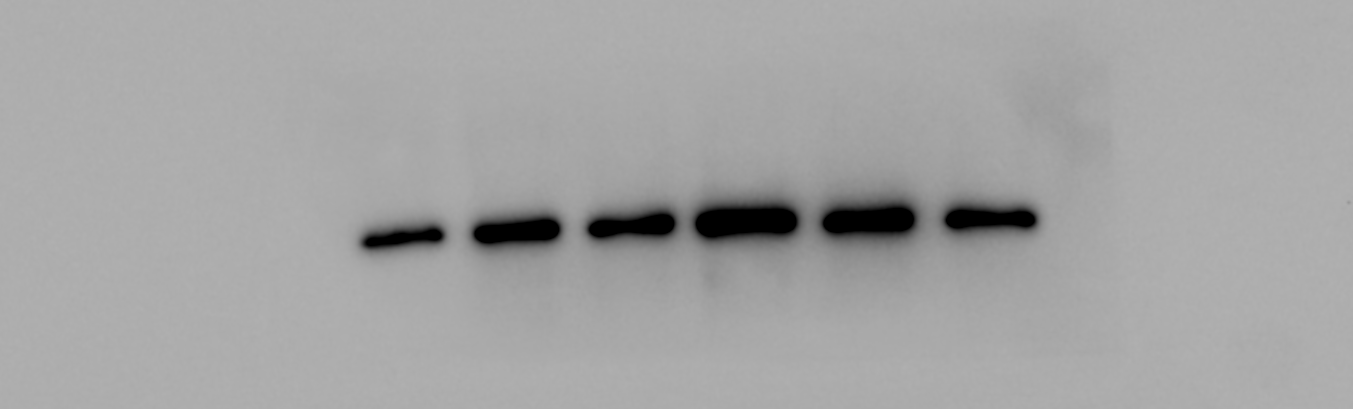

Supplement: Supplementary file 2 [file Data_Sheet_2.ZIP › WB/Figure 3/p-p38/1.tif]

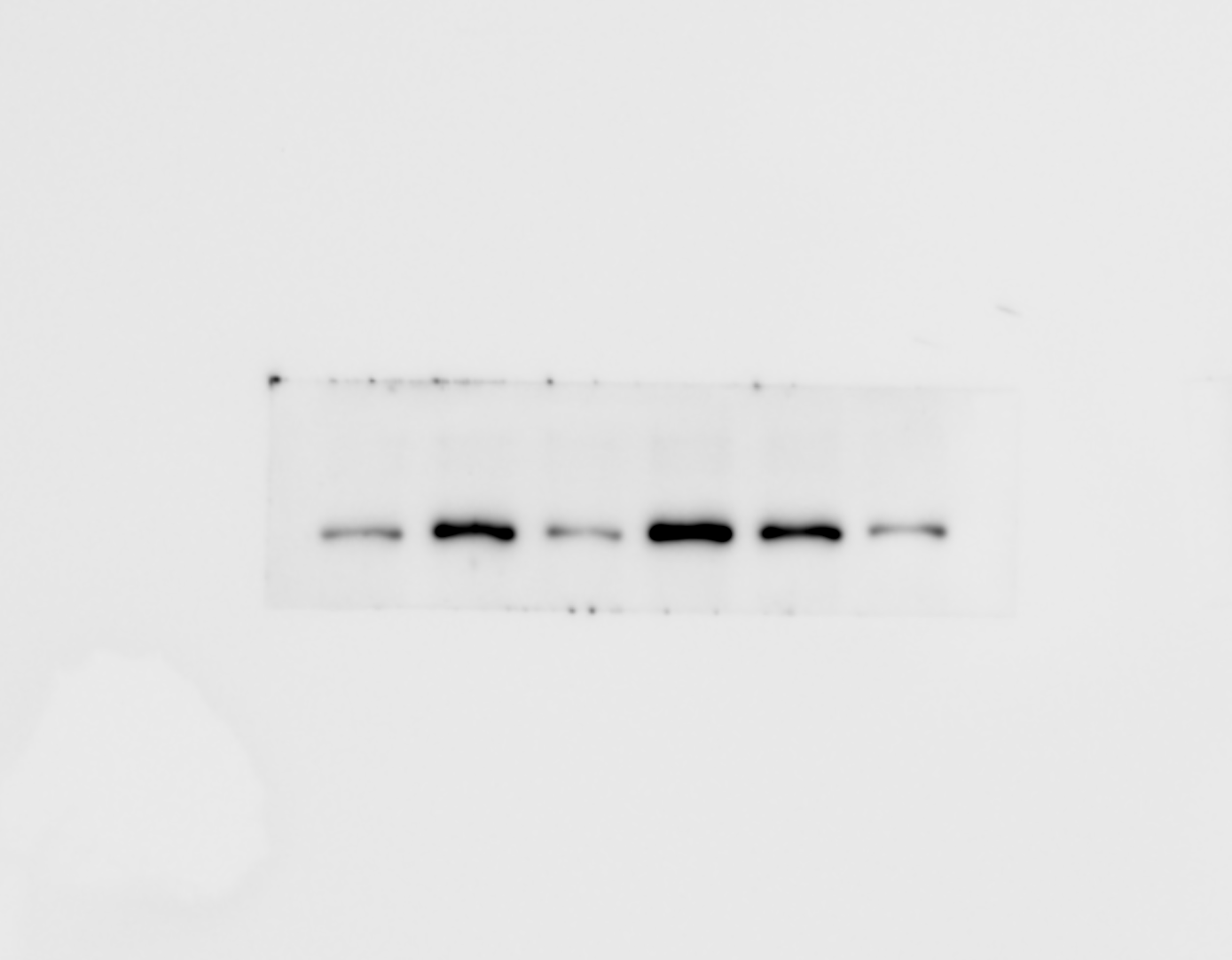

Supplement: Supplementary file 2 [file Data_Sheet_2.ZIP › WB/Figure 3/p-p38/2.tif]

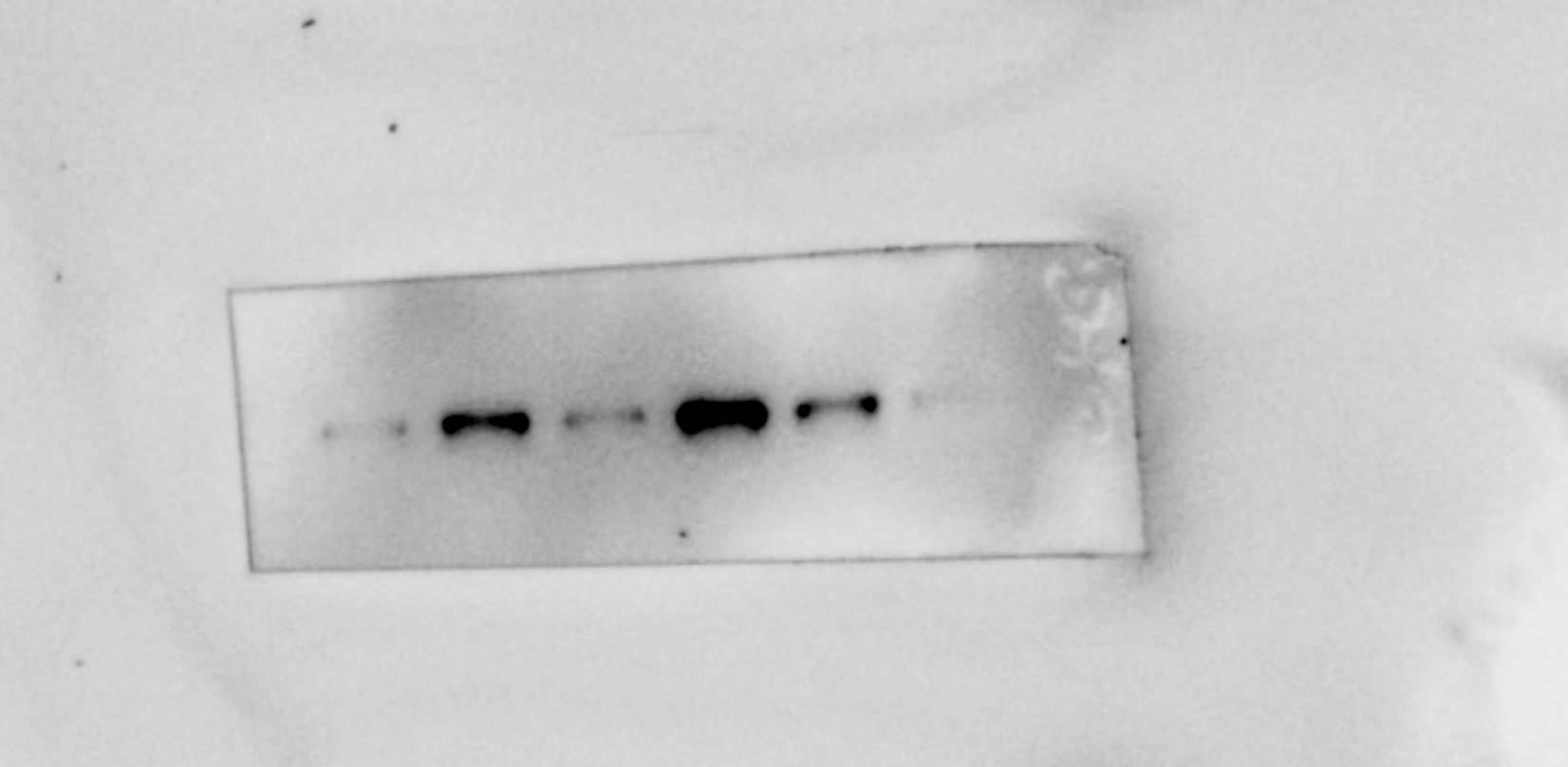

Supplement: Supplementary file 2 [file Data_Sheet_2.ZIP › WB/Figure 3/p-p38/3.tif]

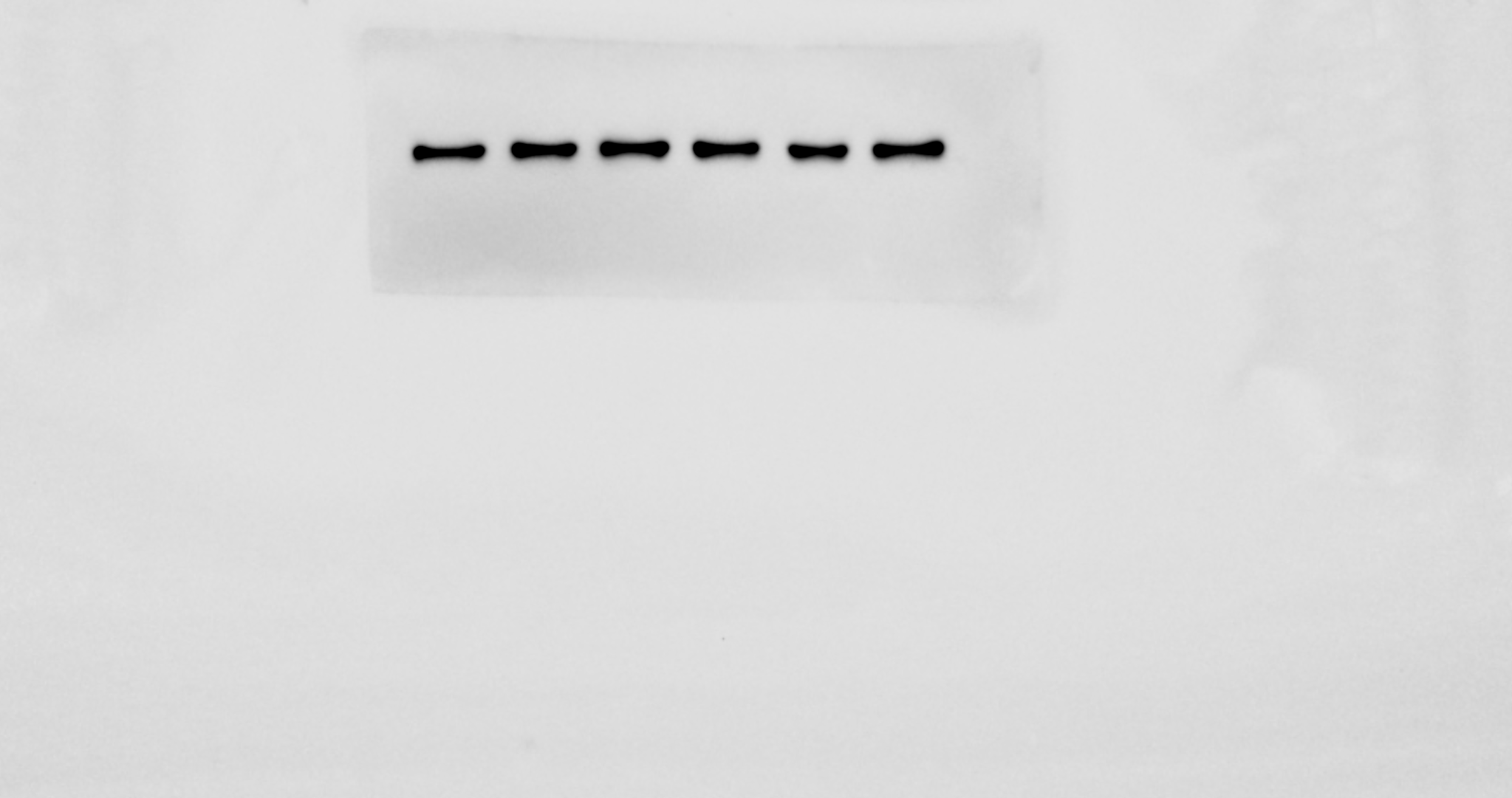

Supplement: Supplementary file 2 [file Data_Sheet_2.ZIP › WB/Figure 3/p38/1.tif]

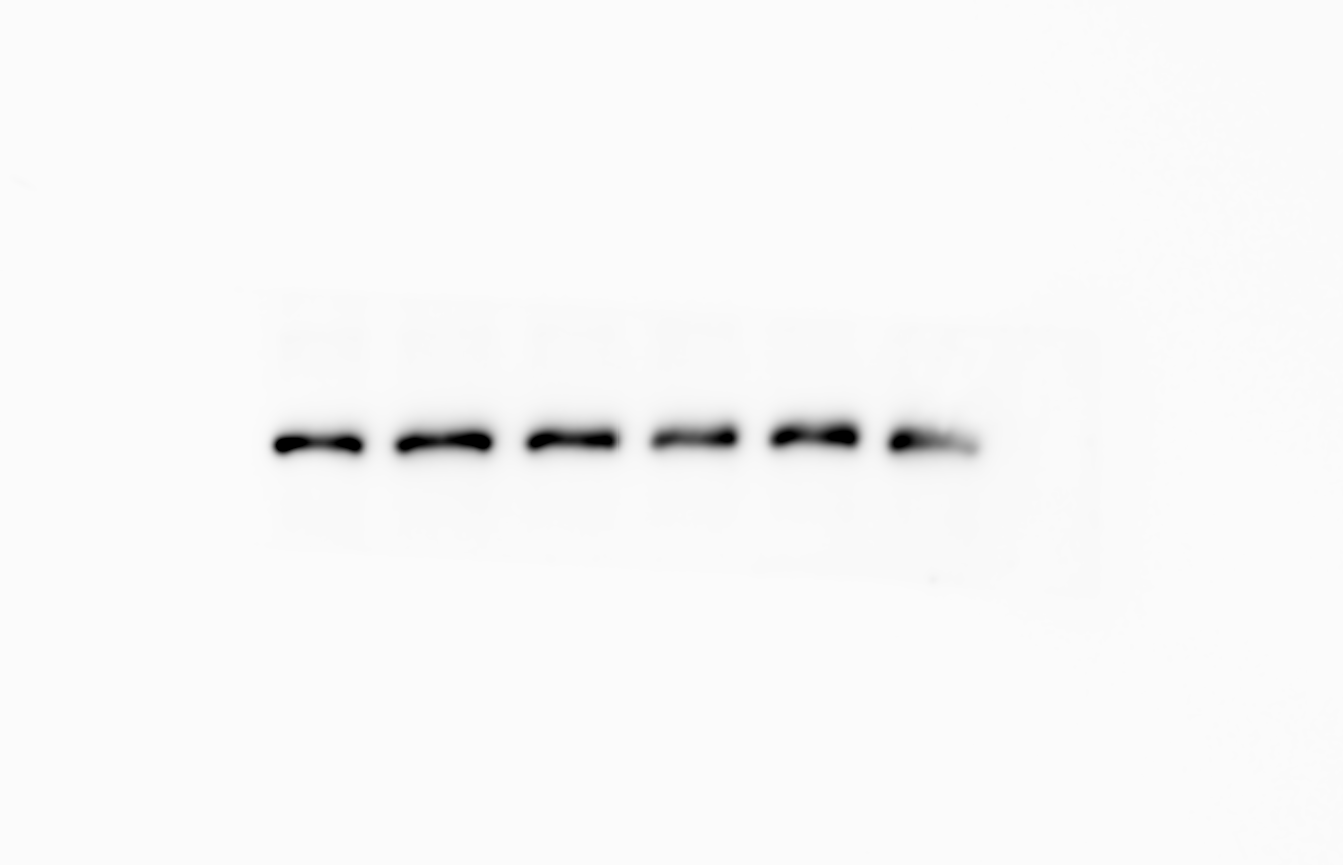

Supplement: Supplementary file 2 [file Data_Sheet_2.ZIP › WB/Figure 3/p38/2.tif]

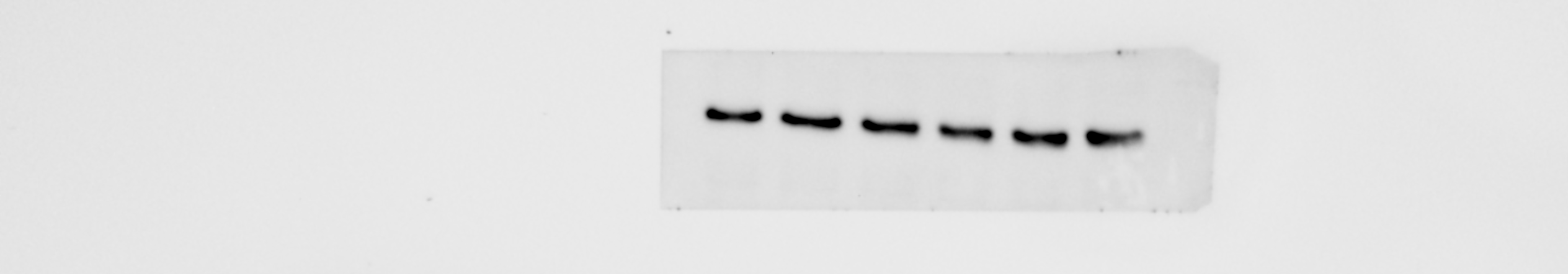

Supplement: Supplementary file 2 [file Data_Sheet_2.ZIP › WB/Figure 3/p38/3.tif]

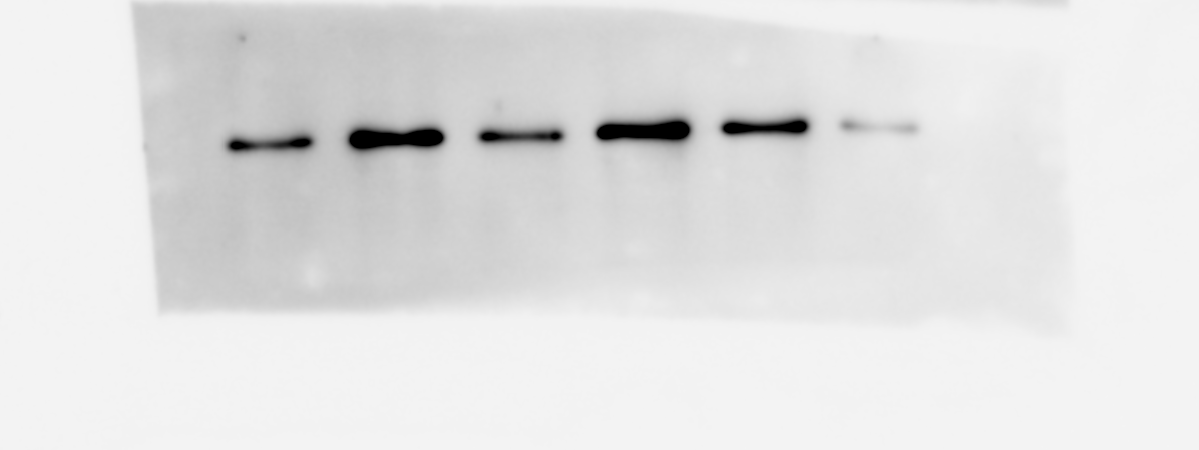

Supplement: Supplementary file 2 [file Data_Sheet_2.ZIP › WB/Figure 3/p65/1.tif]

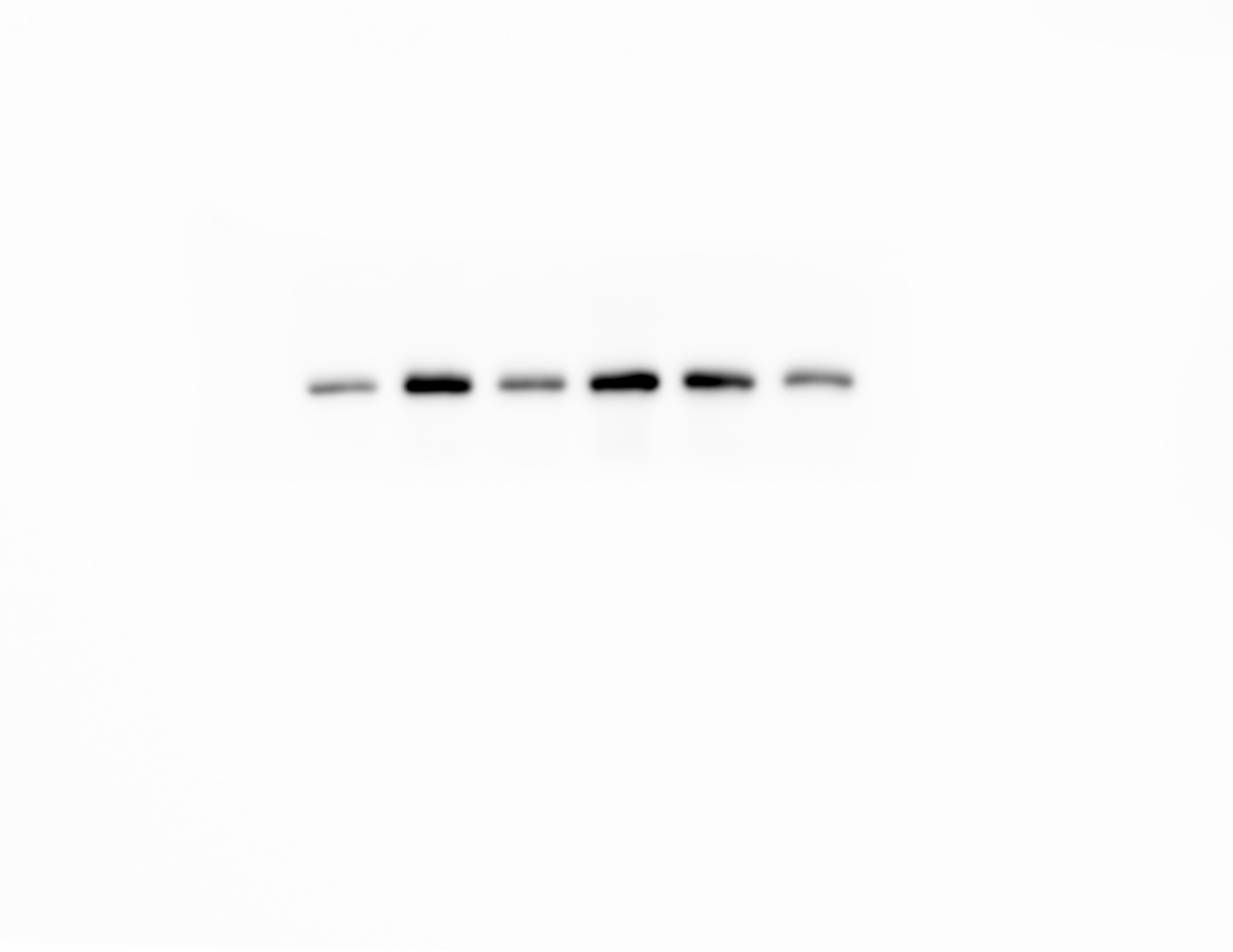

Supplement: Supplementary file 2 [file Data_Sheet_2.ZIP › WB/Figure 3/p65/2.tif]

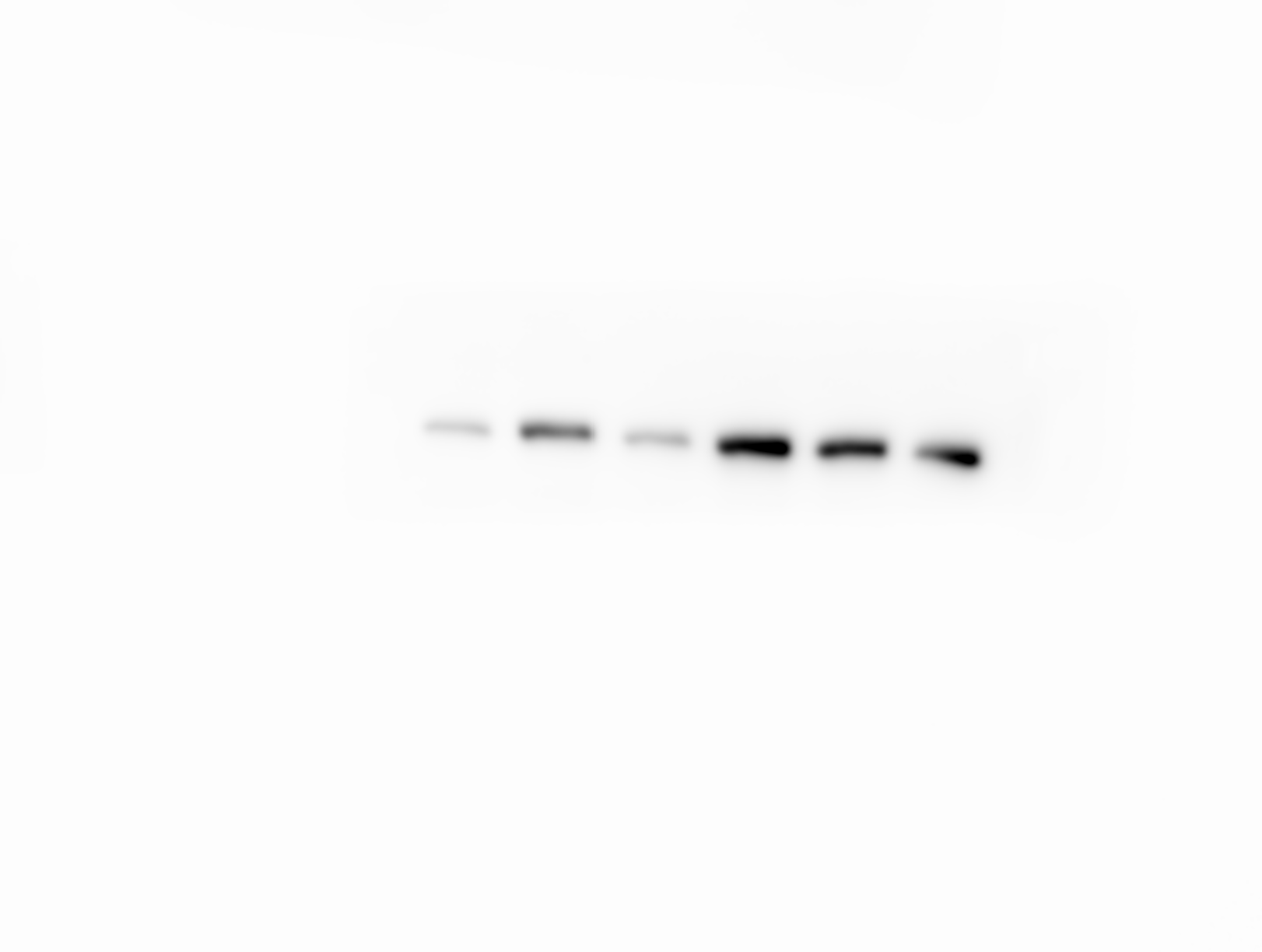

Supplement: Supplementary file 2 [file Data_Sheet_2.ZIP › WB/Figure 3/p65/3.tif]

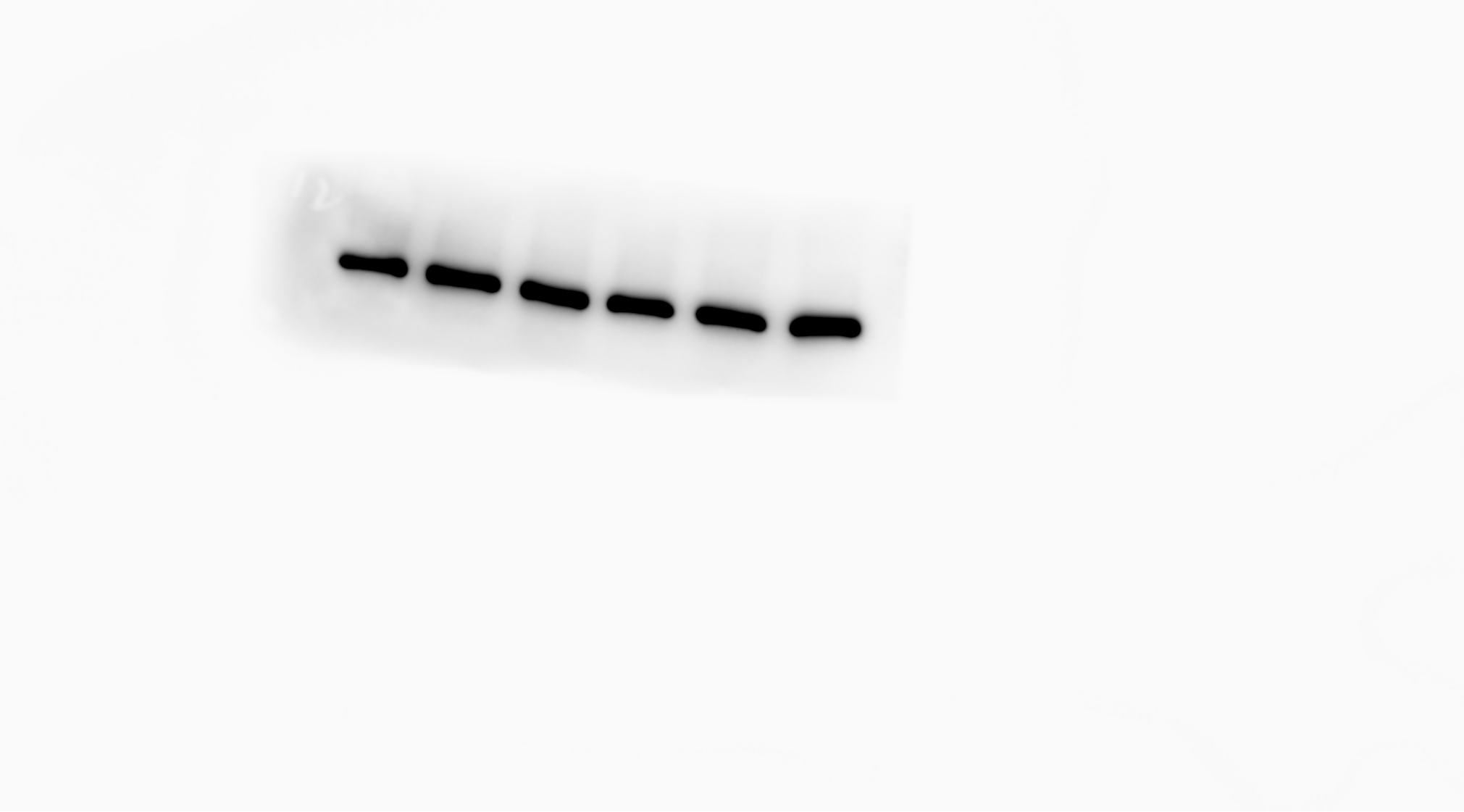

Supplement: Supplementary file 2 [file Data_Sheet_2.ZIP › WB/Figure 4/actin/1.tif]

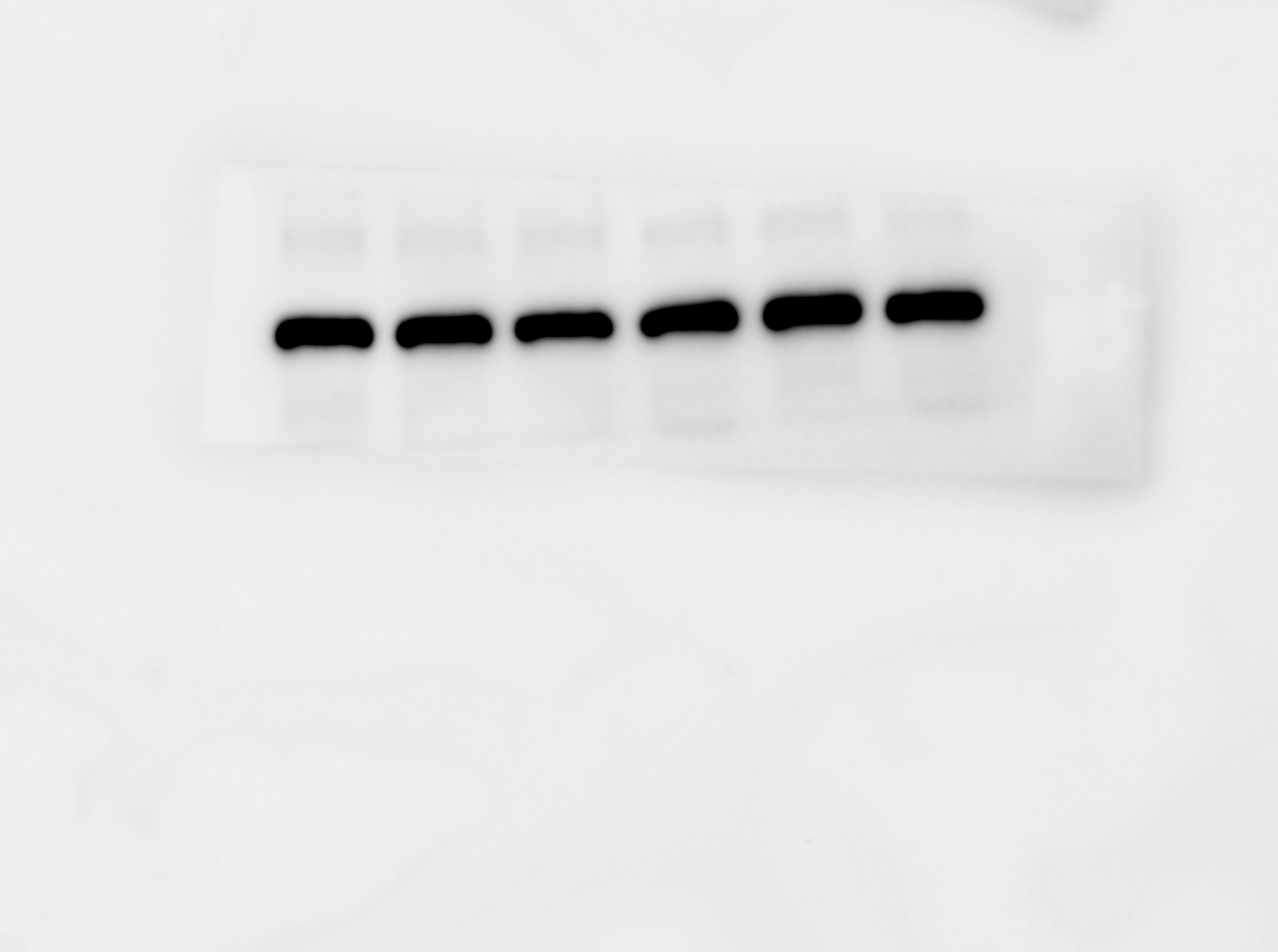

Supplement: Supplementary file 2 [file Data_Sheet_2.ZIP › WB/Figure 4/actin/2.tif]

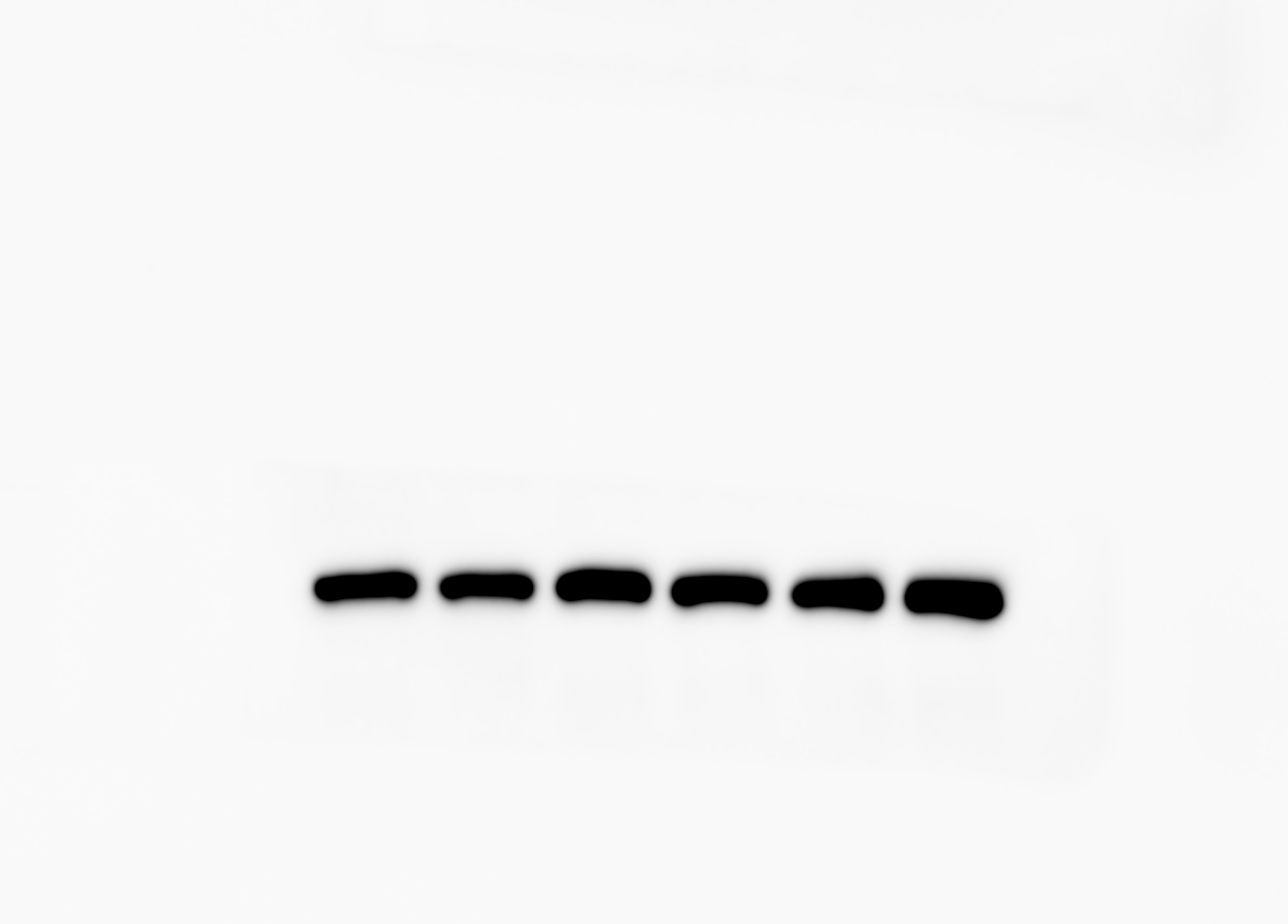

Supplement: Supplementary file 2 [file Data_Sheet_2.ZIP › WB/Figure 4/actin/3.tif]

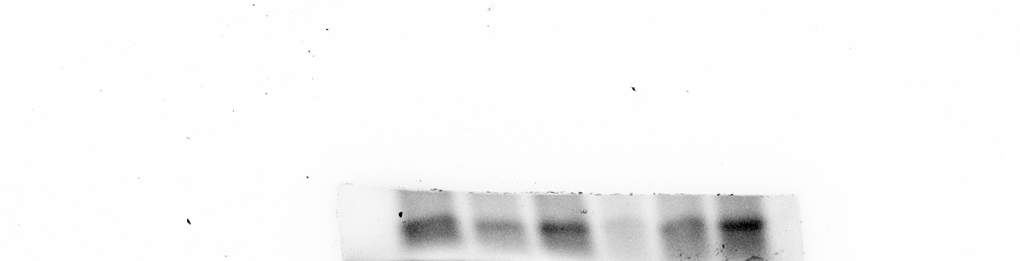

Supplement: Supplementary file 2 [file Data_Sheet_2.ZIP › WB/Figure 4/HO-1/1.tif]

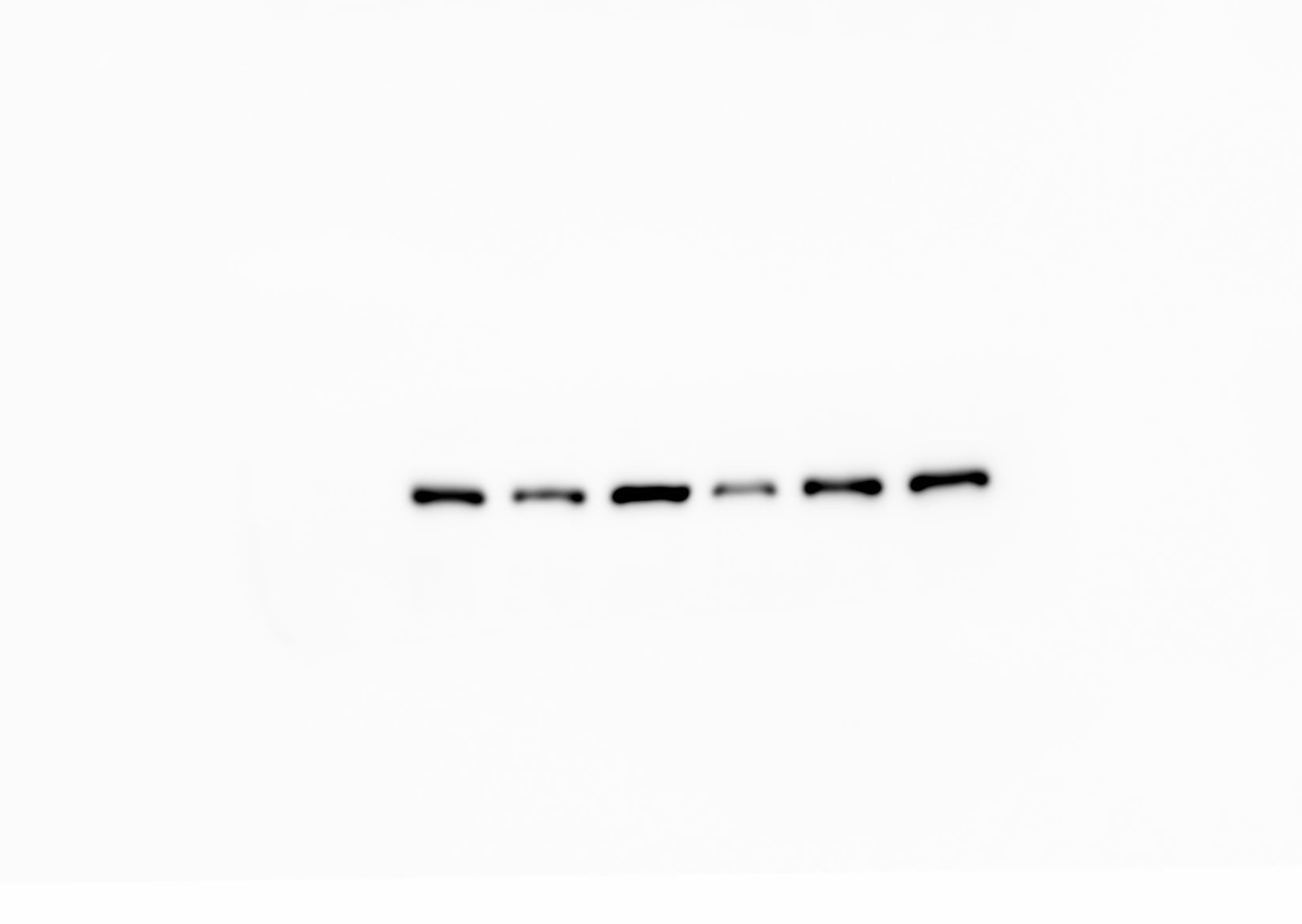

Supplement: Supplementary file 2 [file Data_Sheet_2.ZIP › WB/Figure 4/HO-1/2.tif]

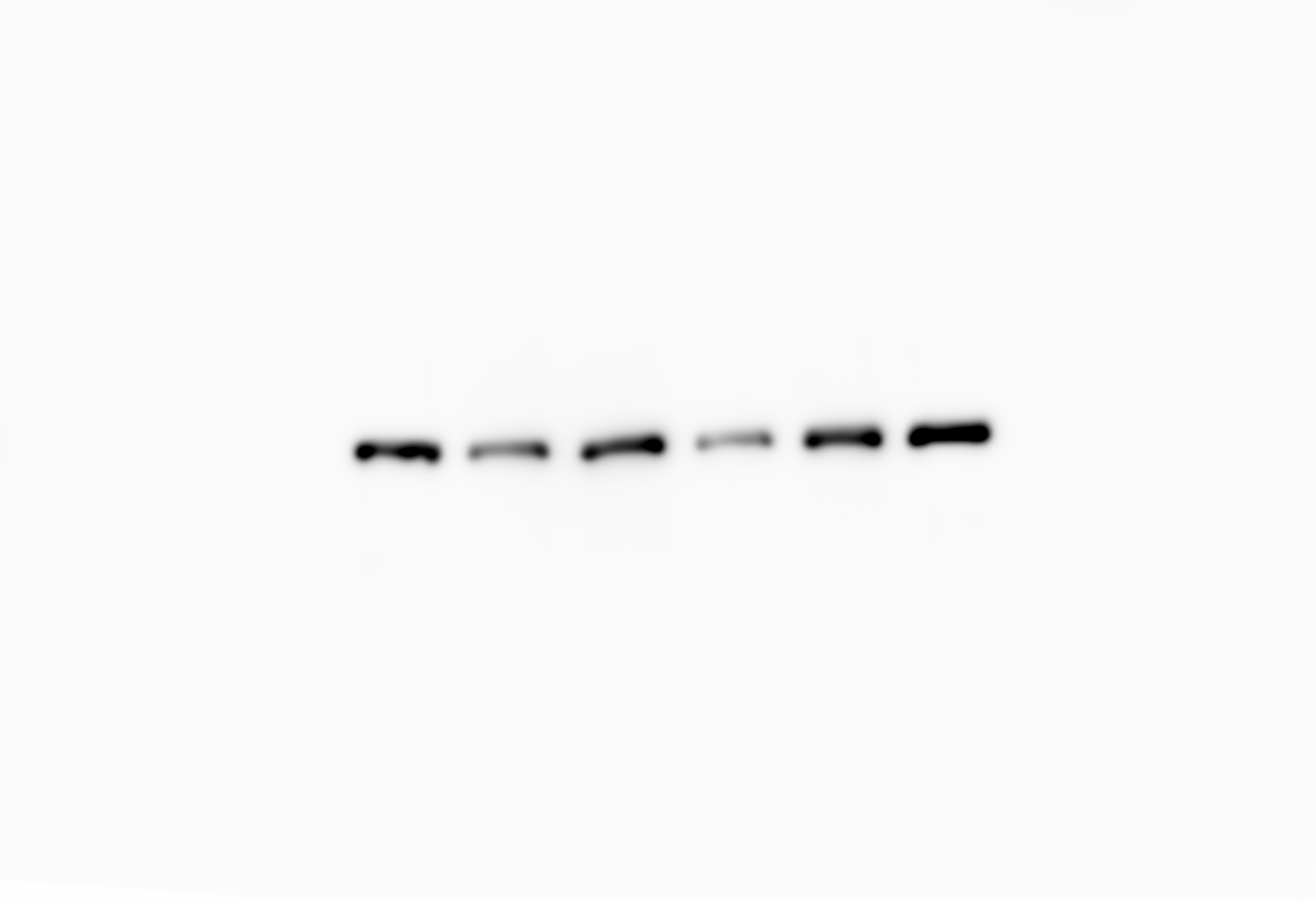

Supplement: Supplementary file 2 [file Data_Sheet_2.ZIP › WB/Figure 4/HO-1/3.tif]

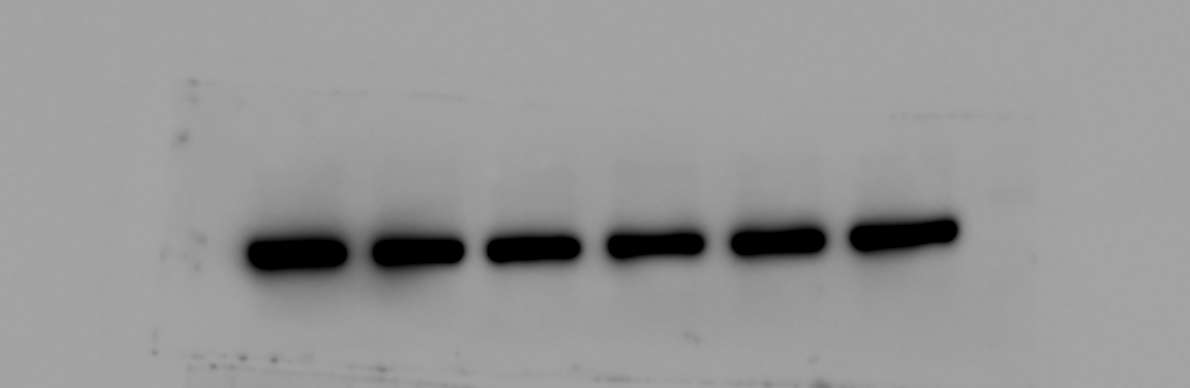

Supplement: Supplementary file 2 [file Data_Sheet_2.ZIP › WB/Figure 4/laminb/1.tif]

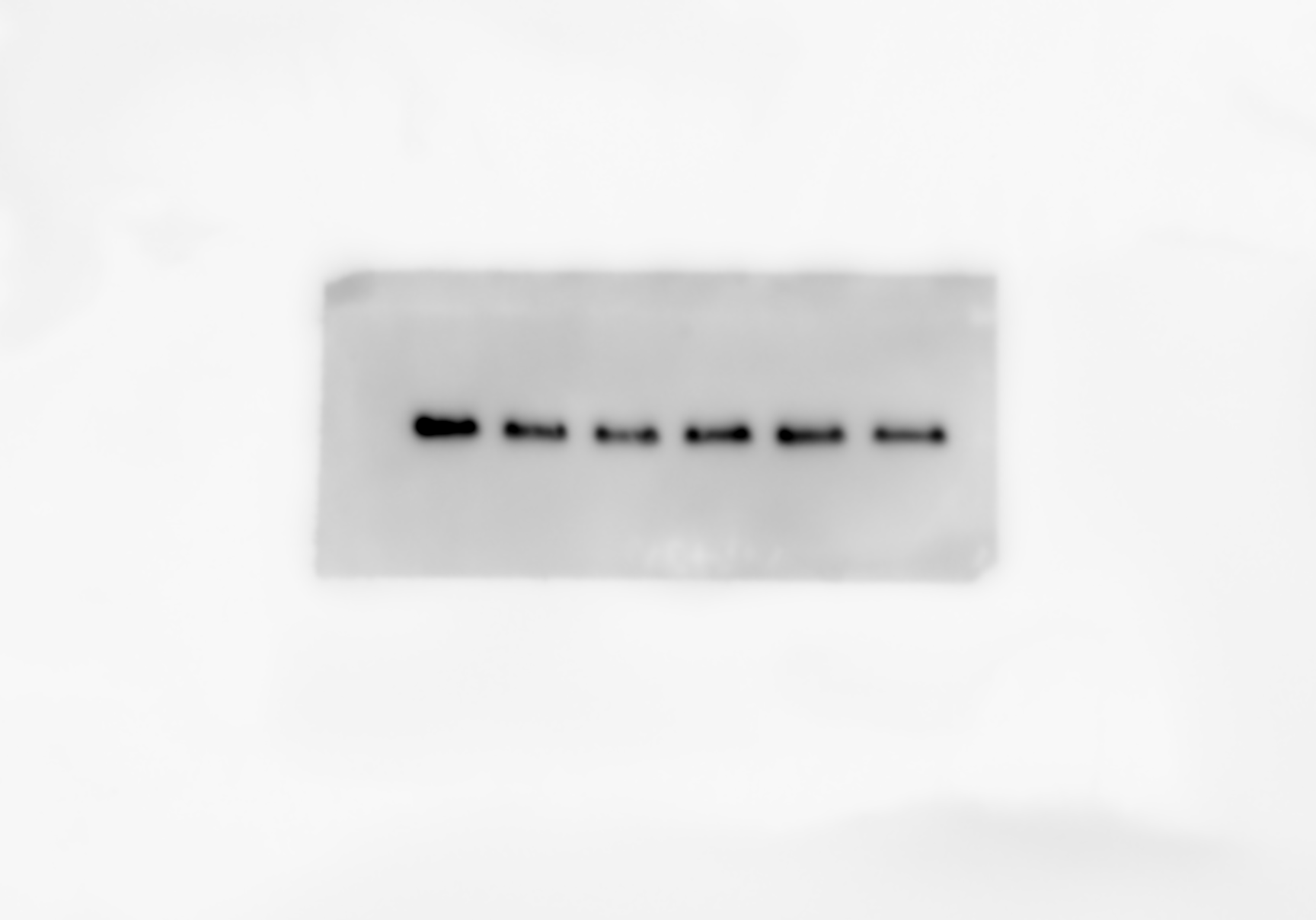

Supplement: Supplementary file 2 [file Data_Sheet_2.ZIP › WB/Figure 4/laminb/2.tif]

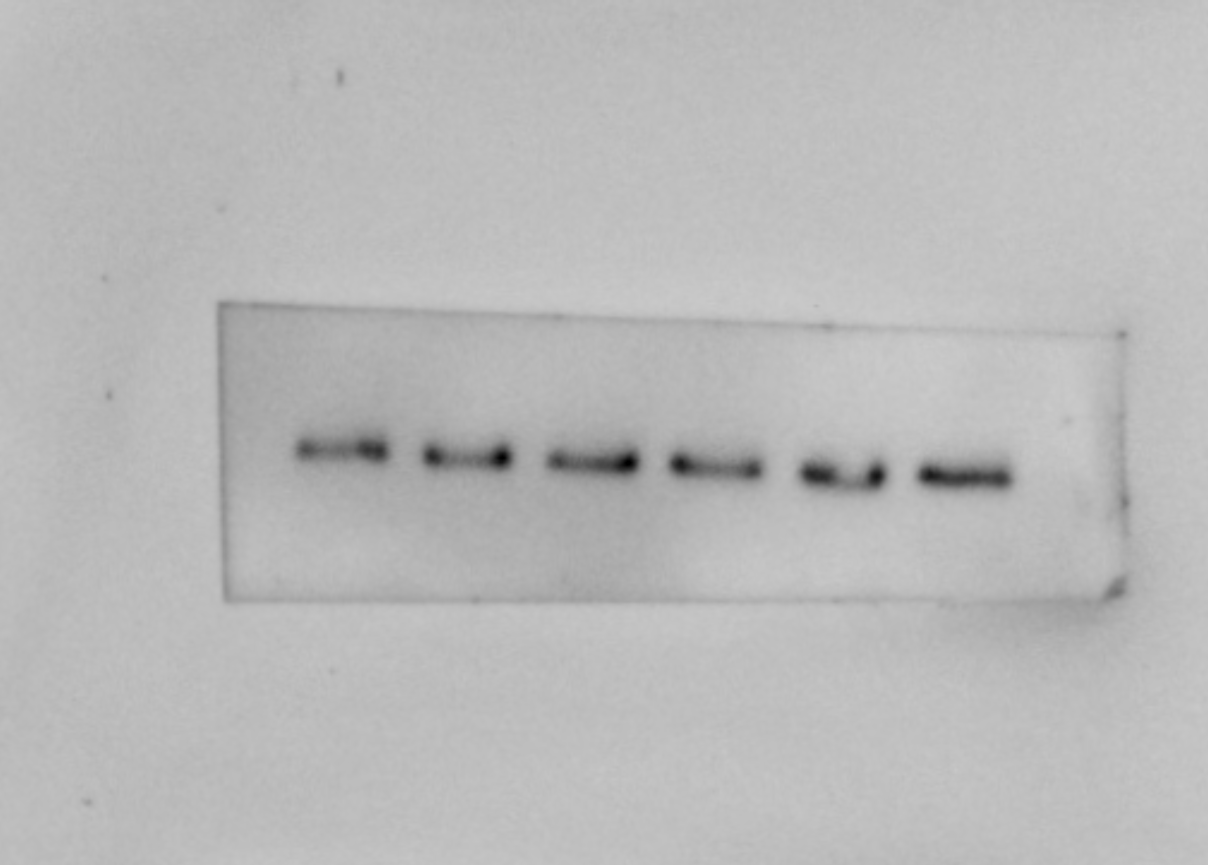

Supplement: Supplementary file 2 [file Data_Sheet_2.ZIP › WB/Figure 4/laminb/3.tif]

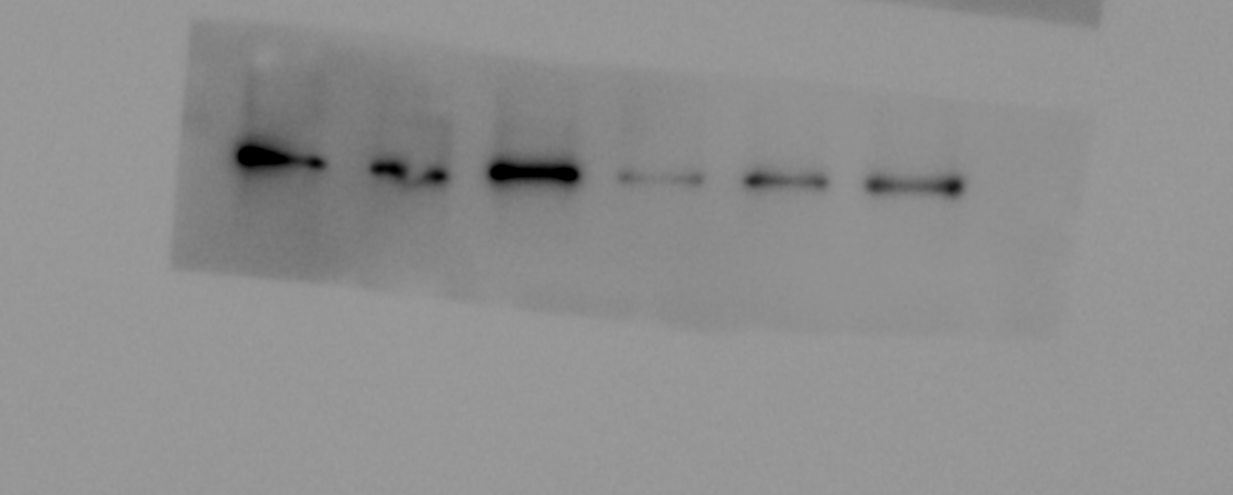

Supplement: Supplementary file 2 [file Data_Sheet_2.ZIP › WB/Figure 4/MKP1/1.tif]

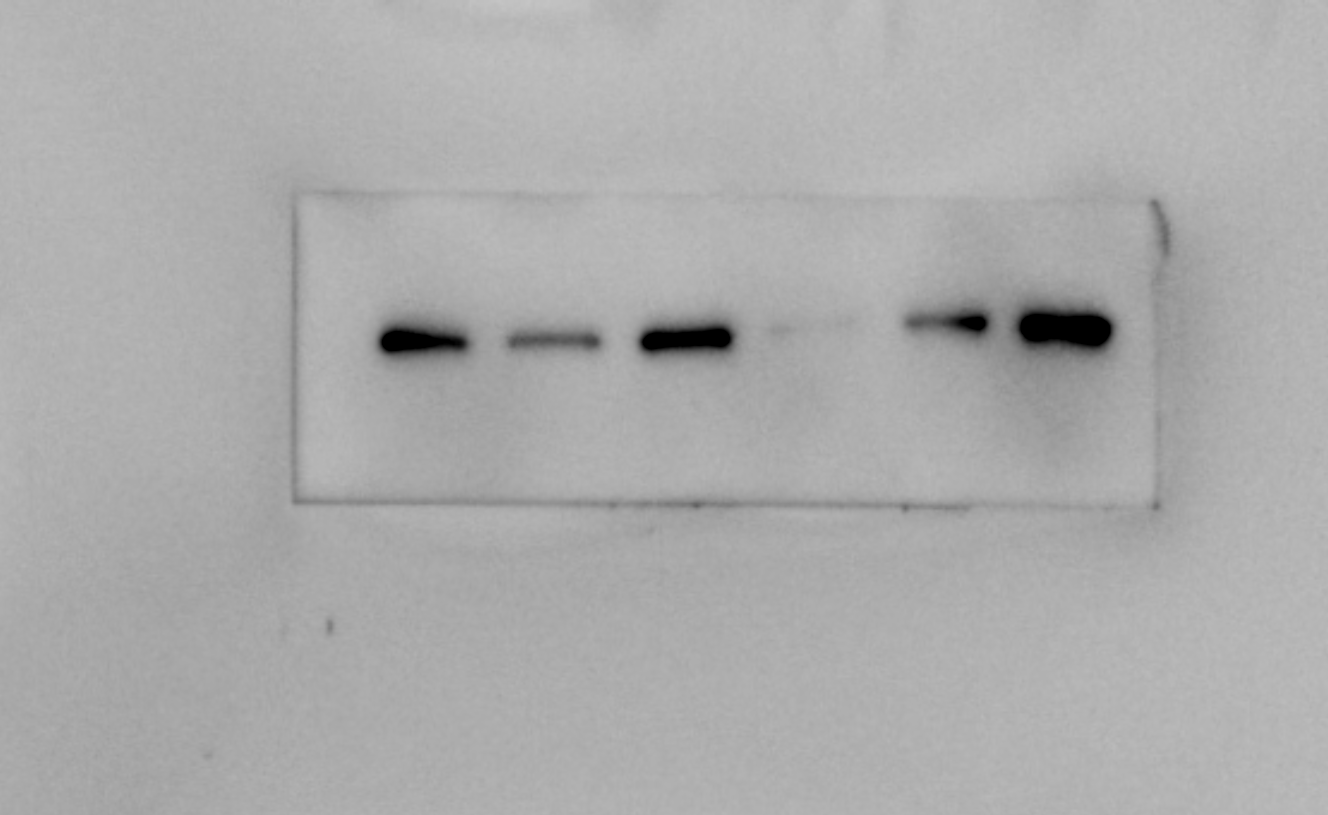

Supplement: Supplementary file 2 [file Data_Sheet_2.ZIP › WB/Figure 4/MKP1/2.tif]

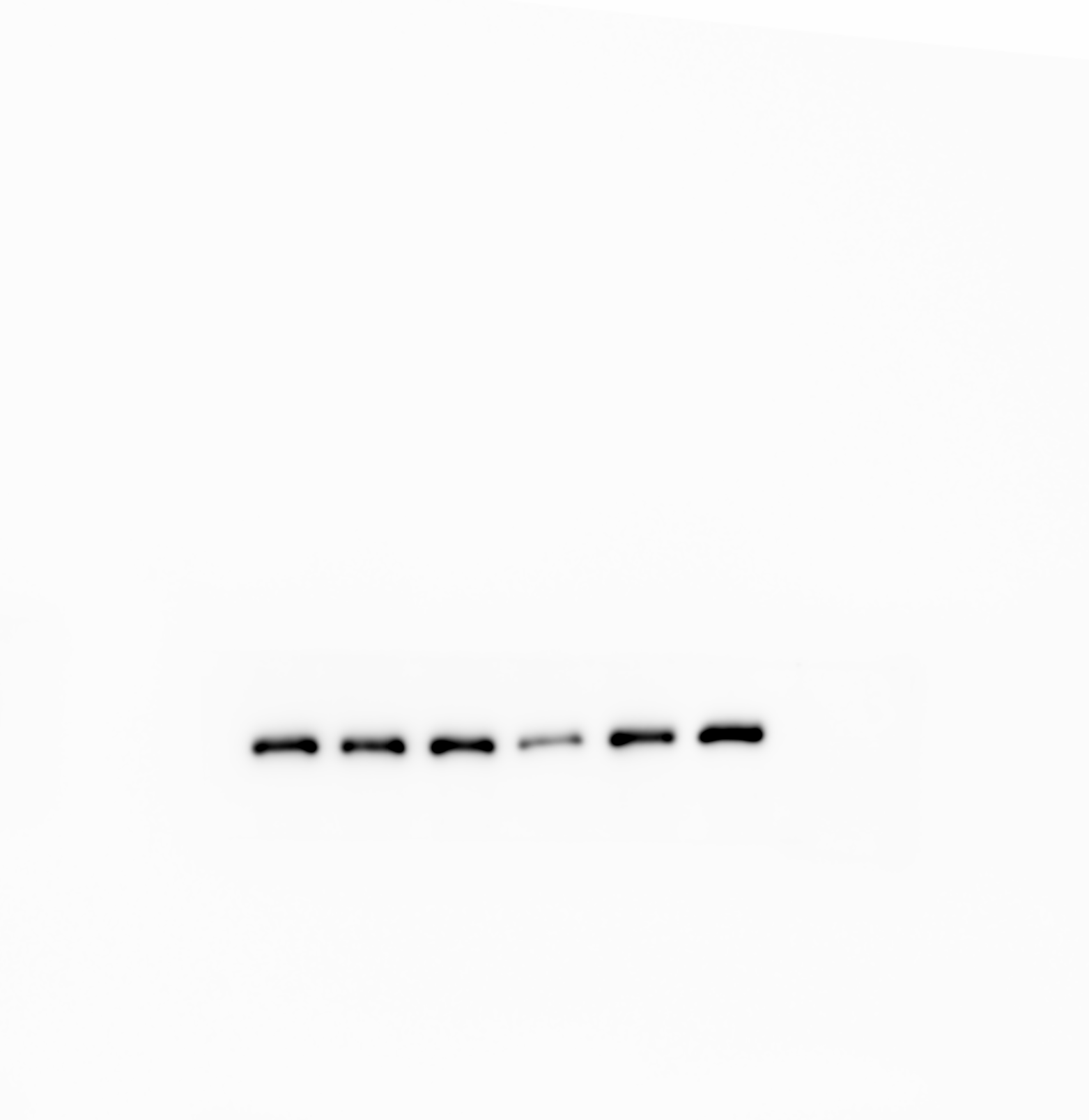

Supplement: Supplementary file 2 [file Data_Sheet_2.ZIP › WB/Figure 4/MKP1/3.tif]

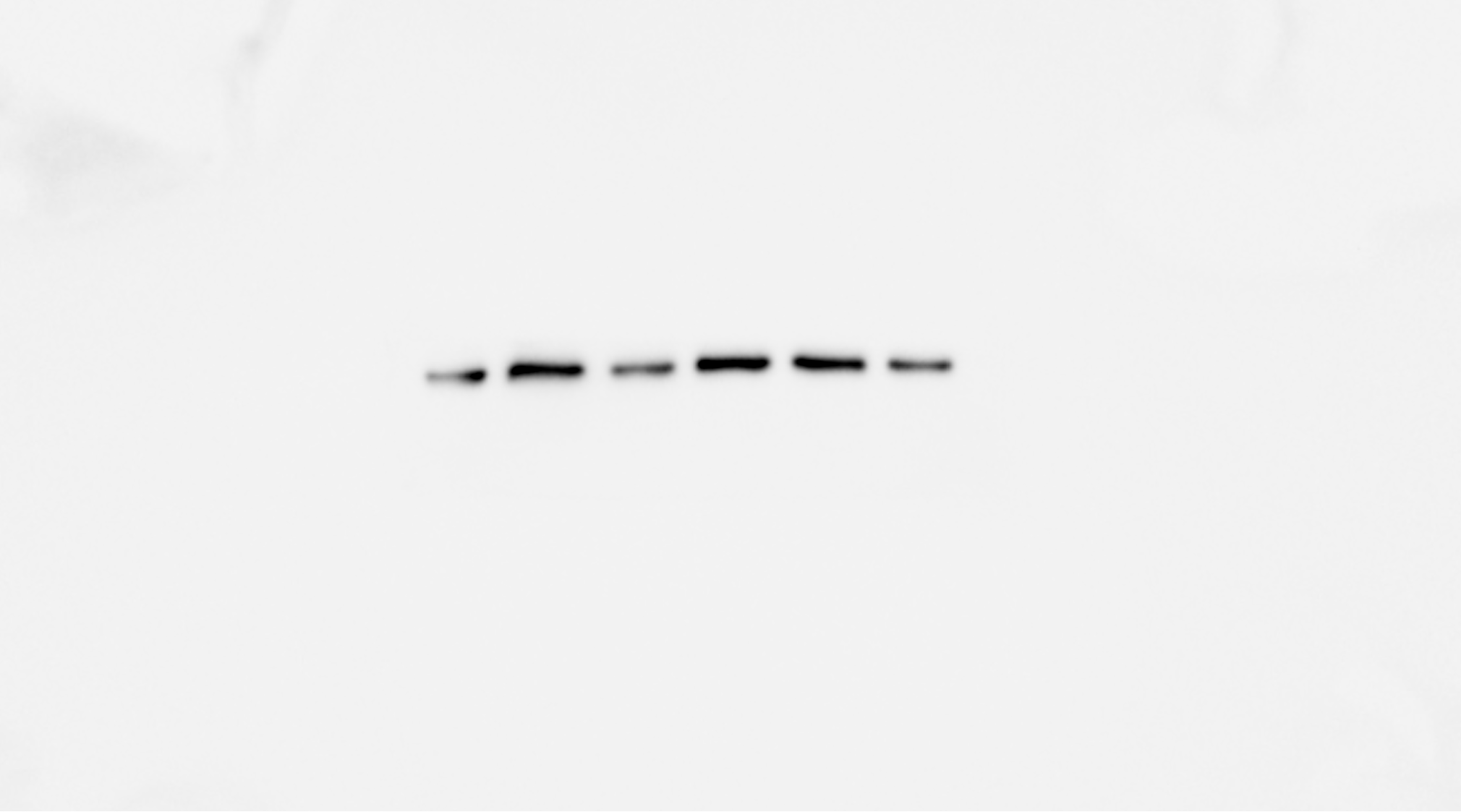

Supplement: Supplementary file 2 [file Data_Sheet_2.ZIP › WB/Figure 4/p-p38/1.tif]

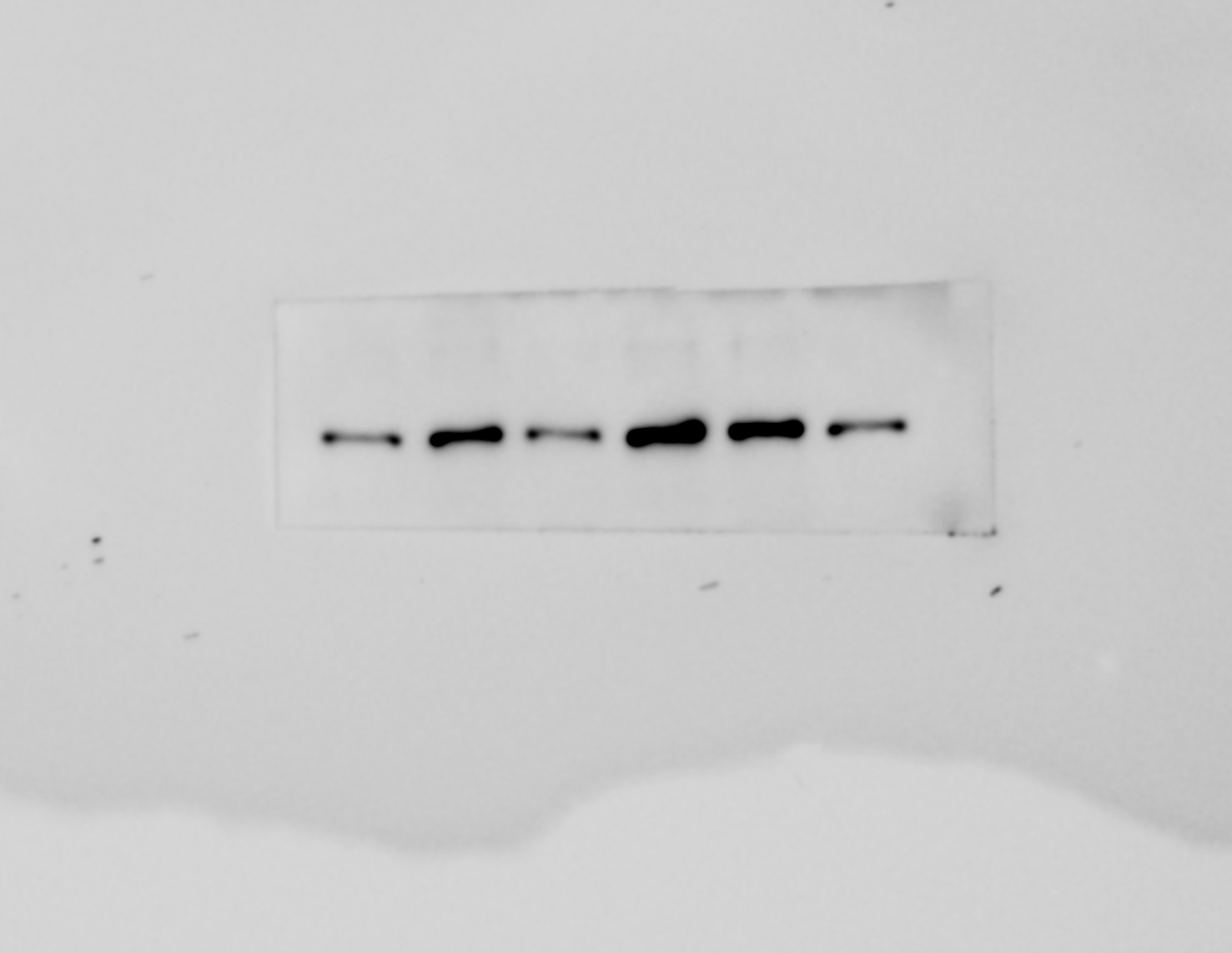

Supplement: Supplementary file 2 [file Data_Sheet_2.ZIP › WB/Figure 4/p-p38/2.tif]

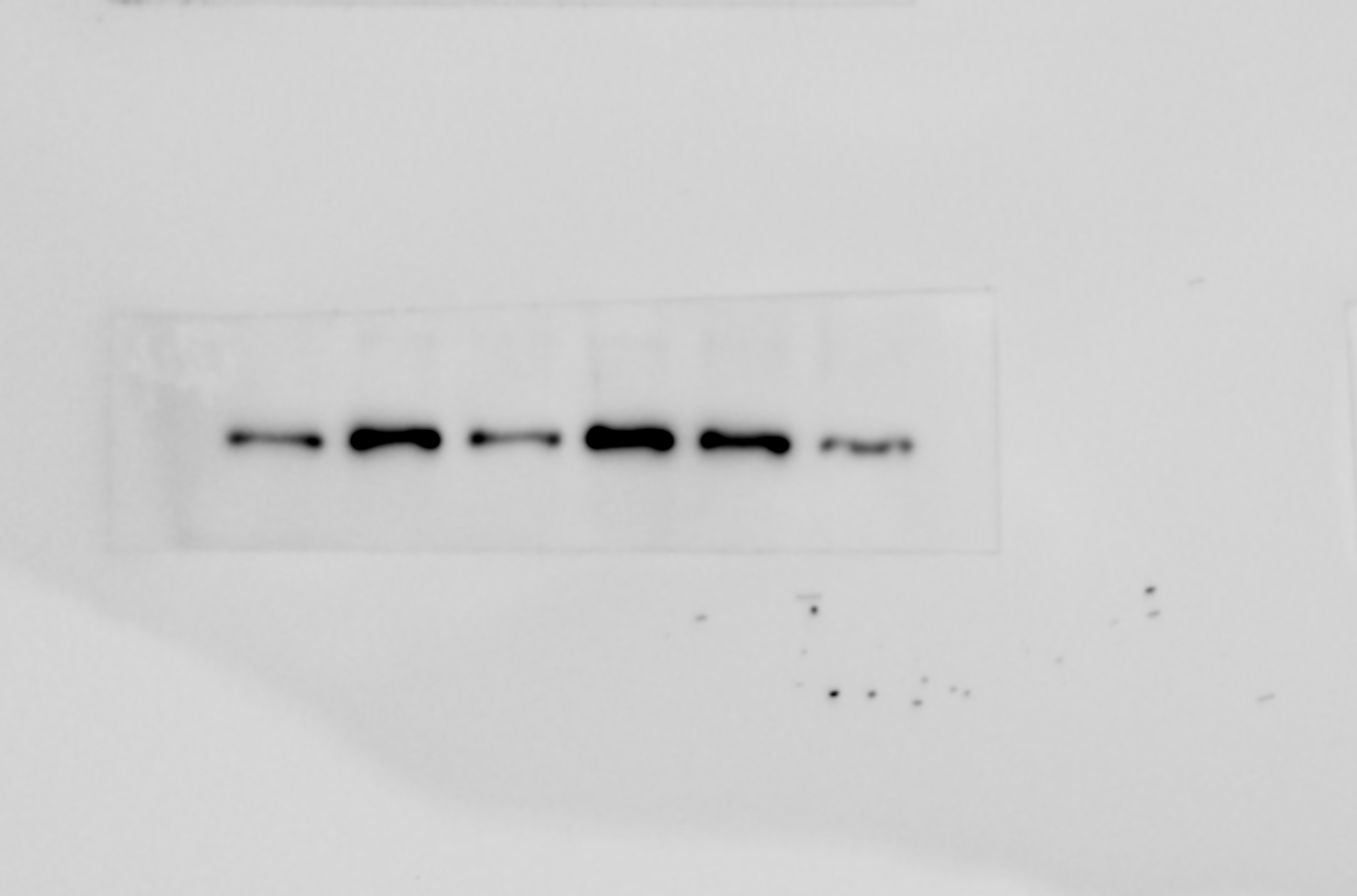

Supplement: Supplementary file 2 [file Data_Sheet_2.ZIP › WB/Figure 4/p-p38/3.tif]

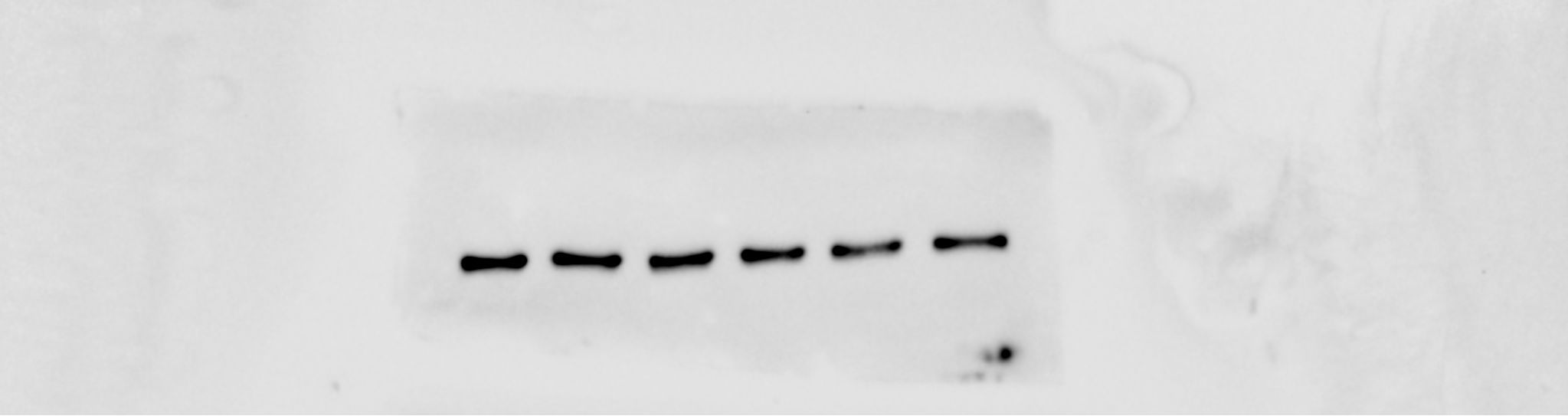

Supplement: Supplementary file 2 [file Data_Sheet_2.ZIP › WB/Figure 4/p38/1.tif]

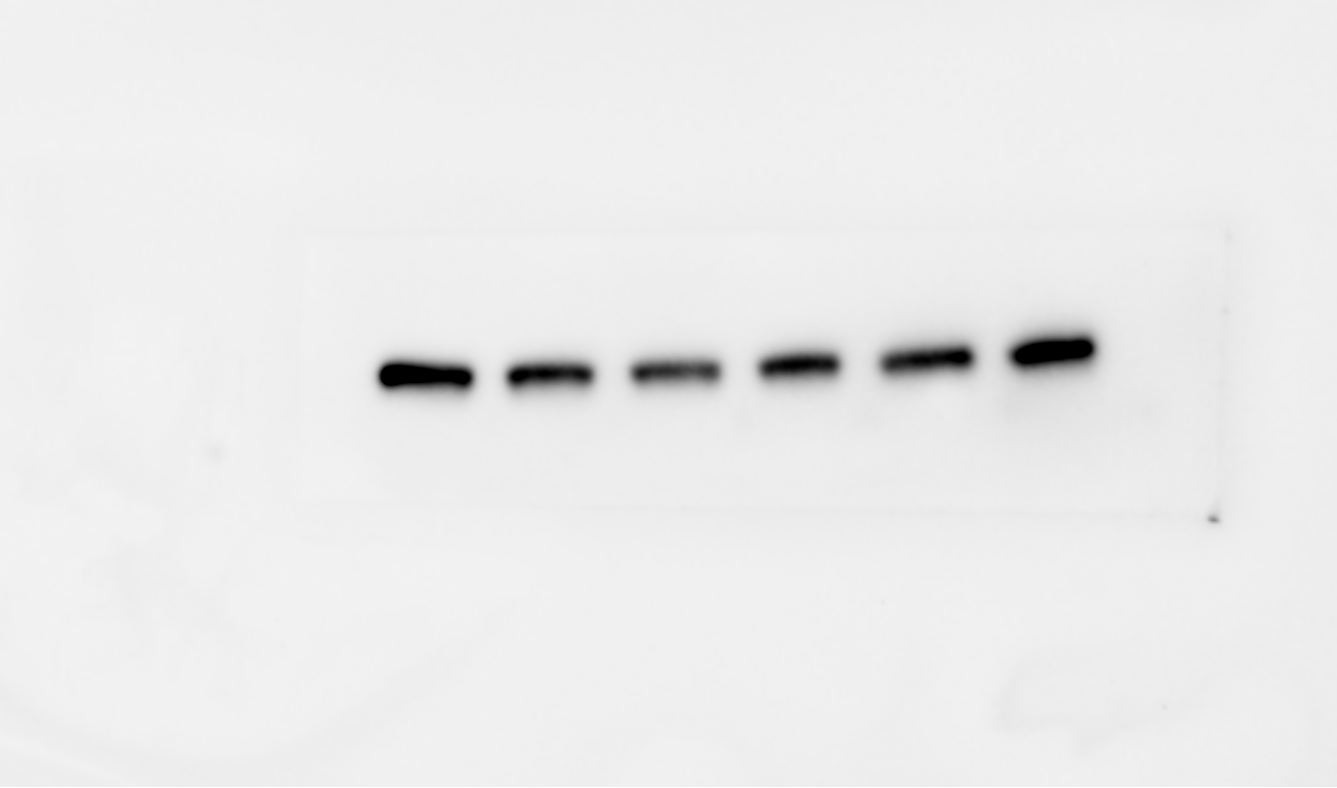

Supplement: Supplementary file 2 [file Data_Sheet_2.ZIP › WB/Figure 4/p38/2.tif]

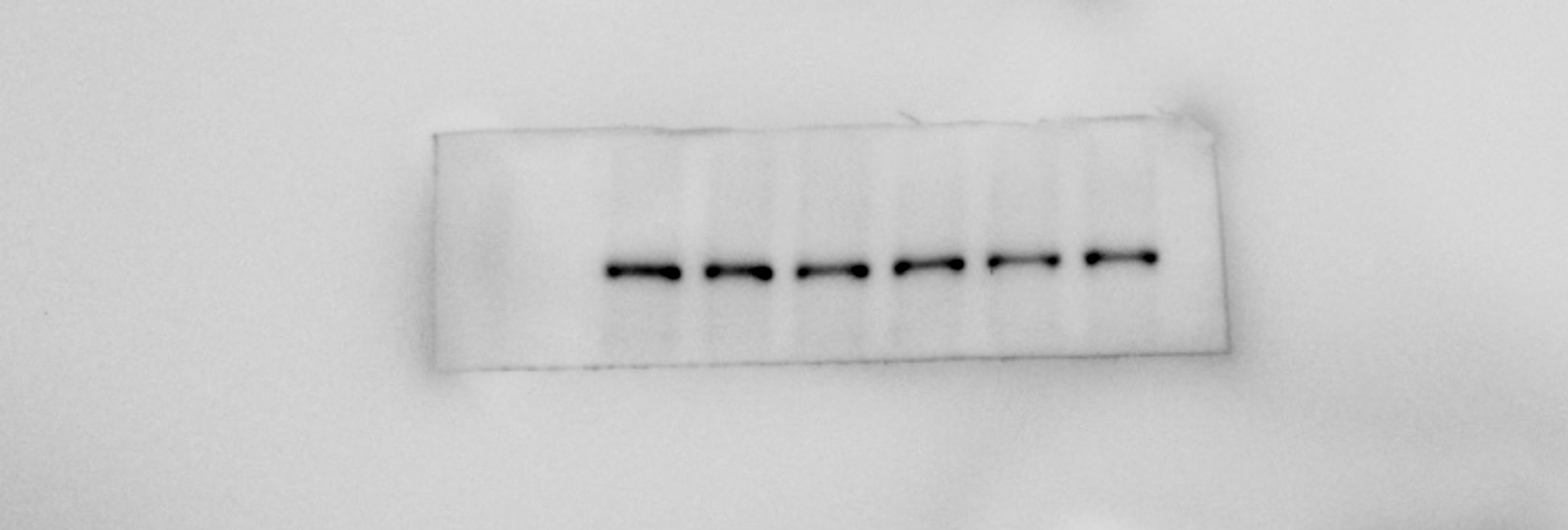

Supplement: Supplementary file 2 [file Data_Sheet_2.ZIP › WB/Figure 4/p38/3.tif]

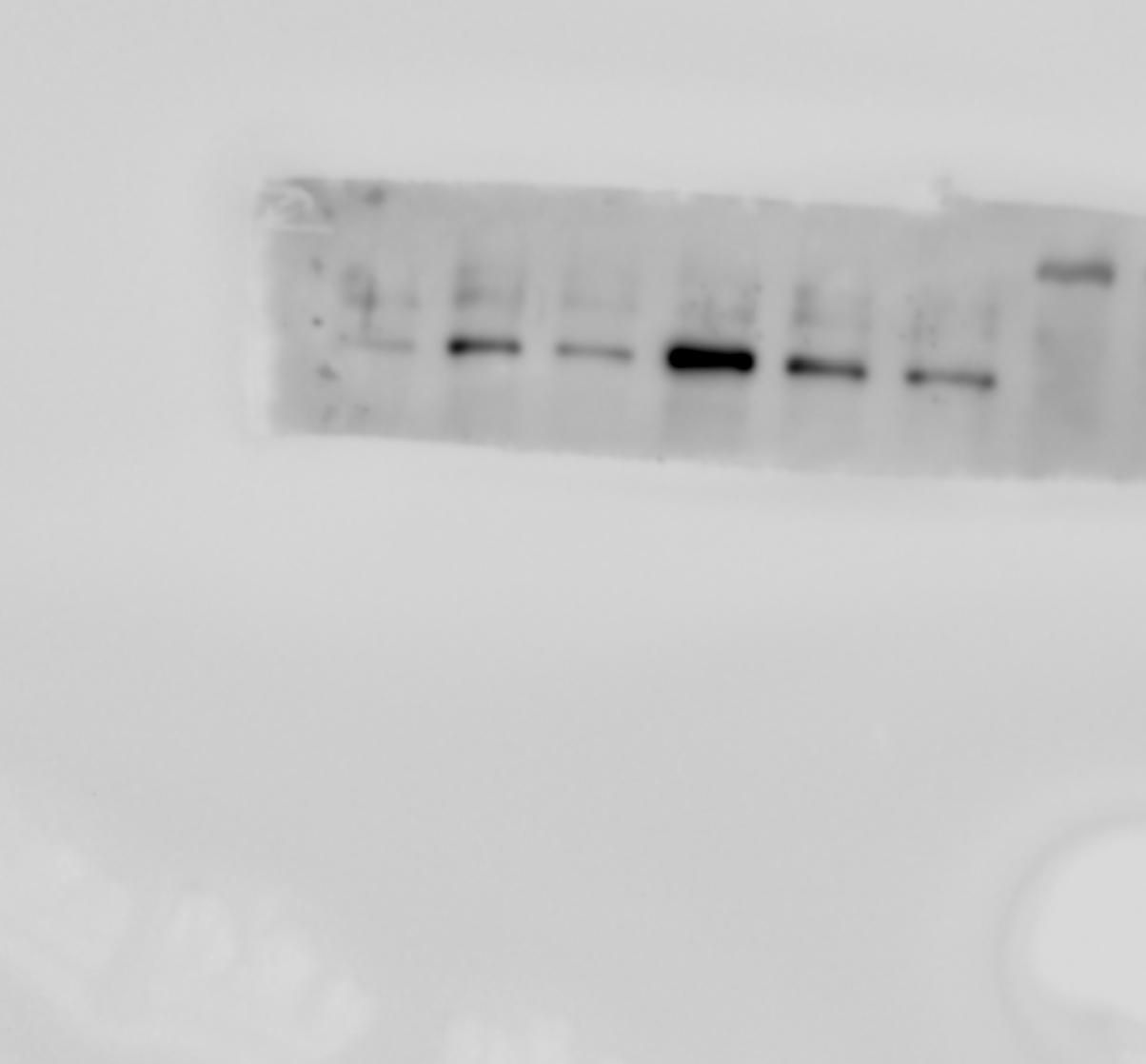

Supplement: Supplementary file 2 [file Data_Sheet_2.ZIP › WB/Figure 4/p65/1.tif]

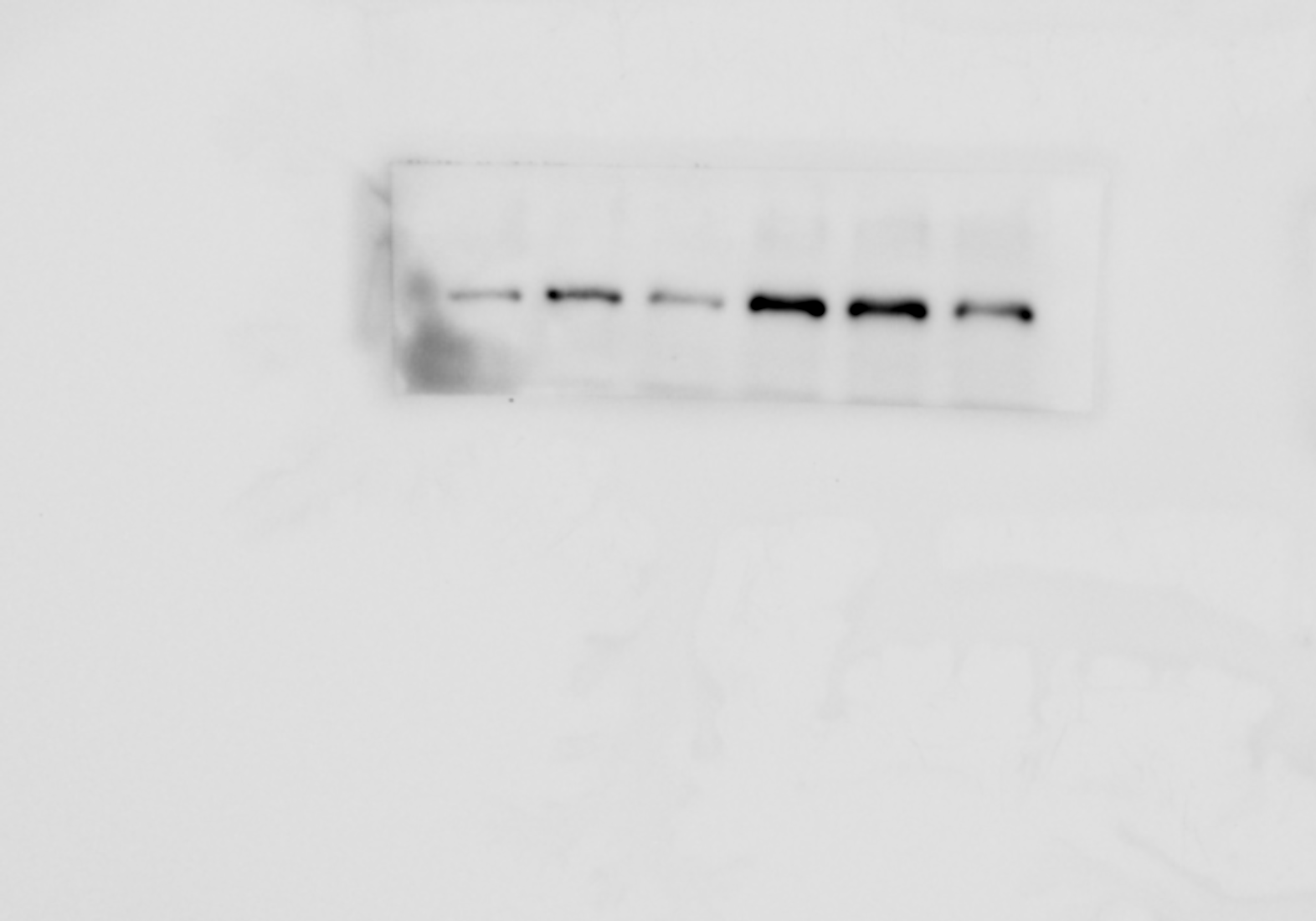

Supplement: Supplementary file 2 [file Data_Sheet_2.ZIP › WB/Figure 4/p65/2.tif]

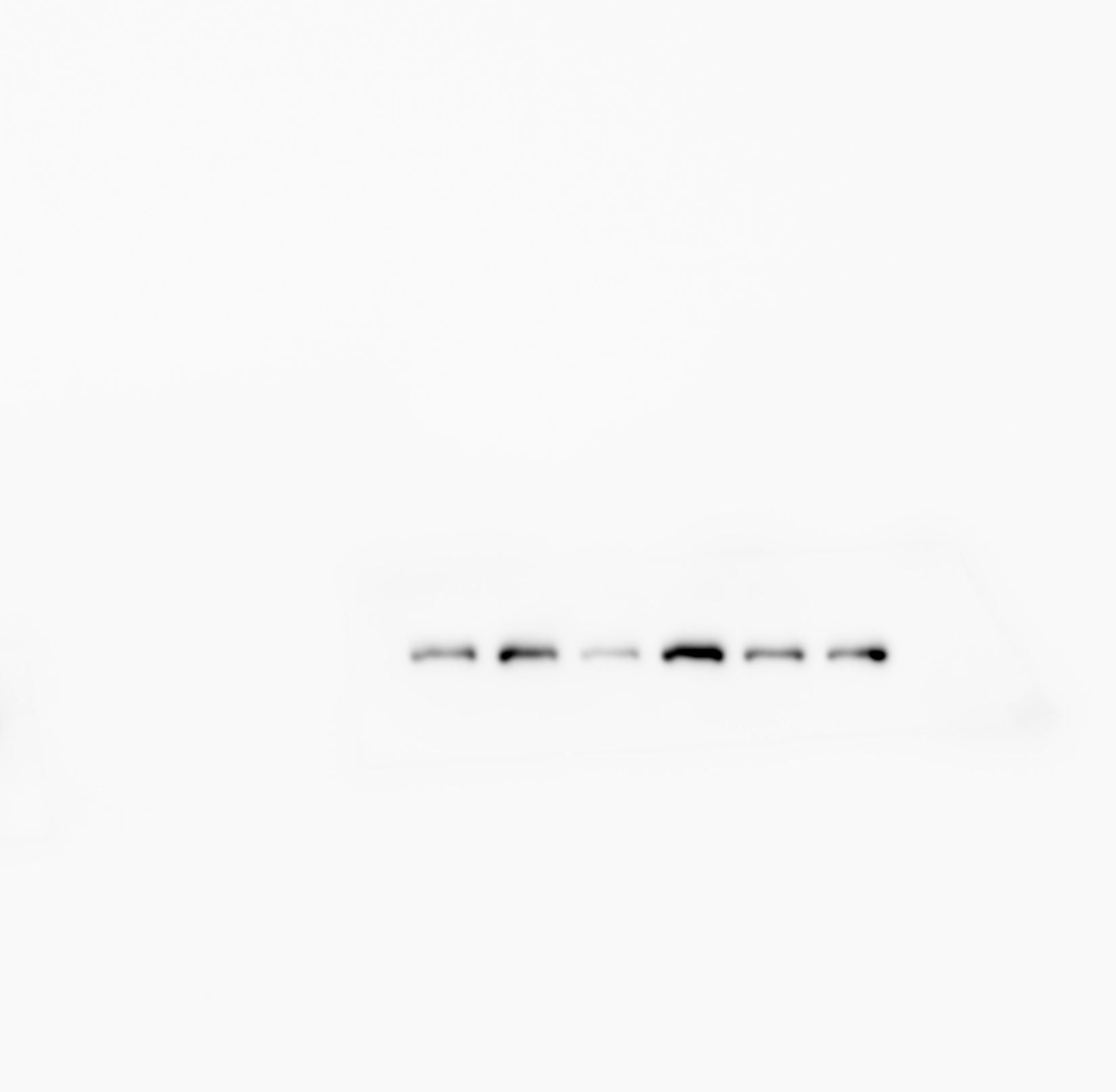

Supplement: Supplementary file 2 [file Data_Sheet_2.ZIP › WB/Figure 4/p65/3.tif]
