# Supplementary material for: Chronic Intermittent Hypoxia Reduces the Effects of Glucosteroid in Asthma via Activating the p38 MAPK Signaling Pathway
Source: Front Physiol. 2021 Aug 27;12:703281. doi: 10.3389/fphys.2021.703281 (PMC8430218; doi:10.3389/fphys.2021.703281)
Supplement: Supplementary file 2 [file Data_Sheet_2.ZIP › WB/uncropped blot.docx]

Figure 3

p-p38


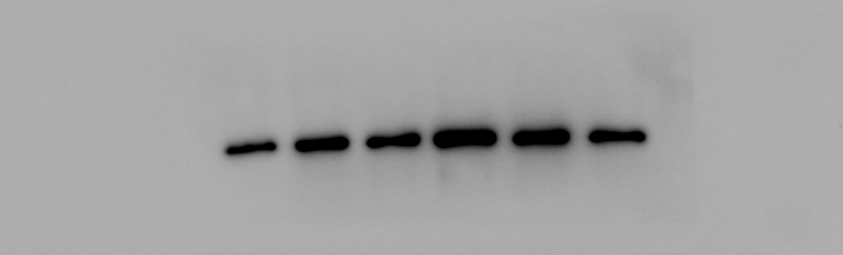


34kDa

p38


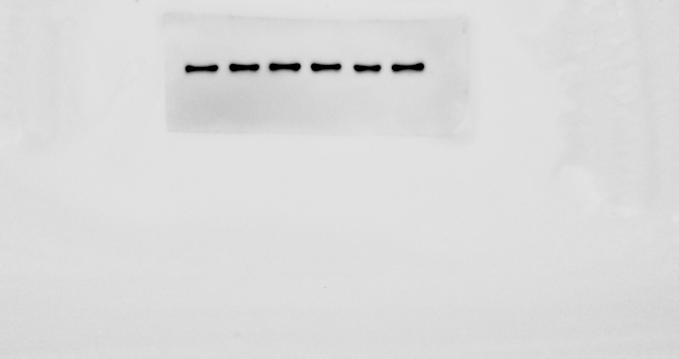


41kDa

MKP-1


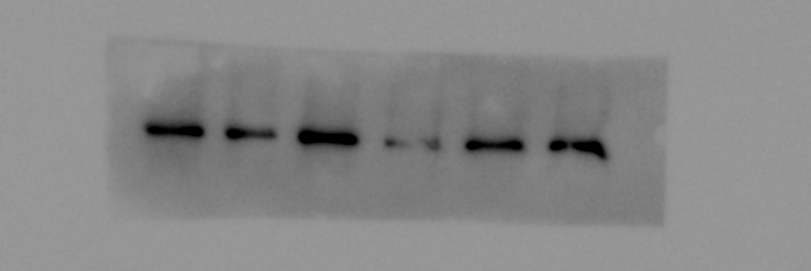


49kDa

HO-1


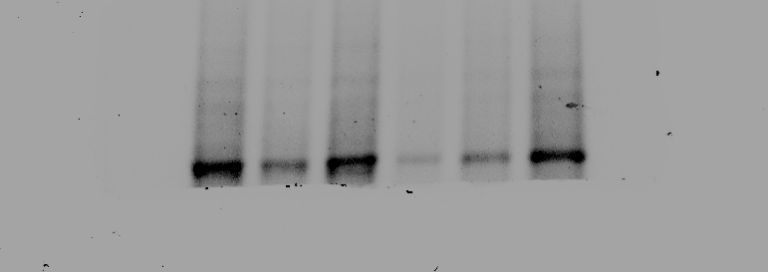


33kDa

ACTIN


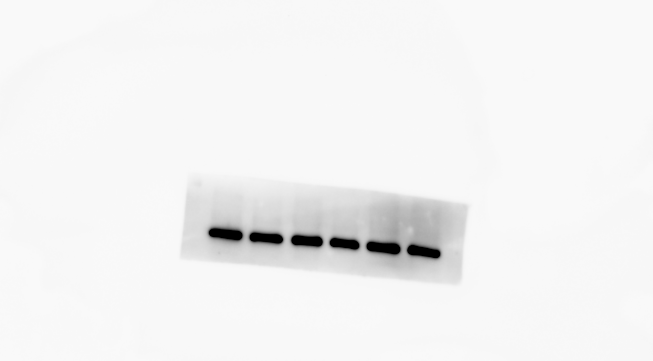


42kDa

P65


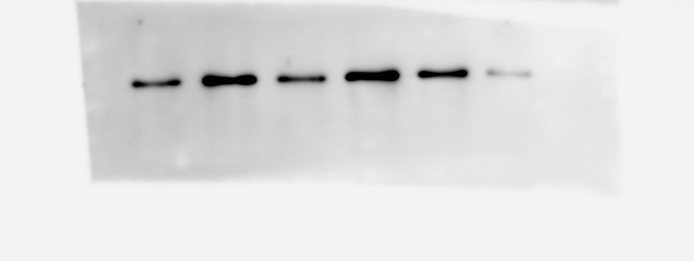


65kDa

LAMINB


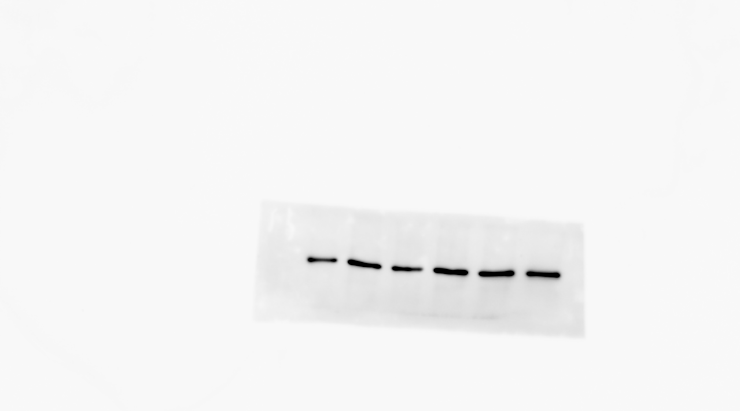


70kDa

Figure 4

p-p38


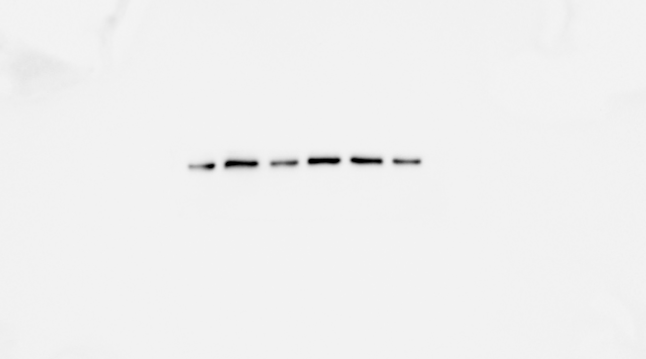


34kDa

p38


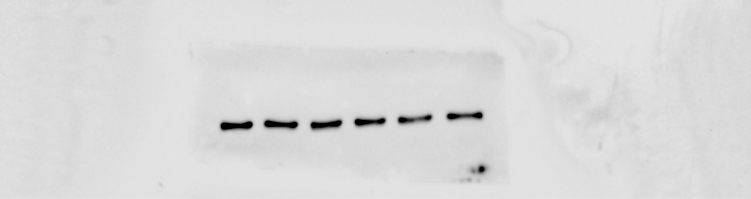


41kDa

MKP-1


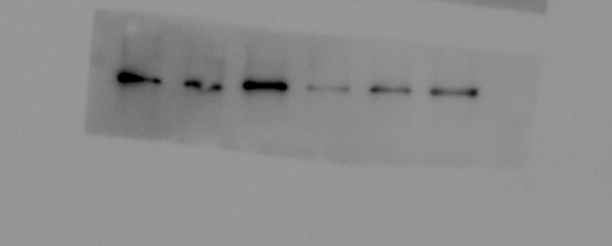


49kDa

HO-1


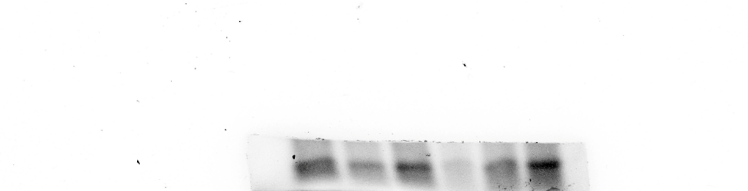


33kDa

ACTIN


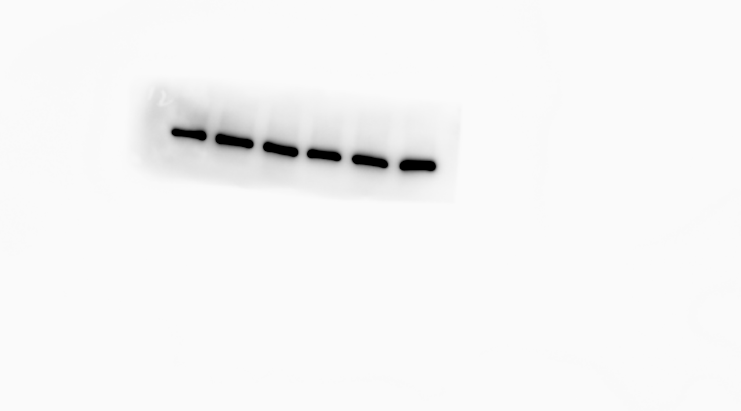


42kDa

P65


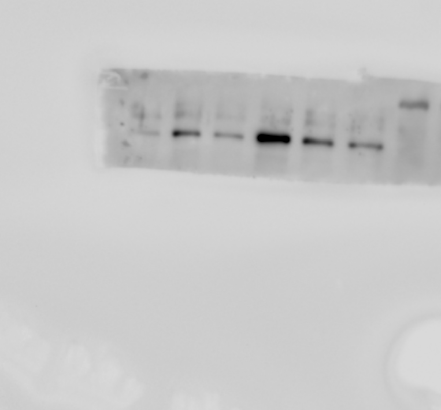


65kDa

LAMINB


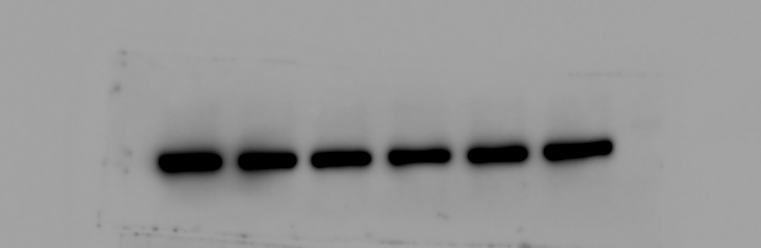


70kDa
